# d-cysteine impairs tumour growth by inhibiting cysteine desulfurase NFS1

**DOI:** 10.1038/s42255-025-01339-1

**Published:** 2025-08-12

**Authors:** Joséphine Zangari, Oliver Stehling, Sven A. Freibert, Kaushik Bhattacharya, Florian Rouaud, Veronique Serre-Beinier, Kinsey Maundrell, Sylvie Montessuit, Sabrina Myriam Ferre, Evangelia Vartholomaiou, Vinzent Schulz, Karim Zuhra, Víctor González-Ruiz, Sahra Hanschke, Takashi Tsukamoto, Michaël Cerezo, Csaba Szabo, Serge Rudaz, Michal T. Boniecki, Miroslaw Cygler, Roland Lill, Jean-Claude Martinou

**Affiliations:** 1 Department of Molecular and Cellular Biology, Faculty of Sciences, University of Geneva, Geneva, Switzerland; 2 Institut für Zytobiologie am Zentrum für Synthetische Mikrobiologie Synmikro, Philipps-Universität Marburg, Marburg, Germany; 3 MPC Therapeutics, Geneva, Switzerland; 4 Division of Thoracic and Endocrine Surgery, Department of Surgery, Faculty of Medicine, University of Geneva, Geneva, Switzerland; 5 School of Pharmaceutical Sciences, University of Geneva, Geneva, Switzerland; 6 Institute of Pharmaceutical Sciences of Western Switzerland, University of Geneva, Geneva, Switzerland; 7 Department of Oncology, Microbiology and Immunology, Faculty of Science and Medicine, University of Fribourg, Fribourg, Switzerland; 8 Johns Hopkins Drug Discovery and Department of Neurology, Johns Hopkins University, Baltimore, MD USA; 9 INSERM, U1065, Équipe 12, Centre Méditerranéen de Médecine Moléculaire (C3M), Université Côte d’Azur, Nice, France; 10 Department of Biochemistry, Microbiology and Immunology, University of Saskatchewan, Saskatoon, Saskatchewan Canada; 11 Present Address: TransCure bioService, Archamps, France; 12 Present Address: Steinmühle—Schule & Internat, Marburg, Germany; 13 Present Address: Centro de Metabolómica y Bioanálisis (CEMBIO), Facultad de Farmacia, Universidad San Pablo-CEU, CEU Universities, Urbanización Montepríncipe, Boadilla del Monte, Spain

**Keywords:** Energy metabolism, Breast cancer, Biochemistry, Metabolism

## Abstract

Selective targeting of cancer cells is a major challenge for cancer therapy. Many cancer cells overexpress the cystine/glutamate antiporter xCT/CD98, an l-cystine transport system that strengthens antioxidant defences, thereby promoting tumour survival and progression. Here, we show that the d-enantiomer of cysteine (d-Cys) is selectively imported into xCT/CD98-overexpressing cancer cell lines and impairs their proliferation, particularly under high oxygen concentrations. Intracellular d-Cys specifically inhibits the mitochondrial cysteine desulfurase NFS1, a key enzyme of cellular iron–sulfur protein biogenesis, by blocking sulfur mobilization due to steric constraints. NFS1 inhibition by d-Cys affects all cellular iron–sulfur cluster-dependent functions, including mitochondrial respiration, nucleotide metabolism and maintenance of genome integrity, leading to decreased oxygen consumption, DNA damage and cell cycle arrest. d-Cys administration diminishes tumour growth of human triple-negative breast cancer cells implanted orthotopically into the mouse mammary gland. Hence, d-Cys could represent a simple therapy to selectively target those forms of cancer characterized by overexpression of xCT/CD98.

## Abstract (web summary)

Zangari et al. show that d-cysteine targets NFS1, thus affecting Fe–S cluster biogenesis and impairing tumour growth.

## Main

The stereochemistry of amino acids (aa) is fundamental to the structure and function of biological systems. Except for glycine, all proteinogenic aa possess a chiral α-carbon, giving rise to two enantiomeric forms: l-aa and d-aa. While l-aa are exclusively used in ribosomal protein synthesis, d-aa were historically considered rare and restricted primarily to bacterial cell walls^1^.

However, accumulating evidence has changed this view, revealing the presence of d-aa across all domains of life and implicating them in a wide array of physiological processes^2^. In mammals, for instance, d-serine and d-aspartate act as neuromodulators, regulating synaptic plasticity, neurogenesis and broader aspects of neurotransmission^3^. Among these, d-Cys has emerged as a particularly intriguing molecule. Initially identified in plants and bacteria where it is metabolized by d-Cys desulfhydrases in the context of stress response and signalling^4^, d-Cys has more recently been detected in mammalian systems. It is generated from l-Cys via serine racemase^5^ and plays a crucial role in maintaining neural progenitor cell homeostasis, suggesting a novel regulatory function in neurodevelopment^6^. In pancreatic beta cells, d-Cys has also been shown to modulate insulin secretion^5^. Moreover, in eukaryotic tissues such as the brain and kidney, d-Cys is oxidatively metabolized by d-amino acid oxidase (DAAO) and 3-mercaptopyruvate sulfurtransferase to produce hydrogen sulfide (H_2_S), a gasotransmitter with cytoprotective and signalling roles^7^. These findings underscore the biological relevance of cysteine chirality, which can lead to distinct metabolic fates and physiological outcomes.

In parallel, aa metabolism has been identified as a key determinant for the development of cancer. Many tumours upregulate aa transporters and metabolic enzymes to support rapid growth and adapt to nutrient-limited environments. Leveraging this metabolic reprogramming, targeting aa metabolism has emerged as a promising therapeutic avenue^8,9^. However, despite notable progress in understanding l-aa metabolism in cancer, the role of d-aa in tumorigenesis remains largely unexplored.

Here, we show that d-Cys selectively impairs proliferation of those cancer cell lines that exhibit high expression of the cystine/glutamate antiporter xCT/CD98, while sparing cells with low levels of xCT/CD98 and cystine uptake. Mechanistically, this selectivity arises from disruption of mitochondrial iron–sulfur (Fe–S) protein biogenesis, because d-Cys, imported via xCT/CD98, acts as an inhibitory decoy substrate for the cysteine desulfurase NFS1, thereby compromising all mitochondrial, cytosolic and nuclear Fe–S protein-dependent biological processes.

## Results

### Selective inhibition of cancer cell proliferation by d-Cys

**Fig. 1:**
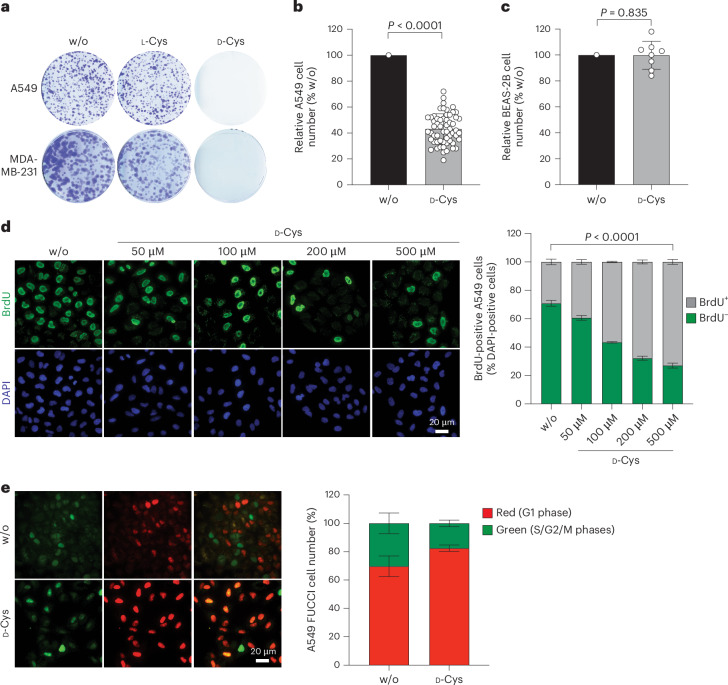
d-Cys impairs proliferation of certain tumour cells. **a**, Colony formation assays of A549 or MDA-MB-231 cells cultured for 15 days in medium supplemented with 100 µM d-Cys, 100 µM l-Cys or water (w/o). **b**, Time course of A549 cell proliferation in the absence (w/o) or presence of 500 µM d-Cys for 3 days (*n* = *58* biological replicates), analysed by Wilcoxon matched-pairs signed-rank test. **c**, Relative cell yield of BEAS-2B cells after 3 days in culture in the absence (w/o) or presence of 500 µM d-Cys; *n* = 8, paired two-tailed Student’s *t*-test. **d**,**e**, Cell cycle analysis of A549 cells cultured in the presence of indicated concentrations of d-Cys, using BrdU immunofluorescence assay (**d**; w/o and 500 µM d-Cys, *n* = 3 biological replicates each; 50, 100 and 500 µM d-Cys, *n* = 2 biological replicates each; two-way analysis of variance (ANOVA) followed by Dunnett’s multiple-comparisons test) or the FUCCI assay (**e**; *n* = *2* biological replicates). All data are presented as the mean ± s.d. Representative images are shown. Source data

**Extended Data Fig. 1:**
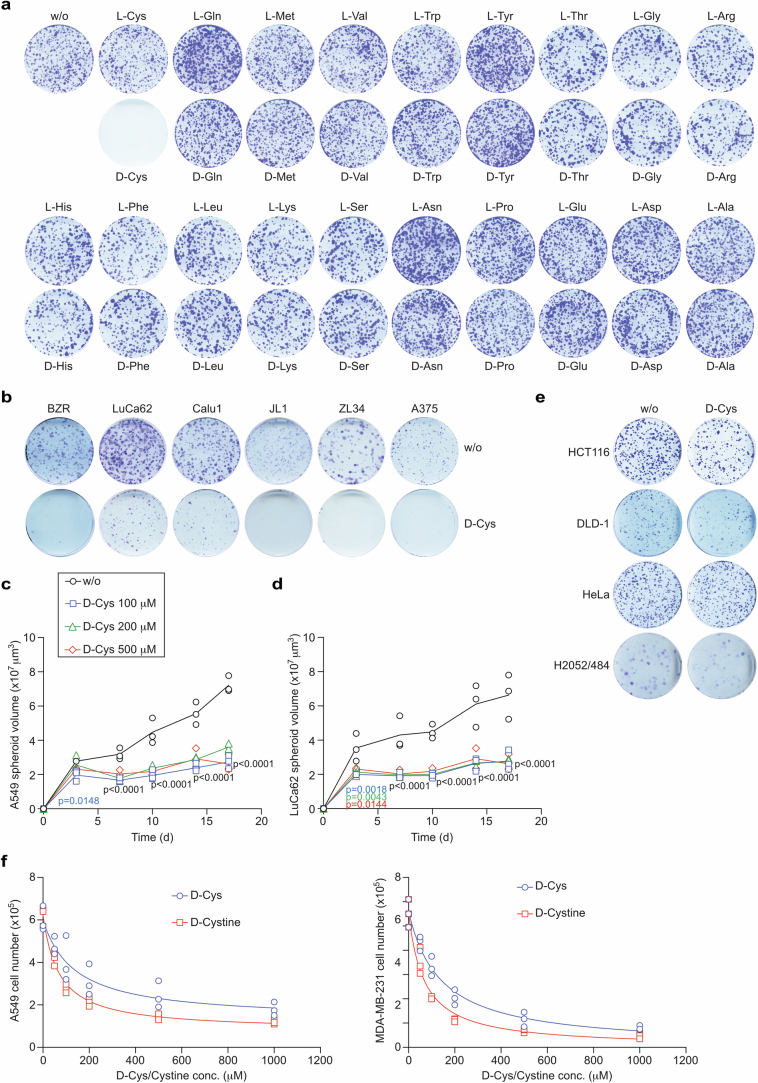
D-Cys impairs proliferation of a number of different cancer cell types grown under 2D or 3D conditions. **a**, Colony formation assay of A549 cells in the absence (w/o) or presence of either the L- or D-enantiomer of each proteinogenic amino acid. Each L- or D-amino acid was added to standard medium at similar concentrations as those already present in the medium. D-Cys was used at 100 µM. **b**, Colony formation assay for various cell types, including BZR, LuCa62, Calu1, JL1, ZL34, and A375 cells cultured in the absence (w/o) or presence of 100 µM D-Cys. **c**, **d**, Growth of A549 (**c**) and LuCa62 (**d**) spheroids in the absence (w/o) or presence of indicated D-Cys concentrations. All data are presented as mean ± SD of n = 3 biological replicates. Spheroid volumes at the indicated days of treatment were compared by 2-way ANOVA and Dunnett’s multiple comparisons test; **e**, Colony formation assays for HCT-116, DLD-1, HeLa and H2052/484 tumour cells in the absence (w/o) or presence of D-Cys. **f**, Determination of IC50 values using nonlinear fit, inhibitor conc *vs* response, for D-cystine or D-Cys for growth of A549 (left) or MDA-MB-231 cells (right). Source data

We tested whether d-aa would impact the growth and metabolism of A549 lung cancer cells. Either the l-enantiomer or the d-enantiomer of each proteinogenic aa was added at concentrations similar to those of l-aa already present in the basal medium. By using colony formation assays, we compared cell growth in the presence of l-aa or d-aa, and found that only d-Cys was able to prevent colony formation (Fig. 1a and Extended Data Fig. 1a). The presence of d-Cys also resulted in strong inhibition of A549 cell proliferation in regular two-dimensional (2D) cell cultures supplemented with 500 μM d-Cys for 3 days (Fig. 1b). Furthermore, d-Cys decreased the growth of numerous other cancer cells including the triple-negative breast cancer cell line MDA-MB-231 (Fig. 1a), as well as BZR, LuCa62 and Calu1 cells, the mesothelioma cells JL1 and ZL34 and melanoma A375 cells, irrespective of whether the cells were grown in 2D (Extended Data Fig. 1b) or three-dimensional (3D) cell cultures (Extended Data Fig. 1c,d). Interestingly, the normal lung bronchial epithelial cells BEAS-2B (Fig. 1c), as well as some other cancer cells including the colon adenocarcinoma HCT116 and DLD1, cervix cancer HeLa cells and malignant pleural mesothelioma H2052/484 cells (Extended Data Fig. 1e) were less or not at all sensitive to d-Cys, thus showing a high cell-type and aa specificity of this toxic effect.

Cell cycle analysis of d-Cys-treated A549 cells using bromodeoxyuridine (BrdU) immunolabelling or the FUCCI cell cycle sensor^10^ showed not only fewer BrdU-positive cells in d-Cys-treated cultures than in control cultures, but also an increased proportion of cells in the G1 phase of the cell cycle suggesting that d-Cys decreases cell proliferation through a cell cycle arrest in G1 (Fig. 1d,e).

Recent studies reported that d-Cys partially rescues growth in l-Cys-depleted cells, delaying cell death for at least 24 h, although cells ceased proliferating after extended cultivation as observed above^11^. We also observed a partial d-Cys rescue effect for l-Cys-depleted A549 cells (~30% relative to l-Cys supplementation; Supplementary Fig. 1a) and hypothesized that this effect could stem from the reducing power of d-Cys. Supporting this, continuous treatment with the reductant tris(2-carboxyethyl)phosphine (TCEP; 125–500 µM) restored cell growth almost as effectively as 500 µM d-Cys (Supplementary Fig. 1b). However, higher TCEP concentrations decreased this effect, likely due to reductive stress. We conclude that d-Cys provides only a transient reductive benefit to l-Cys-depleted cells, alongside its long-term inhibitory action.

Due to cysteine’s tendency to oxidize into cystine, the predominant extracellular form is l-cystine, both in vivo (within plasma and extracellular spaces^12,13^) and in vitro, even though the oxidation process in solution is slow (Supplementary Fig. 1c). Cells mainly take up l-cystine via the xc^−^ system that consists of the antiporter xCT (encoded by *SLC7A11*, exchanging cystine with glutamate) and its chaperone CD98 (encoded by *SLC3A2*)^14^. We compared the inhibitory effects of d-Cys and d-cystine on cell proliferation. The IC_50_ values for d-cystine were 67 ± 15 µM in MDA-MB-231 cells and 71 ± 27 µM in A549 cells, compared with 140 ± 22 µM and 154 ± 69 µM for d-Cys, respectively (*n* = 3; Extended Data Fig. 1f). These results confirm that both forms of d-Cys inhibit cell proliferation, with d-Cys showing twofold weaker inhibition. For most experiments, d-Cys was used due to its higher solubility compared with d-cystine.

### Cellular acquisition and toxicity of d-Cys mediated by xCT

**Fig. 2:**
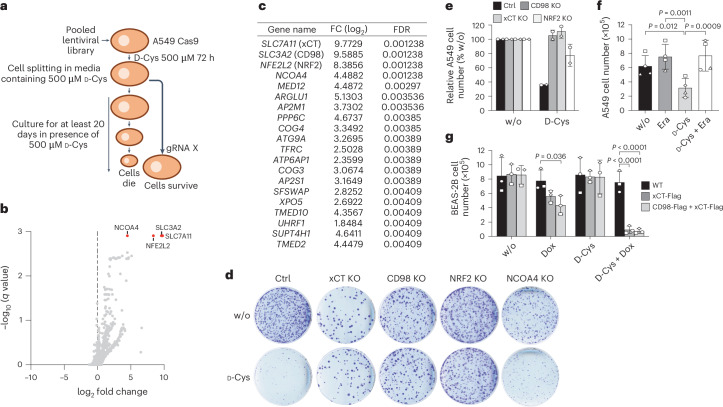
d-Cys is imported through xCT/CD98. **a**, Outline of the pooled guide RNA (gRNA) depletion screen. **b**,**c**, Volcano plot representation of the screen hits after cell growth in the presence or absence of 500 mM d-Cys. *q* values were calculated using MAGeCK, which includes a correction for multiple comparisons. The most notable hits required for d-Cys toxicity are shown in **c**. See also Supplementary Table 1 for detailed results. FC, fold change; FDR, false discovery rate. **d**, Colony formation assays of control (Ctrl) A549 cells and indicated A549 KO cells in the absence (w/o) or presence of 100 μM d-Cys. **e**, Relative cell yield of control A549 cells and indicated A549 knockout (KO) 2D cell cultures grown for 72 h in the absence (w/o, set to 100%) or presence of 500 μM d-Cys, *n* = *2* biological replicates (consisting of three technical replicates each). **f**, Effects of erastin (Era) and d-Cys on A549 cell yield after 72 h of culture (*n* = 4 biological replicates), analysed by two-way ANOVA followed by Tukey’s multiple-comparisons test. **g**, BEAS-2B cells inducibly overexpressing xCT-Flag alone or together with CD98-Flag (Extended Data Fig. 3c) following doxycycline (Dox) addition were cultured in the absence (w/o) or presence of 500 μM d-Cys for 72 h and counted (*n* = *3* biological replicates), analysed by two-way ANOVA followed by Tukey’s multiple-comparisons test. Data in **e**–**g** are presented as the mean ± s.d. Source data

**Extended Data Fig. 2:**
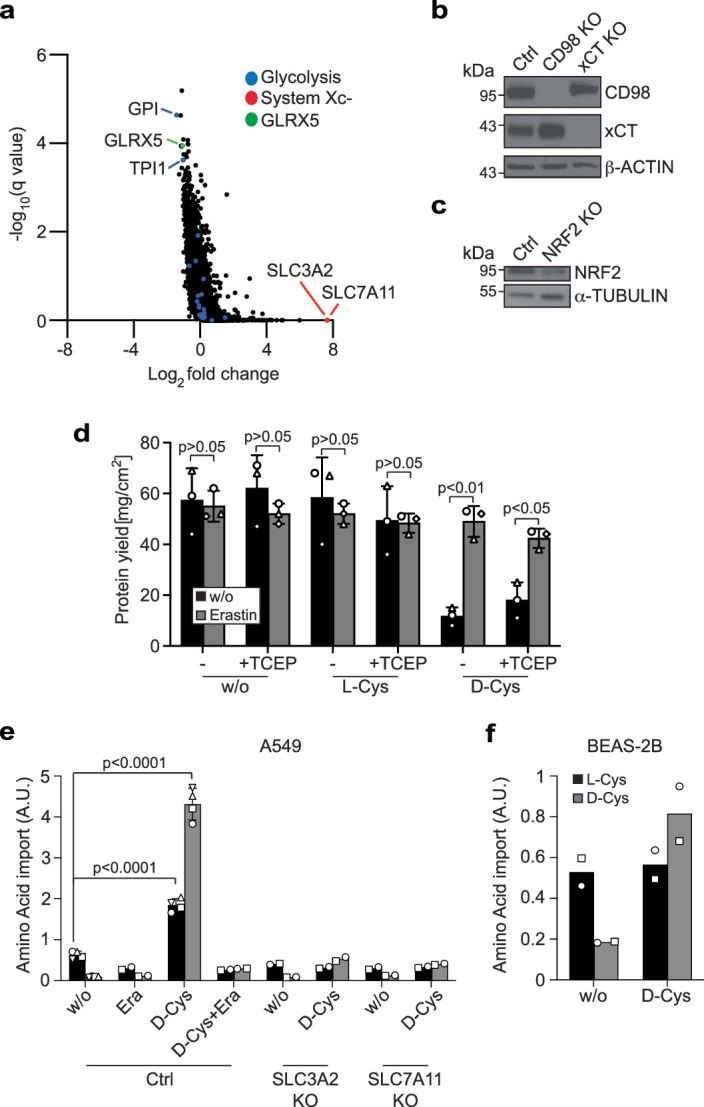
Genome-wide CRISPR/Cas9 knockout screening identifies genes increasing the D-Cys toxicity in A549 cells. **a**, Volcano plot showing the most significant proteins whose depletion boosts D-Cys toxicity. Among the top hits are several glycolytic genes and the mitochondrial ISC protein glutaredoxin (GLRX5); SLC3A12 and SLC7A11 (red bars) are mentioned for comparison. Q values were calculated using MAGeCK, which includes a correction for multiple comparisons. See also Supplementary Table 2 for detailed results. **b**, **c**, Validation of CRISPR/Cas9 KO of SLC3A2 (CD98), SLC7A11 (xCT), or NFE2L2 (NRF2) genes in A549 cells by immunoblotting of corresponding proteins (molecular masses in parentheses). Representative blots of n = 3 (**b**) and n = 2 (**c**) biological replicates. **d**, A549 cells were cultured in full culture medium in the absence (–) or presence of the reductant TCEP (375 μM), in the absence (Ctrl) or presence of the xCT inhibitor erastin (1 μM). Samples either did not receive additional Cys supplementation (w/o), or were supplemented with 500 μM L-Cys or D-Cys for a total of three days as indicated. Cells were harvested and total protein yield was determined (mean ± SD). The efficiency of erastin treatment was analyzed by 2-way repeated measures ANOVA and Bonferroni posttest; symbols indicate matching samples of n = 3 biological replicates. **e**, Wild-type or knock-out (KO) A549 cells were cultured for three days in full culture medium in the presence of erastin (Era) and/or 500 μM D-Cys as indicated. Intracellular L-Cys and D-Cys levels were determined as a measure of cellular Cys import. Results are mean ± SD of n = 3 biological replicates for cells cultured in the presence (D-Cys) or absence (w/o) of D-cys; n = 2 biological replicates (consisting of 3 technical replicates each) for the other conditions. Unpaired two-tailed Student’s t-tests were used for comparison between cells cultured with and without (w/o) D-Cys. **f**, BEAS-2B cells were cultured for three days in full culture medium in the absence or presence of 500 μM D-Cys as indicated, and the cellular L- and D-Cys content was determined in n = 2 experimental series as a measure of Cys import. Source data

**Extended Data Fig. 3:**
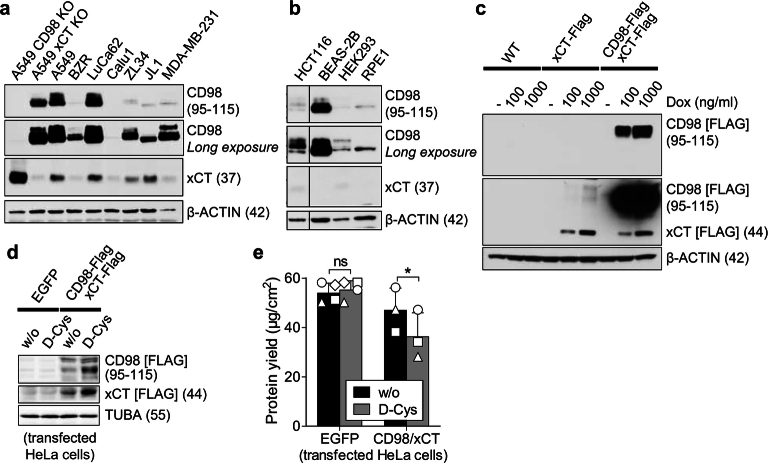
xCT (*SLC7A11*) and CD98 (*SLC3A2*) expression is associated with D-Cys sensitivity. **a**, **b**, Immunoblotting of xCT and CD98 in various cell types that display sensitivity (**a**) or resistance (**b**) to D-Cys. **c**, Doxycycline-induced (co-)expression of C-terminally FLAG-tagged xCT and CD98 was analysed by anti-FLAG immunoblotting in BEAS-2B cells stably transduced by lentiviral infection. **d**, HeLa cells were transiently transfected to co-express C-terminally FLAG-tagged xCT and CD98, or the reference protein EGFP as indicated, cultured in absence (w/o) or presence of D-Cys for three days, and analysed by anti-FLAG immunoblotting. **e**, Protein yield of HeLa cell cultures from (**d**). Data are presented as mean ± SD; *P < 0.05; ns, not significant. Comparison by 2-way repeated measures ANOVA and Bonferroni posttest; symbols indicate matching samples of n = 4 biological replicates (one series lacks xCT/CD98 values). Representative blots of n = 4 (**a**, **b**), n = 1 for (**c**) and n = 4 (**d**) biological replicates are shown. γ-actin and β-tubulin (TUBA) were used as loading controls (molecular masses in parentheses). Source data

To elucidate the mechanisms by which d-Cys impaired proliferation of cancer cells, we performed a CRISPR–Cas9 knockout screen (Fig. 2a). This screen identified the two components of the xc^−^ system, xCT and CD98, as well as NRF2 (encoded by *NFE2L2*; nuclear factor erythroid 2-related factor 2)^15^ and NCOA4 as top candidates essential for d-Cys toxicity in A549 cells (Fig. 2b,c and Supplementary Table 1). Conversely, the screen also identified several glycolysis-related genes whose depletion sensitized cells to d-Cys toxicity (Extended Data Fig. 2a and Supplementary Table 2), underscoring an essential role of glycolysis in cell survival under d-Cys exposure (see below). The top essential candidate genes implicated in d-Cys-mediated growth inhibition were individually validated by immunoblotting of dedicated CRISPR–Cas9 knockout extracts (Fig. 2d,e and Extended Data Fig. 2b,c). The suggested role of the xCT antiporter in mediating d-Cys toxicity was further confirmed pharmacologically using its inhibitor erastin, which effectively restored cell growth (Fig. 2f). Importantly, erastin attenuated d-Cys-induced growth inhibition both in the presence and absence of TCEP (Extended Data Fig. 2d), supporting the notion that xCT can import both d-cystine and d-Cys, fitting to a previous suggestion for the l-enantiomers^16^. Supporting the crucial role of xCT in d-Cys toxicity, all d-Cys-sensitive cancer cell lines were found to express both xCT and CD98, albeit at different levels (Extended Data Fig. 3a), whereas xCT expression was barely detectable in d-Cys-insensitive cells (Extended Data Fig. 3b). Consistent with this correlation, doxycycline-inducible overexpression of both xCT and CD98 in BEAS-2B cells was sufficient to render them highly sensitive to d-Cys (Fig. 2g and Extended Data Fig. 3c). Similar results were obtained for HeLa cells (Extended Data Fig. 3d,e).

All these findings align with the identification of NRF2 as another top mediator of d-Cys toxicity in the CRISPR–Cas9 knockout screen (Fig. 2a–c). NRF2 is a master regulator of the cellular redox state and, importantly, has been shown to regulate expression of xCT^17^. Knockout of *NFE2L2* (encoding NRF2) in A549 cells (Extended Data Fig. 2c) reduced expression of xCT by 87.2% ± 0.3% leading to decreased sensitivity to d-Cys (Fig. 2d,e).

The cellular content of d-Cys was measured using our recently developed chiral chromatography method for d-Cys quantification^18^. In xCT-expressing A549 cells, d-Cys sensitivity correlated with xCT-mediated, erastin-sensitive uptake of d-Cys (Extended Data Fig. 2e). CRISPR–Cas9 knockout of either SLC7A11 (xCT) or SLC3A2 (CD98) blocked d-Cys accumulation, confirming xCT dependency. In contrast, d-Cys-insensitive BEAS-2B cells exhibited low levels of d-Cys (Extended Data Fig. 2f). Together, these findings suggest that xCT-mediated d-Cys uptake drives cancer cell toxicity.

**Fig. 3:**
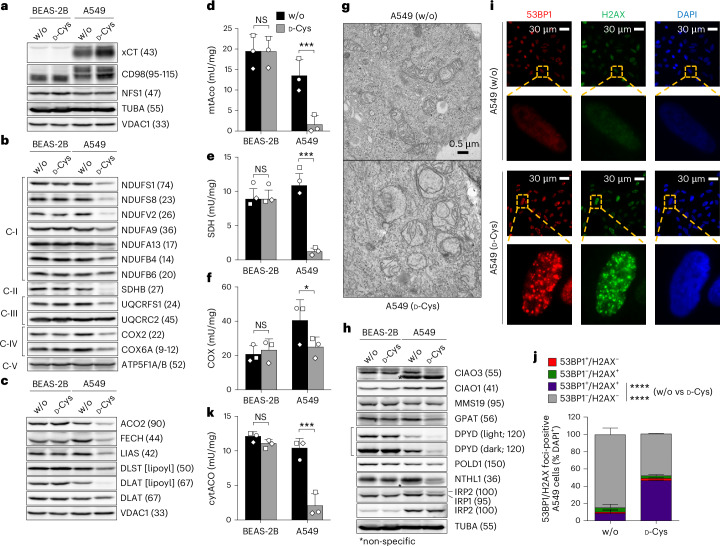
d-Cys induces severe cellular Fe–S protein defects. **a**–**k**, BEAS-2B and A549 cells were cultured in the absence (w/o) or presence of 500 µM d-Cys for a total of 3 days and analysed. **a**–**c**, Cell samples were immunoblotted against xCT and CD98 subunits, the indicated mitochondrial proteins or the lipoyl cofactor. TUBA, VDAC1 and ATP5F1A/ATP5F1B served as loading controls. **d**–**f**, Mitochondria-containing organellar fractions obtained by digitonin-based cell separation were analysed for the specific enzyme activities of mitochondrial aconitase (mtAco) (**d**), succinate dehydrogenase (SDH, respiratory complex II) (**e**) and cytochrome *c* oxidase (COX, respiratory complex IV) (**f**). Comparison by two-way repeated-measures ANOVA and Bonferroni post-test; symbols indicate matching samples of *n* = 3 biological replicates. **g**, Electron microscopy of mitochondria from A549 cells cultured for 3 days in the absence (w/o) or presence of 500 µM d-Cys. **h**, Cell samples were analysed by immunoblotting against the indicated cytosolic and nuclear proteins. Alpha-tubulin (TUBA) served as the loading control. **i**,**j**, Immunofluorescence analysis of 53BP1 and γ-H2AX to assess DNA damage in A549 cells cultured in the absence (w/o) or presence of d-Cys (**i**). The percentage of 53BP1-positive or γ-H2AX-positive cells was determined (**j**), *n* = 2 biological replicates. **k**, Cytosolic fractions obtained by digitonin-based cell separation were analysed for the specific enzyme activity of cytAco (IRP1); comparison by two-way repeated-measures ANOVA and Bonferroni post-test; symbols indicate matching samples of *n* = 3 biological replicates. Representative blots and images of *n* = 3 biological replicates are shown. Observed molecular masses in immunoblots are indicated in parentheses. C-I to C-V, OXPHOS complexes I–V. All data are presented as the mean ± s.d.; *P < 0.05; ***P < 0.001; ****P < 0.0001; NS, not significant. Source data

One possible explanation for d-Cys-mediated toxicity is the depletion of intracellular l-Cys and/or glutathione. However, d-Cys treatment unexpectedly led to a doubling of intracellular l-Cys levels, an effect that was abrogated by the xCT inhibitor erastin (Extended Data Fig. 2e). This increase may be attributed to the formation of mixed disulfides between l-Cys and d-Cys in the culture medium, which are co-imported via xCT, as previously proposed^13^, or to an upregulation of xCT expression in d-Cys-treated cells (Fig. 3a). Importantly, the glutathione levels remained unchanged in d-Cys-treated cells (Supplementary Table 3) ruling out l-Cys or glutathione depletion as potential causes of d-Cys toxicity.

### d-Cys impairs cellular Fe–S proteins

**Extended Data Fig. 4:**
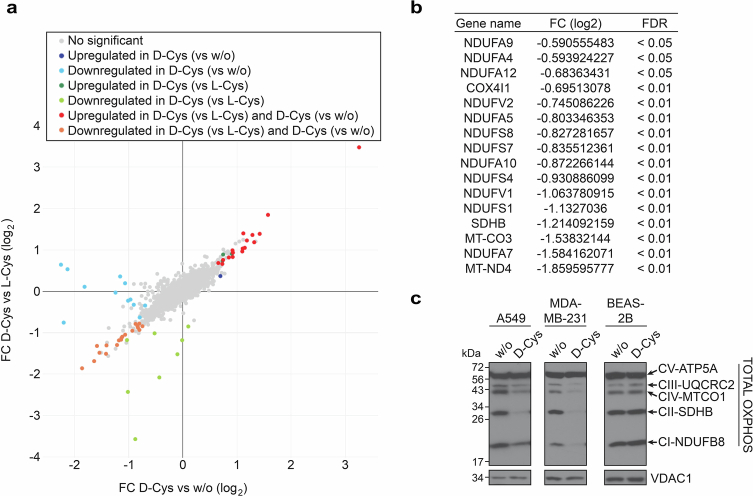
Proteomic analysis of A549 cells cultured in the presence or absence of D-Cys or L-Cys. A549 cells were cultured for three days in DMEM containing 200 μM L-cystine (w/o) or in the same medium supplemented with either 500 μM D-Cys or L-Cys, and a proteomic analysis was performed. **a**, Volcano plot showing proteins that vary among the different conditions. Different comparisons have been made according to the protein colour code shown in the box. **b**, List of the most downregulated mitochondrial proteins in D-Cys treated cells (orange dots from (**a**)). See also Suppl. Table S2 for a more detailed list of proteins analysed and their comparison among the different culture conditions. **c**, Immunoblot analyses of representative OXPHOS protein subunit levels in indicated cell lines cultured in the absence (w/o) or presence of D-Cys. VDAC1 served as loading control. Representative blots of n = 4 (for A549 cells) and n = 2 (for MDA-MA-231 and BEAS-2B cell) biological replicates are shown. Source data

Proteomic analysis of A549 cells treated with 500 µM d-Cys resulted in a depletion of subunits from respiratory chain complexes (RCCs) I, II and IV, compared with cells cultured in standard or l-Cys-supplemented medium (Extended Data Fig. 4a,b and Supplementary Table 4). Immunoblotting confirmed the d-Cys-induced diminution of RCC components in xCT-expressing A549 and MDA-MB-231 cells, but not in xCT-deficient BEAS-2B cells (Fig. 3a,b and Extended Data Fig. 4c). Because the most severely affected subunits in d-Cys-sensitive A549 cells contain Fe–S clusters, we hypothesized that the mitochondrial Fe–S cluster assembly (ISC) system might be a key target of d-Cys toxicity. This essential pathway critically depends on the cysteine desulfurase complex NFS1–ISD11–ACP1 ((NIA)_2_), which utilizes l-Cys as a sulfur donor for the de novo biosynthesis of [2Fe–2S] clusters on the scaffold protein ISCU2 (ref. ^19^). A failure of Fe–S protein biogenesis (model in Supplementary Fig. 2) leads to non-functional apoproteins that are unstable and become degraded^20^. This behaviour would explain the d-Cys effect on RCC subunits.

d-Cys treatment did not affect NFS1 protein levels (Fig. 3a), but consistent with a suspected functional defect of the enzyme and impaired Fe–S cluster synthesis, the level and activity of the [4Fe-4S] enzyme ACO2 (Fig. 3c,d) as well as the amount of the Fe–S cluster binding subunit SDHB and the activity of RCC II (Fig. 3b,e) were severely decreased. As a control, the levels of the Fe–S cluster-independent RCC III subunit UQCRC2 and subunit ATP5F1 of complex V (Fig. 3b), as well as the enzyme activity of citrate synthase (Supplementary Fig. 3a), remained unaffected. We further evaluated the [4Fe-4S] radical SAM enzyme lipoyl synthase (LIAS)^21^, which catalyses the conversion of a protein-bound octanoyl moiety into the lipoyl cofactor in a [2Fe–2S] ferredoxin FDX1-dependent manner^22^. d-Cys treatment resulted in decreased LIAS protein levels in A549 but not BEAS-2B cells, suggesting impaired Fe–S maturation resulting in instability of the enzyme (Fig. 3c). A functional defect of LIAS was indicated by the severely diminished lipoylation of DLAT and DLST, the E2 subunits of pyruvate dehydrogenase and 2-ketoglutarate dehydrogenase, respectively, despite unchanged DLAT protein levels (Fig. 3c). The multiple detrimental effects of d-Cys treatment on enzymes connected to the citric acid cycle were reflected by metabolomic findings revealing a twofold accumulation of succinate and a 2.5-fold reduction in malate (Supplementary Table 5).

To test whether d-Cys treatment also affects mitochondrial [2Fe–2S] proteins, we examined ferrochelatase (FECH), the final enzyme of haem biosynthesis^23^ (Supplementary Fig. 2). d-Cys treatment markedly decreased FECH levels in A549 but not BEAS-2B cells (Fig. 3c). Similar findings were made for the Rieske Fe–S protein UQCRFS1 (Fig. 3b), together indicating impaired [2Fe–2S] cluster maturation. We also found a strong diminution in the levels of subunits COX2 and COX6A of RCC IV (Fig. 3b) as well as decreased COX enzyme activity following d-Cys treatment (Fig. 3f). This impairment is readily explained by the FECH-dependent and FDX1-dependent biosynthesis of the COX cofactors haem *a/a*_*3*_^22^, and/or the [4Fe-4S] cluster-containing METTL17-dependent assembly of the mitoribosome needed for translation of COX1, COX2 and COX3 subunits^24^.

**Extended Data Fig. 5:**
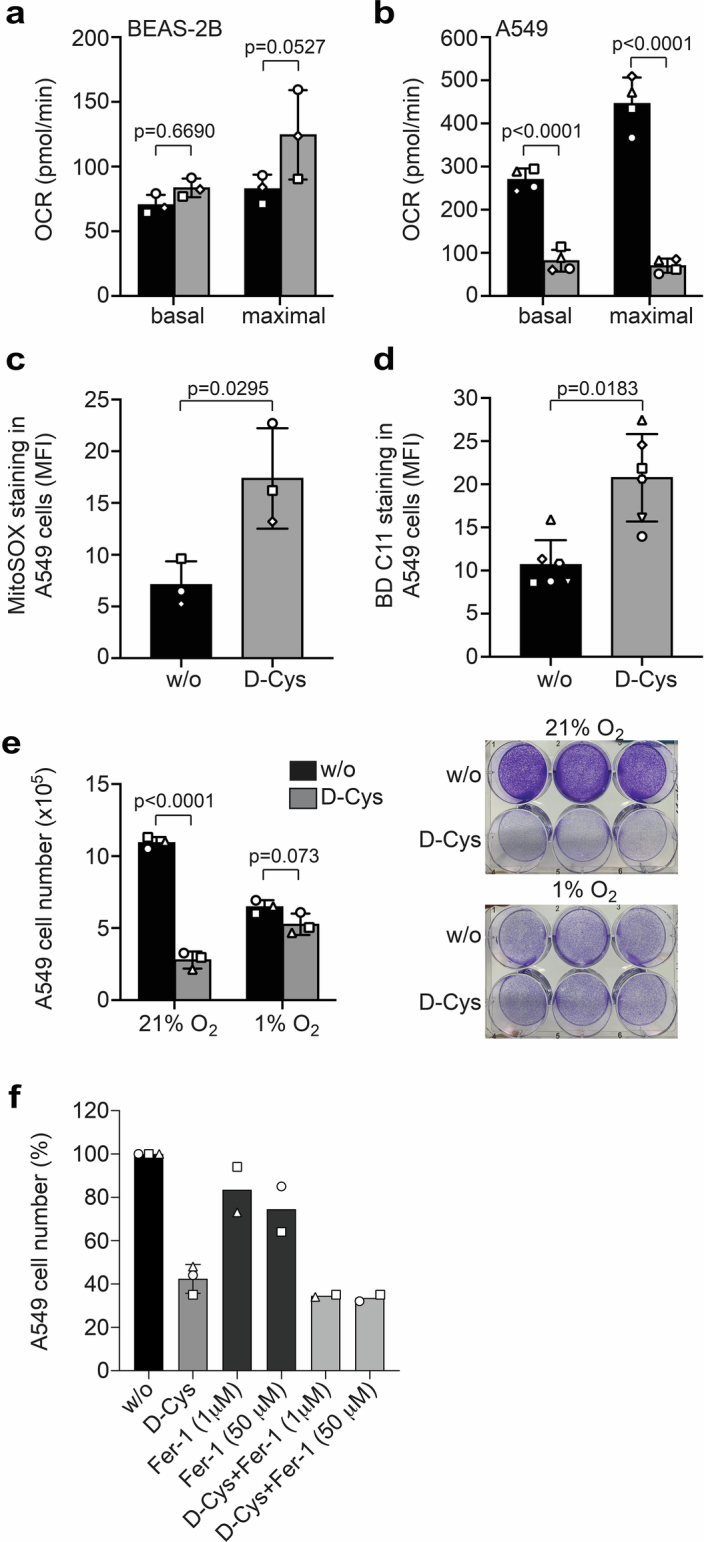
D-Cys decreases oxygen consumption rates and triggers ROS and lipid peroxidation in A549 cells. **a**, **b**, Basal and maximal oxygen consumption rates (OCR) of BEAS-2B (**a**) or A549 cells (**b**) cultured in the absence (w/o) or presence of 500 μM D-Cys for three days. Data of n = 3 (**a**) and n = 4 (**b**) biological replicates are presented as mean ± SD and were analysed by 2-way ANOVA followed by Sidak’s multiple comparisons test. **c**, **d**, Quantitation of FACS-analyses of ROS production in A549 cells cultured in the absence (w/o) or presence of 500 μM D-Cys for three days, using either MitoSOX (determination of mitochondrial ROS in n = 3 biological replicates) (**c**) or BD C11 (determination of lipid peroxidation in n = 6 biological replicates) (**d**). Mean fluorescence intensity (MFI) ± SD is shown; data were analysed by unpaired two-tailed Student’s t-test. **e**, A549 cells have been cultured under normoxia (21% oxygen, O_2_) or hypoxia (1% oxygen, O_2_) for 72 h, either in the absence (w/o) or presence of 500 mM D-Cys. Cells were then counted (left panel) or stained with crystal violet (right panel). Cell counts of n = 3 biological replicates are presented as mean ± SD and were analysed by unpaired two-tailed Student’s t-test. **f**, Lack of effect of ferrostatin-1 (Fer-1) on D-Cys-induced toxicity. A549 cells were cultured for 72 h with or without D-Cys and with the indicated concentrations of Fer-1 added freshly from day 1 to day 3. Following the 72 h culture period cells of n = 3 biological replicates were counted, and the results are presented as mean ± SD. The comparison was performed by an unpaired two-tailed Student’s t-test. Source data

The widespread defects in mitochondrial Fe–S proteins following d-Cys treatment were accompanied by reduced oxygen consumption, elevated reactive oxygen species and increased lipid peroxidation (Extended Data Fig. 5a–d). Notably, hypoxic growth conditions (1% oxygen) significantly diminished d-Cys toxicity (Extended Data Fig. 5e), closely resembling the hypoxia phenotypes observed in NFS1-deficient^25^ or frataxin (FXN)-deficient^26^ cells. Electron microscopy of d-Cys-treated A549 cells revealed profound changes of the mitochondrial morphology including distorted cristae and onion-like membranes (Fig. 3g), a phenotype reminiscent of NFS1-depleted mitochondria^27^. All these findings further support our hypothesis that d-Cys may target NFS1 function and elicit a general deficiency in mitochondrial Fe–S proteins.

The mitochondrial core ISC system including NFS1 is also critical for extra-mitochondrial Fe–S protein biogenesis, involving the cytosolic Fe–S protein assembly (CIA) machinery^27,28^ (Supplementary Fig. 2). In line with a mitochondrial core ISC defect following d-Cys treatment, the level of the Fe–S cluster-containing CIA protein CIAO3 (also known as IOP1 or NARFL), which facilitates [4Fe-4S] cluster transfer from the NBP35–CFD1 scaffold complex to the CIA targeting complex (CTC)^29–31^, was significantly reduced (Fig. 3h). In contrast, the Fe–S cluster-lacking CTC components CIAO1 and MMS19 remained unaffected. The d-Cys-dependent loss of CIAO3 was accompanied by decreased levels of multiple cytosolic and nuclear Fe–S proteins involved in nucleotide metabolism and genome integrity. This included glutamine phosphoribosyl pyrophosphate amidotransferase^32^, dihydropyrimidine dehydrogenase^33^, DNA polymerase delta (POLD1) and Nth-like DNA glycosylase 1 (NTHL1)^34–36^ (Fig. 3h).

To assess the impact of d-Cys treatment on DNA integrity, we measured the DNA damage markers 53BP1 and γ-H2AX. We found a substantial increase in nuclear foci, indicating that d-Cys compromised DNA integrity (Fig. 3i,j). This DNA damage, linked to dysfunction of NTHL1 and other Fe–S enzymes involved in DNA metabolism, along with impaired purine and pyrimidine metabolism (via glutamine phosphoribosyl pyrophosphate amidotransferase and dihydropyrimidine dehydrogenase inhibition), likely explains the G1 cell cycle arrest observed in d-Cys-treated cells (Fig. 1d,e). The severe cytosolic Fe–S protein defect by d-Cys was also evident from a decrease of cytosolic aconitase (cytAco) levels and enzyme activity, while lactate dehydrogenase activity remained unchanged (Fig. 3k and Supplementary Fig. 3b). A similar effect was previously observed following NFS1 depletion^27^. Because the apoform of cytAco functions as iron regulatory protein 1 (IRP1), this suggested a dysregulation of cellular iron metabolism, even though we note that IRP2 levels remained unchanged (Fig. 3h)^20^. Increased cellular iron levels were also suggested by our CRISPR–Cas9 knockout screen identifying the transferrin receptor (TFR1) and the ferritinophagy-related receptor NCOA4 as mediators of d-Cys toxicity (Fig. 2a–c and Supplementary Table 1). Altered iron levels and impaired mitochondrial respiration may explain the increased oxidative stress observed in d-Cys-treated A549 cells (Extended Data Fig. 5c,d). However, treatment with 500 µM TCEP did not ameliorate the d-Cys-induced growth and Fe–S enzyme defects, indicating that the toxic effect is caused mainly by reduced d-Cys (Supplementary Fig. 4). Likewise, ferrostatin-1, an iron-dependent lipid radical scavenger and ferroptosis inhibitor, failed to alleviate d-Cys toxicity (Extended Data Fig. 5f). This result is consistent with no detectable effects of ferrostatin-1 on NFS1-depleted MDA-MB-231 cells^25^. Together, these findings suggest that ferroptosis or lipid radicals are no decisive determinants for d-Cys-induced toxicity.

**Extended Data Fig. 6:**
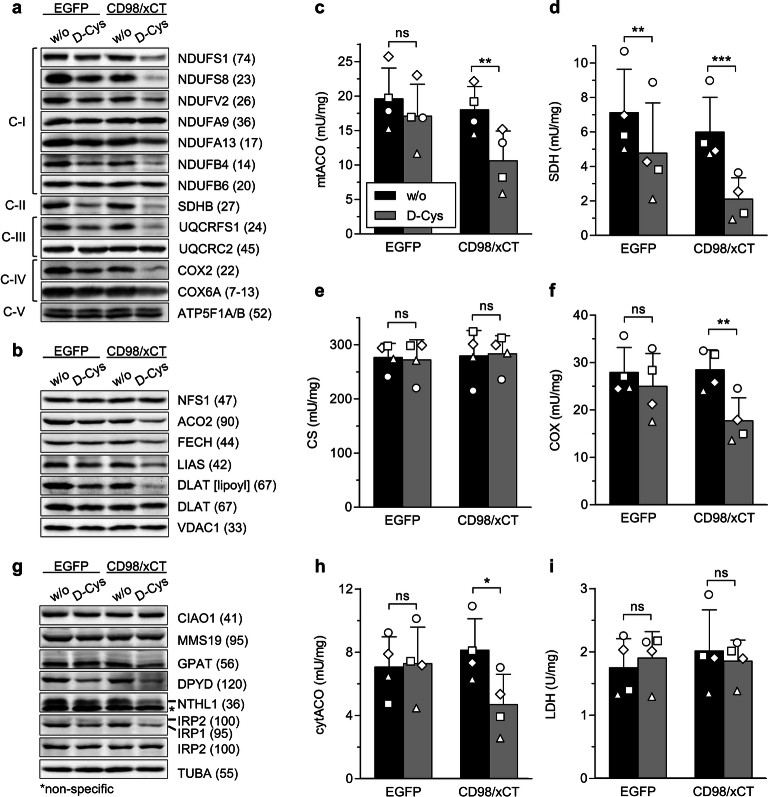
D-Cys induces Fe-S protein defects in xCT/CD98-overproducing HeLa cells. HeLa cells were transiently transfected to co-express C-terminally FLAG-tagged CD98 and xCT, or the reference protein EGFP as indicated. Cells were cultured in the absence (w/o) or presence of 500 μM D-Cys for three days (cf. Extended Data Fig. 3d, e). **a**, **b**, Cell extracts were analysed by immunoblotting of the indicated mitochondrial proteins or the lipoyl cofactor. ATP5F1A/B and VDAC1 served as loading controls. **c**–**f**, Mitochondria-containing organellar fractions, obtained by digitonin-based cell separation, were analysed for the specific enzyme activities of mitochondrial aconitase (mtAco), succinate dehydrogenase (SDH, respiratory complex II), citrate synthase (CS), and cytochrome c oxidase (COX, respiratory complex IV). **g**, Cell samples were analysed by immunoblotting of the indicated cytosolic nuclear proteins. α-tubulin (TUBA) served as loading control. **h**-**i**, Cytosolic fractions obtained by digitonin-based cell separation were analysed for the specific enzyme activities of cαytosolic aconitase (cytAco) and lactate dehydrogenase (LDH). Representative blots of n = 4 biological replicates are shown. Numbers in parentheses indicate observed molecular masses. C-I to C-V, OXPHOS complexes I to V. Data are presented as mean ± SD; *P < 0.05; **P < 0.01; ***P < 0.001; ns, not significant. Symbols indicate matching samples of n = 4 biological replicates. Source data

Overall, our results showed that d-Cys treatment of xCT/CD98-overexpressing A549 cells induced general defects in cellular Fe–S proteins. To further test the dependence of this effect on xCT/CD98, we used d-Cys-insensitive HeLa cells. Notably, following overexpression of xCT/CD98, these cells became sensitive to d-Cys, causing general Fe–S protein-related mitochondrial defects in, for example, energy conversion, haem and lipoyl cofactor formation (Extended Data Fig. 6a–f). Likewise, cytosolic and nuclear Fe–S proteins were decreased following d-Cys treatment (Extended Data Fig. 6g–i). Our data collectively identify NFS1 as the primary target of d-Cys (see also below). This conclusion is further supported by the exacerbation of d-Cys toxicity following CRISPR–Cas9-mediated depletion of the mitochondrial ISC protein glutaredoxin 5 (GLRX5; Extended Data Fig. 2a, Supplementary Table 2 and Supplementary Fig. 2), likely due to additional impairment of cellular Fe–S protein biogenesis.

### d-Cys does not support sulfur transfer within NFS1

**Fig. 4:**
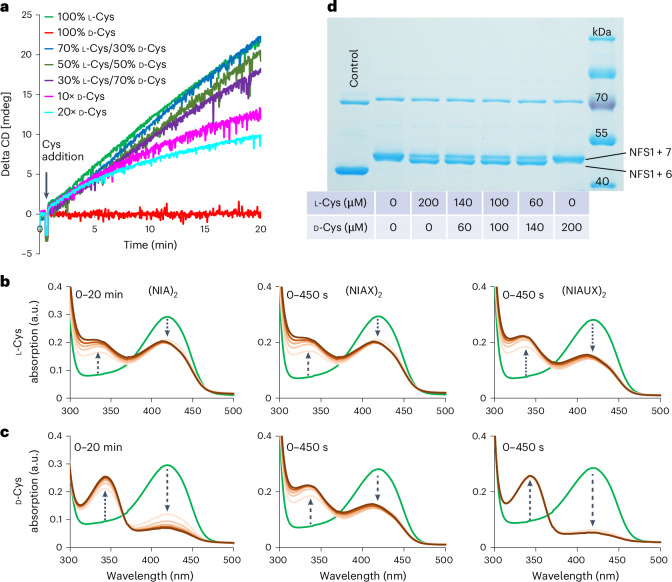
d-Cys does not support sulfur transfer within NFS1 during de novo [2Fe–2S] cluster biosynthesis. **a**, Enzymatic [2Fe–2S] cluster reconstitution by the core ISC complex on the ISCU2 scaffold using l-Cys (positive control), d-Cys and mixtures of both enantiomers as indicated. Final Cys concentration was 1 mM for all mixtures, except for 10× and 20× d-Cys (0.5 mM). **b**,**c**, Cys-ketimine generation on (NIA)_2_, (NIAX)_2_ and (NIAUX)_2_ complexes measured by UV/Vis spectroscopy. The increase in the absorption at 340 nm indicates the generation of the l-Cys-ketimine (**b**) and d-Cys-ketimine (**c**) at the expense of the internal aldimine absorbing around 416 nm. Nearly identical rates of Cys-ketimine formation were observed for l-Cys and d-Cys (for time courses see Extended Data Fig. 7b,c). (NIA)_2_ spectra were recorded every 4 min, while (NIAX)_2_ and (NIAUX)_2_ spectra were recorded every 90 s. **d**, Persulfidation of Cys381^NFS1^ (NFS1 + 6) in the presence of l-Cys or d-Cys or mixtures thereof at the indicated concentrations (for explanation of the assay see Supplementary Fig. 7; representative image of two biological replicates is shown). d-Cys did not enable any NFS1 persulfidation (NFS1 + 7). The left lane shows non-labelled NFS1 as a control. The band at 70 kDa is the *Escherichia* *coli* DnaK chaperone, which does not contain a persulfide. a.u., arbitrary units.

To further test our hypothesis that d-Cys targets NFS1, causing a general deficiency in cellular Fe–S protein biogenesis, we investigated the initial step of this complex process: the de novo synthesis of [2Fe–2S] clusters on the ISCU2 scaffold (Supplementary Fig. 2). This step requires the cysteine desulfurase complex (NIA)_2_, the regulatory protein FXN and the electron transport chain components FDX2, FDXR and NADPH^37,38^. Because NFS1 catalyses the sulfur release from l-Cys producing alanine and a persulfide (-SSH) on its active-site residue Cys381, the enzyme is a likely target of d-Cys. To biochemically investigate NFS1 function, we used a well-established enzymatic reconstitution system monitoring de novo [2Fe–2S] cluster formation on ISCU2 by circular dichroism (CD) spectroscopy under conditions that allow estimation of initial rates^37,39^. Following addition of d-Cys, no Fe–S cluster formation above background levels was observed, in stark contrast to addition of l-Cys, which served as a positive control (Fig. 4a). These findings indicate that the d-enantiomer did not support [2Fe–2S] cluster formation on ISCU2.

To assess the inhibitory potential of d-Cys, we used mixtures of d-Cys and l-Cys. As the ratio of d-Cys to l-Cys increased, a steady decrease in [2Fe–2S] cluster formation was observed, although notable inhibition required an excess of d-Cys (Fig. 4a). These results suggest that NFS1 preferentially interacts with l-Cys over d-Cys. To further explore this selectivity, we measured the apparent affinities of both enantiomers for the (NIA)_2_ complex using microscale thermophoresis. To prevent catalytic turnover during the measurements, we used the active-site variant NFS1-p.Cys381Ser, which cannot generate the Cys381^NFS1^-bound persulfide. Consistent with the required excess of d-Cys over l-Cys to obtain inhibitory effects on Fe–S cluster synthesis, we observed a 12-fold lower apparent affinity of (NIA)_2_ for d-Cys (*K*_d_^app^ = 25.6 µM) compared with l-Cys (*K*_d_^app^ = 2.15 µM; Supplementary Fig. 5). Collectively, these findings suggest that the cysteine desulfurase NFS1 binds d-Cys more weakly than l-Cys. However, when d-Cys is present in excess, the NFS1 enzymatic activity is inhibited, even though the enzyme still cannot utilize d-Cys as a substrate.

**Extended Data Fig. 7:**
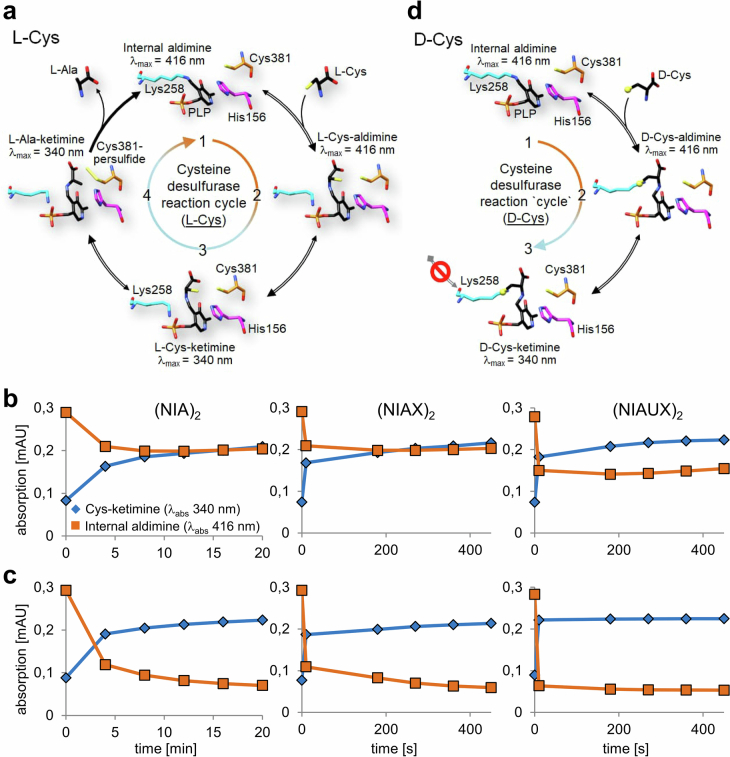
NFS1 can generate a D-Cys-ketimine intermediate but is unable to mobilise sulfur. **a**, Cartoon of the reaction cycle of the cysteine desulfurase NFS1 with L-Cys. The resting state of the enzyme comprises an internal (that is enzyme-bound) aldimine, by which the pyridoxal phosphate (PLP) cofactor is covalently bound to Lys258 as a Schiff base (1). Cys381 and His156 are positioned close to the PLP. Binding of free L-Cys creates an external (that is non-enzyme-bound) L-Cys-aldimine (2) which tautomerizes (catalysed by His156) to an external L-Cys-ketimine (3). The sulfur atom (yellow ball) can be released from L-Cys ketimine and transferred to Cys381, thereby creating an external L-Ala-ketimine and a persulfide (-SSH) on Cys381 (4). The resting state 1 of the enzyme with its internal aldimine is regenerated by rapid release of free L-Ala. Residue numbering is for human NFS1. The reaction scheme was adopted from refs. ^40,41^. **b**, **c**, Time courses of the generation of the L- and D-Cys-ketimine intermediate (absorption at 340 nm, blue) from the internal aldimine (416 nm, red) by the indicated (NIA)_2_ complexes. Data were taken from the spectra shown in Fig. 4b, c. **d**, Cartoon of the NFS1 reaction with D-Cys. The enzyme can efficiently and rapidly form the D-Cys-aldimine (2) and -ketimine intermediates (3), but for steric reasons cannot transfer the sulfur to Cys381, unlike with L-Cys (**a**). For 3D inspection of the models in parts **a**,**d**, a Chimera X session has been added as Supplementary File 1 (L_D-Cys_Cycle_final.X.py), using the following 3D structures: PDB ID: 7E6D, 6O13, 6O11, 7XEJ. refs. ^40,41^.

We next investigated which specific step of the NFS1 enzymatic reaction was blocked by d-Cys. Briefly, the reaction cycle of the pyridoxal phosphate (PLP)-dependent NFS1 (and related desulfurases) involves the conversion of the Lys258^NFS1^-bound internal aldimine (that is, an NFS1-bound PLP via Schiff base formation) to external (that is, non-protein-bound) Cys-aldimine and Cys-ketimine followed by generation of Ala-ketimine and concomitant Cys sulfur release for persulfide formation on Cys381^NFS1^ (Extended Data Fig. 7a)^19,40–42^. We used ultraviolet-visible (UV/Vis) spectroscopy to follow the generation of the external Cys-ketimine intermediate (absorption maximum at 340 nm) from the internal aldimine (absorption maximum at 416 nm) after addition of l-Cys or d-Cys^43^. The reactions were performed with (NIA)_2_ as well as with (NIA)_2_ plus bound FXN ((NIAX)_2_) or FXN-ISCU2 ((NIAUX)_2_), because the latter two proteins enhance NFS1 reactivity^43,44^. Notably, both Cys enantiomers elicited similar spectral changes with nearly identical time courses for all three complexes, while the presence of FXN (and ISCU2) accelerated the rates dramatically (Fig. 4b,c and Extended Data Fig. 7b,c). The absorption increase at 340 nm demonstrated that d-Cys was even more efficient than l-Cys in generating the external Cys-ketimine intermediate from the internal aldimine (decrease at 416 nm) for all three (NIA)_2_ complexes.

To explore whether l-Cys-ketimine or d-Cys-ketimine formation is influenced by the subsequent persulfide generation on Cys381^NFS1^ (Extended Data Fig. 7a), we used the NFS1-p.Cys381Ser variant that cannot undergo this conversion. In this case, the spectral changes and time courses were similar for both l-Cys and d-Cys (Supplementary Fig. 6). These results clearly indicated that it is mainly the Cys-ketimine and not Ala-ketimine (both absorb around 340 nm) intermediate that is visualized under these conditions. Further, the rate of conversion for all three complexes was slow (similar to (NIA)_2_ with l-Cys) and was not stimulated by FXN (and ISCU2) addition showing that replacing the active-site Cys381^NFS1^ for Ser had a profound influence on the rate and FXN-mediated stimulation of Cys-ketimine formation^45^. Importantly, the similarity between l-Cys and d-Cys in all these spectroscopic studies clearly indicated that the inability of NFS1 to form [2Fe–2S] clusters from d-Cys must occur at a step after the uncompromised Cys-ketimine formation, likely during sulfur mobilization and transfer to Cys381^NFS1^.

We therefore directly investigated the ability of the two Cys enantiomers to support Cys381^NFS1^ persulfidation using a Cys-alkylation-based gel-shift assay^39,44^ (Supplementary Fig. 7). Human NFS1 contains seven Cys residues, and persulfidation in this assay is visualized by the gel-mobility shift of the all-Cys-alkylated NFS1 (NFS1 + 7 species) to the mono-persulfidated species (NFS1 + 6). Addition of l-Cys to (NIA)_2_ was highly efficient in causing this mass loss, while d-Cys was totally ineffective, clearly demonstrating that d-Cys cannot support NFS1 persulfidation (Fig. 4d). Interestingly, in keeping with our enzymatic [2Fe–2S] cluster reconstitution data (Fig. 4a), a twofold excess of d-Cys over l-Cys had little effect on l-Cys-mediated persulfidation (Fig. 4d). In summary, the NFS1 Cys-alkylation experiment clearly identified the sulfur release from cysteine and subsequent Cys381^NFS1^ persulfide formation as the step that was blocked by d-Cys (Extended Data Fig. 7d).

### Structural explanation for failed d-Cys sulfur transfer

**Fig. 5:**
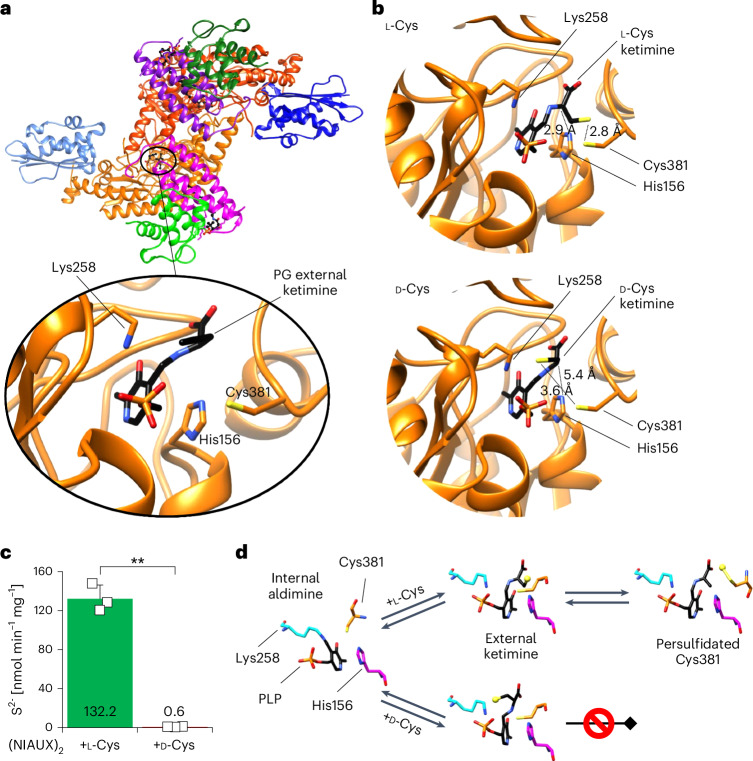
Structural orientation of d-Cys-ketimine precludes sulfur transfer. **a**, Crystal structure of (NIAU)_2_ after incubation with l-propargylglycine (PG; PDB 8TVT). The blow-up shows the NFS1 active centre harbouring an external PG-ketimine. This cofactor-substrate entity is no longer covalently bound to Lys258^NFS1^, and mimics the Cys-ketimine intermediate one step before actual persulfidation (Extended Data Fig. 7a). **b**, Models of bound l-Cys-ketimine (top) and d-Cys-ketimine (bottom) based on the crystal structure solved after addition of PG and validated using two bacterial SufS structures (PDB 7XEJ and PDB 7XEP). For l-Cys-ketimine both catalytic His156^NFS1^ and sulfur-accepting Cys381^NFS1^ are in close proximity to the l-Cys sulfur, while for d-Cys-ketimine these distances are much larger and the d-Cys sulfur is too far away for efficient persulfidation. For inspection of the model in 3D, a Chimera X file is available in Supplementary File 1. **c**, The (NIAUX)_2_ complex is not able to produce free sulfide from d-Cys using the DTT-dependent desulfurase activity assay. The rate of sulfide production was determined in *n* = *3* biological replicates, presented as the mean ± s.d., and compared by a paired two-tailed Student’s *t*-test, **P = 0.0039. **d**, A simplified mechanistic scheme for the reactions that l-Cys and d-Cys undergo with the NFS1-bound PLP (Extended Data Fig. 7a). When l-Cys or d-Cys are added to the enzyme, harbouring in its ground state the Lys258^NFS1^-bound PLP as an internal aldimine (Schiff base), an external (that is, non-enzyme-bound) Cys-ketimine is formed. Proton abstraction by His156^NFS1^ facilitates persulfidation of Cys381^NFS1^ for l-Cys but not d-Cys. Source data

**Extended Data Fig. 8:**
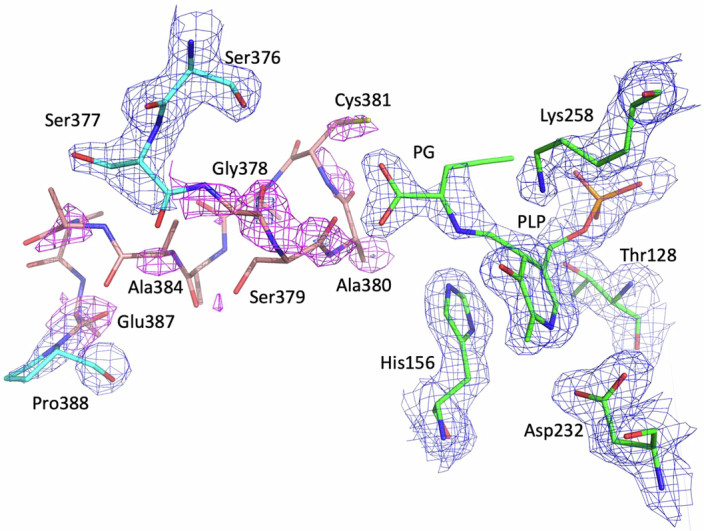
Electron density maps around pyridoxal-phosphate (PLP) in the L-propargylglycine (PG)-containing (NIAU)_2_ crystal structure. The 2Fo-DFc (blue; contoured at 0.5 sigma) and Fo-DFc (orange; 2.5 sigma) difference maps were calculated after 5 refinement macrocycles in Phenix of the model from which the PG-PLP, the sidechain of Lys258, and residues 377-387 were excluded (PDB ID: 8TVT). While the density for the loop is weak, it was possible to build backbones of residues Gly378-Cys381 and Ala384-Glu387. Most of the sidechains in this region were not visible and were not modeled. The Fo-DFc map clearly shows that Lys258 is not covalently bound to PLP, thus defining PG-PLP as an external aldimine. There appears to be density at low level to allow placing the sidechain of Cys381 in one possible conformation, where it points toward the PG sidechain.

To elucidate the precise molecular mechanism for the inability of d-Cys to support persulfidation within NFS1, we solved the crystal structure of (NIA)_2_ in complex with ISCU2 and the Cys-analogue l-propargylglycine (Protein Data Bank (PDB) 8TVT; Fig. 5a and Supplementary Table 6). As expected, the overall shape of this (NIAU)_2_ complex was nearly identical to the known X-ray structure without ligand addition (PDB 6W1D)^39^. However, in the NFS1 active site, we observed an external l-propargylglycine-ketimine (Fig. 5a and Extended Data Fig. 8), which is an analogue of the external Cys-ketimine intermediate that can be formed by both l-Cys and d-Cys (Fig. 4a–c and Supplementary Fig. 6). This l-propargylglycine-ketimine was structurally similar to a l-propargylglycine bound to the class II cysteine desulfurase SufS from *Bacillus subtilis*^46,47^. Therefore, we used our structure to model either an l-Cys-ketimine or a d-Cys-ketimine into the active centre of NFS1 (Fig. 5b). In creating these models, we superimposed the N-Cα-C(O) backbone of l-Cys or d-Cys on that of l-propargylglycine. In the case of l-Cys-ketimine, the Cys sulfur was located in close proximity to both the strictly conserved proton-abstracting His156^NFS1^ (2.9 Å)^46^ and the persulfide-accepting Cys381^NFS1^ (2.8 Å)^41^. In clear contrast, modelling of the d-Cys-ketimine revealed an orientation that was similar to a recently solved structure of *B. subtilis* SufS with bound d-Cys (PDB 7XEJ). In our NFS1 model, the d-Cys-ketimine sulfur was positioned in the almost opposite direction compared with l-Cys-ketimine, pointing towards Lys258^NFS1^ (Fig. 5b). This is best seen in a 3D rendition of the relative orientations of l-Cys and d-Cys-ketimines (Supplementary File 1). As a consequence, the sulfur was located far from both His156^NFS1^ and Cys381^NFS1^ (3.6 Å and 5.4 Å, respectively), thus making sulfur transfer to Cys381^NFS1^ both catalytically and sterically unfavourable (Extended Data Fig. 7d)^41^. We conclude that the altered location of the d-Cys sulfur is incompatible with sulfur mobilization and persulfidation.

To further confirm this view, we measured the release of free sulfide from the l-Cys or d-Cys-treated (NIAUX)_2_ complex in the presence of the artificial reductant dithiothreitol (DTT)^48^. Consistent with our structural data, only l-Cys but not d-Cys could support this reaction (Fig. 5c). In summary, NFS1 readily and efficiently forms the Cys-ketimine intermediate with both l-Cys and d-Cys but steric hindrance in the case of d-Cys prevents subsequent sulfur transfer to the acceptor Cys381^NFS1^ (Fig. 5d and Extended Data Fig. 7a,d). This unique property of NFS1 to initially react with d-Cys explains how the enzyme is selectively and even reversibly (Supplementary Fig. 6c) inhibited by d-Cys. In contrast, cystathionine β-synthase, a key enzyme in the transsulfuration pathway, which converts homocysteine to cystathionine using l-Cys as a co-substrate^49^, remained completely unreactive towards d-Cys (Supplementary Fig. 8). Similarly, glutathione synthase has been reported to be unable to utilize d-Cys as a substrate^13^. These results underscore how d-Cys functions as a highly selective inhibitor of NFS1.

### d-Cys effects are cell density dependent

**Extended Data Fig. 9:**
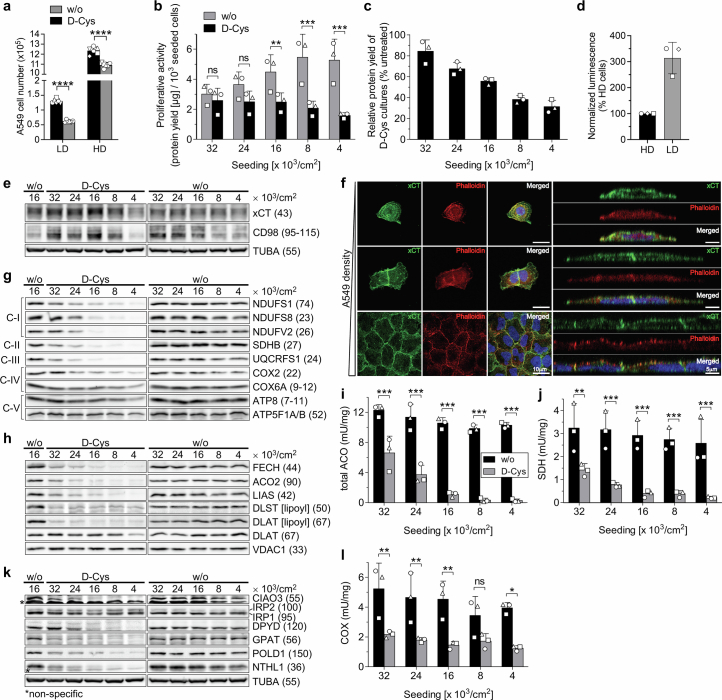
D-Cys induces Fe-S protein defects particularly at low cell densities. **a**, A549 cells were seeded at low density (LD, 5×10^3^ cells/cm^2^) or high density (HD, 60×10^3^ cells/cm^2^), cultured in the absence (w/o) or presence of 500 μM D-Cys for LD cultures and 1 mM for HD cultures. After three days cells were harvested and counted; experiments were done in technical triplicates and performed twice; 2-way ANOVA followed by Sidak’s multiple comparisons test; **** P < 0.0001. **b**, **c**, Indicated numbers of A549 cells were seeded into 25 cm^2^ flasks, cultured in the absence (w/o) or presence of 500 μM D-Cys for a total of three days and harvested. Protein yield of each culture at harvest (**b**), and relative protein yield of D-Cys-treated cultures in comparison to untreated cultures (**c**) are presented. **d**, A549 cells were cultured at LD or HD conditions as indicated in (**a**). After 15 h, the cellular D-Cys content was measured by the D-Cys luciferase assay, and expressed relative to the HD value. **e**, Protein levels of xCT and CD98 in cells from (**b**) were determined by immunoblotting. α-tubulin (TUBA) served as loading control. **f**, Immunofluorescence confocal analysis of xCT and phalloidin in A549 cells cultured at LD (top) to HD (bottom) conditions (left panels). Note the colocalization of xCT and phalloidin at HD. Z sections taken at the horizontal middle position of the respective XY view (left) are shown in right panels. Images are representative of n = 2 biological replicates. **g**, **h**, Cell samples from (**b**) were analysed by immunoblotting against the indicated mitochondrial proteins or lipoyl cofactor. VDAC1 served as loading control. C-I to C-V, OXPHOS complexes I to V. **i**, **j**, Total cell lysates of cells from (**c**) were analysed for the specific enzyme activities of total cellular aconitase (ACO) and succinate dehydrogenase (SDH, respiratory complex II). **k**, Cell samples were analysed by immunoblotting against the indicated cytosolic-nuclear CIA and Fe-S proteins. TUBA served as loading control. **l**, Crude membrane preparations of cells from (**b**) were analysed for the specific enzyme activity of cytochrome c oxidase (COX, respiratory complex IV). Representative blots and images are shown (**e**–**h**, **k**). Numbers in parentheses indicate observed molecular masses. Data obtained from n = 3 biological replicates are presented as mean ± SD, with individual symbols indicating matching samples (**b**–**d**, **i**, **j**, **l**); Comparisons were performed by 2-way repeated measures ANOVA and Bonferroni posttests (**b**, **c**, **i**, **j**, **l**); * P < 0.05; ** P < 0.01; *** P < 0.001; **** P < 0.0001; ns, not significant. Source data

d-Cys toxicity was strongly dependent on cell density, with growth inhibition being maximal at low densities (4–8 × 10^3^ cells per cm^2^) but weak at higher densities or confluency (Extended Data Fig. 9a–c). Intracellular d-Cys levels were threefold increased in subconfluent compared with confluent cultures (Extended Data Fig. 9d). No correlation between intracellular d-Cys levels and the total protein levels of xCT or CD98 was observed (Extended Data Fig. 9e). Instead, confocal immunofluorescence revealed xCT accumulation at A549 cell membrane contacts, particularly at high densities (Extended Data Fig. 9f). Because xCT is post-translationally phosphorylated by the mTORC2 kinase^50^, the efficiency of d-Cys transport by xCT may not be controlled solely by xCT protein abundance, but probably also involves other regulatory mechanisms. Consistently, at low cell densities, d-Cys addition strongly impaired the stability and activity of mitochondrial and cytosolic/nuclear Fe–S cluster-dependent proteins, while weaker effects were detected for high-density cultures (Extended Data Fig. 9g–l). Reference proteins lacking Fe–S clusters remained unaffected (Supplementary Fig. 9). These findings demonstrate that cell density and/or proliferation rates critically modulate xCT‑mediated d-Cys uptake and Fe–S protein deficiencies. These results are consistent with previous data showing that high cell density renders cells resistant to system xc^−^ inhibition^51,52^.

### d-Cys has therapeutic potential

**Fig. 6:**
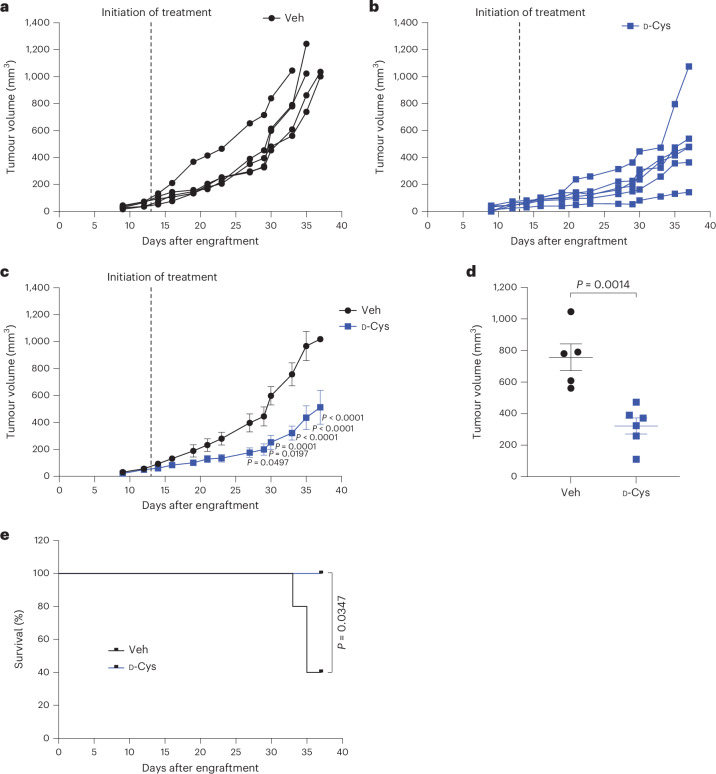
D-Cys impairs mammary tumour growth in the mouse. **a**–**c**, MDA-MB-231 cells were implanted orthotopically into the mammary gland of immunodeficient mice. When tumours reached between 50 mm^3^ and 100 mm^3^, mice were randomized and either did not receive d-Cys (Veh, *n* = 5 animals; **a**) or were administered d-Cys (*n* = 6 animals; **b**) as explained in Methods, and the tumour growth was monitored three times per week. The experiment was stopped at day 37 after tumour engraftment, when tumours had reached 1,000 mm^3^ in a majority of mice. The average tumour growth (mean ± s.d.) in vehicle-treated or d-Cys-treated mice at each timepoint is summarized in **c**. Data were analysed by two-way ANOVA followed by Sidak’s multiple-comparisons test. **d**, Average tumour volume (mean ± s.d.) of the mice from (**a**–**c**) at day 33 after tumour engraftment. Data were analysed by an unpaired two-tailed Student’s *t*-test. **e**, Survival curve of vehicle-treated or d-Cys-treated mice from **a**–**c** until the end of the experiment (day 37). Data were analysed by log-rank (Mantel–Cox) test. Source data

**Extended Data Fig. 10:**
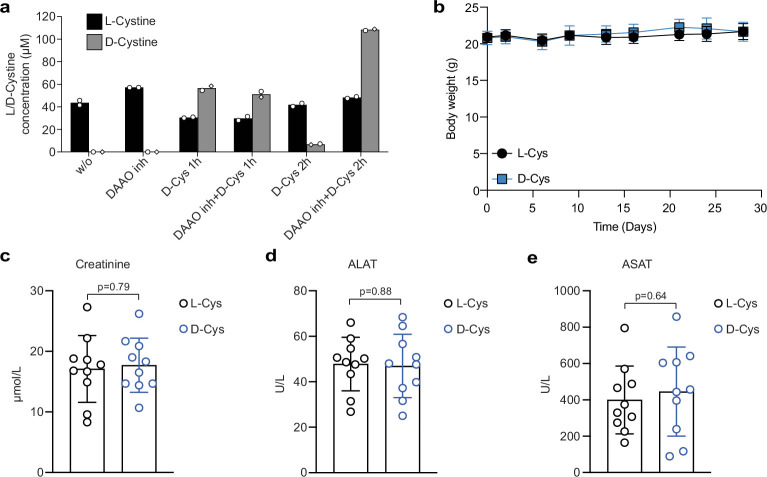
D-Cys clearance by D-amino acid oxidase and its lack of adverse effects. **a**, Immunodeficient mice were administered 200 μL of D-Cys (15 mg/mL in PBS) intraperitoneally, vehicle only (Veh), or the D-amino acid oxidase (DAAO) inhibitor 6-hydroxy-2-(naphthalen-1-ylmethyl)-1,2,4-triazine -3,5(2H,4H)-dione (ref. ^69^) (DAAO inh, 30 mg/kg, orally). A fourth group received both D-Cys and the DAAO inhibitor. Blood samples were collected from the tail at 1 and 2 h post-administration for the analysis of L-cystine and D-cystine levels via chiral chromatography (n = 2 each). **b**–**e**, Immunodeficient mice were fed a chow diet supplemented with either L-cystine or D-cystine for 28 days. Additionally, daily intraperitoneal (IP) and subcutaneous (SC) injections of D-cystine or vehicle were administered as detailed in Methods. Body weight was monitored throughout the experimental protocol (**b**). Twenty-eight days after starting the diet, mice were euthanized, and samples were collected for the measurement of creatinine (**c**), alanine aminotransferase (ALAT; **d**), and aspartate aminotransferase (ASAT; **e**). Data obtained from individual animals are presented as mean ± SD for control and D-Cys-treated groups (n = 10 each). Statistical analyses were performed using unpaired, two-tailed Student’s t-tests. ref. ^69^. Source data

We tested the effect of d-Cys on tumour growth in vivo using MDA-MB-231 breast cancer cells implanted orthotopically in immunodeficient mice. Maintaining a plasma concentration of d-cystine above the IC_50_ was challenging due to rapid clearance of d-aa enantiomers by DAAO^53^ (Extended Data Fig. 10a). To optimize plasma levels, mice were fed a chow diet containing d-cystine instead of l-cystine and given two daily d-Cys injections (intraperitoneal (i.p.) and subcutaneous (s.c.)) 8 h apart, 5 days a week. This regimen was well tolerated, with no weight loss (Extended Data Fig. 10b) and no obvious deviations of kidney (creatinine; Extended Data Fig. 10c) or liver (alanine aminotransferase and aspartate aminotransferase; Extended Data Fig. 10d,e) parameters. d-Cys administration significantly reduced tumour growth (Fig. 6). All untreated mice developed tumours reaching 1,000 mm^3^ by days 32–37 (Fig. 6a,c). In contrast, only in one of six d-Cys-treated mice tumours reached this size by day 37 (Fig. 6b,c), with the average tumour size being reduced by more than twofold (Fig. 6d). Thus, d-Cys alone reduced tumour growth and improved animal survival (Fig. 6e). Given the sensitivity of d-Cys toxicity to oxygen and cell density (Extended Data Fig. 5e and Extended Data Fig. 9), the strongest toxic effects likely may occur at the periphery of the tumour, where oxygen is available and contacts between cancer cells are diminished.

## Conclusion

In conclusion, our findings establish d-Cys as a potent NFS1 inhibitor with therapeutic potential for cancer treatment. d-Cys disrupts cellular Fe–S protein functions, and consequently impairs, for example, mitochondrial respiration, nuclear DNA metabolism and cancer cell proliferation. Notably, the cytotoxic effects of d-Cys are particularly pronounced under normoxic conditions. This observation aligns with prior research demonstrating that defects of mitochondrial Fe–S protein biogenesis are exacerbated by oxygen^26,54^. Further, NFS1 knockdown inhibits cancer cell proliferation in oxygen-rich environments and prevents circulating cancer cells exposed to high oxygen levels from forming metastases in a murine mammary tumour model^25^. The selective sensitivity of certain cancer cells to d-Cys is tightly connected to their elevated xCT expression^55,56^ allowing increased d-Cys uptake. This may be combined with a deficiency in DAAO^57^, an enzyme that metabolizes d-Cys into the cytoprotective molecule H_2_S^7^. Although this protective pathway is present in neurons and kidney cells^51^, its absence in many cancers, due to a lack of DAAO expression^57^, could render these cells particularly vulnerable to d-Cys. These characteristics make d-Cys a promising therapeutic candidate for targeting oxygen-rich tumours and circulating metastatic cells. Additionally, d-Cys holds potential as a powerful research tool for rapidly inhibiting Fe–S protein biogenesis.

## Methods

### Cell culture

Human lung adenocarcinoma A549 cells (American Type Culture Collection (ATCC) CCL-185, gift from the lab of P. Hofman, IRCAN, Nice, France), human breast adenocarcinoma MDA-MB-231 cells (gift from the lab of D. Picard, University of Geneva, Switzerland), human bronchial epithelial BEAS-2B cells (ATCC CRL-9609, gift from the lab of P. Hofman), human tumorigenic lung BZR cells obtained by transfer of the v-Ha-ras oncogene into BEAS-2B cells (ATCC CRL-9483, gift from the lab of P. Hofman), human lung epidermoid carcinoma Calu1 cells, human melanoma A375 cells (ATCC CRL-1619, gift from the lab of P. Hofman), human cervix cancer HeLa cells, human colon cancer HCT116 and DLD1 cells, lung adenocarcinoma LuCa62 cells derived from a patient (gift from V. Serre-Beinier, CMU, Geneva, Switzerland), human embryonic kidney HEK 293 cells and human retinal pigment epithelial RPE-1 cells were grown in DMEM high-glucose media supplemented with 10% FBS, 2 mM l-glutamine, 100 U ml^−1^ penicillin and 100 µg ml^−1^ streptomycin at 37 °C and 5% CO_2_. Mesothelioma JL1, ZL34 and H2052/484 cell lines (gift from V. Serre-Beinier) were grown in RPMI 1640 with GlutaMAX media (Life Technologies, 61870-010) supplemented with 10% FBS, 100 U ml^−1^ penicillin and 100 µg ml^−1^ streptomycin (Corning, 30-002-CI) in an incubator at 37 °C and 5% CO_2_. A549 Bax/Bak double knockout cells were a generous gift from A. Sfeir (Sloan Kettering Institute).

### Cell culture treatments

For the IC_50_ determination of d-Cys or d-cystine, and for most proliferation analyses, cells were seeded in six-well plates at a density of 5 × 10^3^ cells per cm^2^, unless specifically mentioned, and incubated overnight at 37 °C. Cells were then treated with d-Cys (Carl ROTH, 7874.1) or d-cystine (Sigma-Aldrich, 285463) at the indicated concentrations. When not specified, d-Cys was used at 500 µM. For cell counting assays, cells were harvested by trypsination at the indicated times and counted in 0.4% trypan blue solution using a Neubauer counting chamber. For metabolic and proteomic analyses, cells were lysed 72 h after the treatments. For biochemical analyses, A549 and BEAS-2B cells were seeded into tissue culture flasks at densities between 32 × 10^3^ and 4 × 10^3^ cells per cm^2^, grown overnight, supplemented with 500 µM d-Cys for the following 2 days, subjected to a medium exchange including d-Cys replenishment, and harvested the next day. For erastin treatment, 24 h after seeding the cells were treated with 1 µM compound (Sigma-Aldrich, E7781) in the presence or absence of 500 µM d-Cys. Seventy-two hours later, cells were harvested and counted as described above.

Transient Cys-free cultivation of A549 cells was performed in Cys-free/Met-free DMEM (Thermo Fisher, 21013-024), with addition of 200 µM methionine and standard supplements. When indicated, cells were grown in the presence of 375 µM TCEP. Tissue culture media including l-Cys, d-Cys or erastin supplements were freshly prepared and exchanged daily. The autoxidation of d-Cys and TCEP under normoxic tissue culture atmosphere was monitored in 50 mM Tris/HCl buffer (pH 7.4) by Ellman’s reagent DTNB (5,5′-dithiobis-(2-nitrobenzoic acid).

For hypoxia experiments, the culture medium was pre-equilibrated for 4 days in a sealed hypoxic chamber maintained at 1% O_2_ and 5% CO_2_ before initiating the experiment. A549 cells (1.5 × 10^5^ cells per well) were seeded overnight in six-well plates, after which the medium was replaced with pre-equilibrated hypoxic growth medium. The cells were cultured with or without 500 μM d-Cys. The plates were then placed in a sealed, humidified hypoxic chamber containing 1% O_2_ and 5% CO_2_ and incubated at 37 °C for 72 h before performing cell counting or crystal violet staining.

### Colony formation assays

A total of 1,000 cells per dish were seeded in 6-cm dishes and incubated overnight at 37 °C. Twenty-four hours later, cells were treated with 100 µM d-Cys for 2 weeks. Cells were then washed with PBS, fixed in 4% paraformaldehyde (PFA) solution and stained with 0.5% crystal violet solution (Sigma-Aldrich, C0775) in 30% ethanol for 20 min. The stained cells were then washed with H_2_O and left to dry overnight.

### Spheroid formation assays

A total of 1,000 cells per well were seeded in culture media containing 100 µM to 500 µM d-Cys in 96-well plates coated with 1.5% agarose. Spheroid volume was quantified by light microscopy twice per week for 17 days.

### Generation of CRISPR–Cas9 knockout cells

A549 cells were transduced with lentivirus containing lentiCas9-Blast plasmid (gift from F. Zhang, Addgene plasmid no. 52962). After 24 h, cells were selected with 5 µg ml^−1^ blasticidin for 7 days. After selection, cells were seeded at one cell per well in 96-well plates for cell cloning. Following single-cell colony formation, clones were screened for Flag expression and one Flag-positive clone was selected. Then, Cas9-expressing cells were infected with lentivirus containing a single guide RNA (sgRNA) of interest in lentiGuide-Puro vector (Addgene, plasmid no. 52963). For each gene of interest, two sgRNAs were used. The sgRNA target sequences used were as follows:

*SLC3A2* (CD98)-1: GACCTTACTCCCAACTACCG;

*SLC3A2* (CD98)-2: TGAGTGGCAAAATATCACCA;

*SLC7A11* (xCT)-1: AAGGGCGTGCTCCAGAACAC;

*SLC7A11* (xCT)-2: GAAGAGATTCAAGTATTACG;

*NFE2L2*-1: CACATCCAGTCAGAAACCAG;

*NFE2L2*-2: CATACCGTCTAAATCAACAG;

*NCOA4*-1: AGATTGGCTAGTGACTCCCC;

*NCOA4*-2: GAGGTGTAGTGATGCACGGA.

After 24 h, cells were selected with 5 µg ml^−1^ puromycin for 7 days and expression of the gene of interest was verified by western blotting.

### Pooled gRNA depletion screen

Cas9-expressing A549 cells were infected with the human CRISPR Brunello lentiviral pooled library (Addgene, 73178-LV) at a multiplicity of infection of 0.3 such that every gRNA is represented in 500 cells. After 48 h, infected cells were selected with puromycin for at least 6 days. After selection, 40,000,000 cells were seeded per condition to maintain the expression of the whole library. The following day, cells were treated with H_2_O (control condition) or with 500 µM of l-Cys or d-Cys. Cells in control and d-Cys conditions were passaged every 3 days and maintained at a minimum of 40,000,000 cells. Cells in the d-Cys condition were refreshed with media containing 500 µM of d-Cys every 3 days until the emergence of resistant clones. At least 40,000,000 cells were collected for genomic DNA extraction at day 0 after selection and at the end of the treatment (day 11 for control and l-Cys conditions, and day 24 for d-Cys condition). Genomic DNA was extracted from cell pellets. gRNA inserts were amplified via PCR using primers harbouring Illumina TruSeq adapters with i5 and i7 barcodes, and the resulting libraries were sequenced by next-generation sequencing on an Illumina HiSeq 4000.

### Overexpression of SLC3A2 and SLC7A11 in BEAS-2B cells

To generate the *SLC3A2* plasmid the sequence AgeI-SLC3A2-XhoI-3xGly linker-FLAG-STOP-EcoRI was inserted between the AgeI and EcoRI sites of TRIPz replacing the TurboRFP. To make the *SLC7A11* plasmid the sequence AgeI-SLC7A11-SalI was inserted between the AgeI and XhoI sites of the *SLC3A2* construct giving AgeI-SLC7A11-SalI/XhoI-3xGly linker-FLAG-STOP-EcoRI. The final sequence of both plasmids was verified.

BEAS-2B cells were infected with lentivirus containing pTRIPz-EGP (gift from the lab of D. Picard), pTRIPz-SLC3A2-Flag or pTRIPz-SLC7A11-Flag. After 24 h, cells were selected with 5 µg ml^−1^ puromycin for 7 days. After selection, cells were seeded for d-Cys treatment at 100 µM in combination with 100 ng ml^−1^ doxycycline. After 72 h, cells were harvested and counted.

### Transient overexpression of *SLC3A2* and *SLC7A11* in HeLa cells

Open reading frames of C-terminally FLAG-tagged *SLC3A2* and *SLCA11* were subcloned into pEGFP-derived mammalian expression vectors (TaKaRa) by substitution of the EGFP open reading frame using standard cloning techniques. Transfection of HeLa cells by electroporation^22^ was performed in 265 µl of buffer using 6 µg of each CD98-encoding and xCT-encoding plasmid or 12 µg of a reference vector encoding a PEST-sequence destabilized EGFP. After overnight tissue culture, cells were supplemented with 500 µM d-Cys. Two days later, medium including d-Cys was replaced and cells were harvested the following day. Cells were thus exposed to d-Cys for a total of 3 days.

### Generation of A549 FUCCI cells

A549 cells were transduced with lentivirus containing pBOB-EF1-FastFUCCI-Puro plasmid (gift from the lab of D. Picard, Addgene plasmid no. 86849). After 24 h, cells were selected with 5 µg ml^−1^ puromycin for 7 days. Surviving cells were seeded on coverslips in six-well plates, for d-Cys treatment as described above, then fixed for 20 min at room temperature in 4% PFA solution. The coverslips were washed and mounted on slides for imaging. The proportion of cells in G1 phase (mKO-hCdt1, red probe) and in S/G2, G2 or M phases (mAG-hGeminin, green probe) was quantified.

### Immunofluorescence procedures

#### BrdU staining

Cells were seeded in six-well plates containing coverslips and treated with increasing concentrations of d-Cys as described above. After 72 h, cells were incubated with 10 μM BrdU for 8 h in cell culture medium at 37 °C. Then, cells were washed, fixed for 20 min at room temperature in 4% PFA solution and permeabilized in PBS containing 0.15% Triton X-100 for 10 min at room temperature, before addition of 2 M HCl to denature DNA. Cells were then incubated in PBS containing 0.15% Triton X-100 and 5% normal goat serum for 30 min at room temperature and incubated in a solution containing anti-BrdU antibody (Sigma-Aldrich, 11170376001) diluted at 1:200 and 5% normal goat serum at 4 °C overnight. The last step of immunostaining was performed according to standard immunocytochemistry protocols.

#### DNA damage staining

Cells were seeded in six-well plates containing coverslips and treated with d-Cys as described above. After 72 h, cells were washed, fixed for 20 min at room temperature in 4% PFA solution and permeabilized in PBS containing 0.15% Triton X-100 for 10 min at room temperature. Cells were incubated in PBS containing 0.15% Triton X-100 and 1% BSA for 30 min at room temperature and incubated in anti-53BP1 and anti-γ-H2AX antibodies diluted at 1:50 and 1:200, respectively, in a solution containing 1% BSA, at 4 °C overnight. The last step of immunostaining was performed according to standard immunocytochemistry protocols.

#### Immunofluorescence detection of xCT and CD98

For immunofluorescence, cells were cultured on glass coverslips in 24-well plates for 2 days seeded at a low density of 12,500 cells per well and at a high density of 2 × 10^5^ cells per well. Cells were washed two times with cold PBS, fixed in 1% PFA for 12 min, followed by rinsing with and incubating with methanol (MeOH) at −20 °C for 5 min, and 2× washes in PBS. Cells were permeabilized with 0.2% of Triton X-100 in PBS (5 min at room temperature) and saturated for 20 min with 2% of BSA in PBS. Incubation with primary antibodies (xCT; 1:200 dilution) was carried out for 2 h at room temperature, followed by washing 3× with PBS; incubation with anti-rabbit secondary antibody (Supplementary Table 7) and rhodamine-phalloidin (Thermo Fisher Scientific, R415; 1:400 dilution) was carried out for 1 h at room temperature, followed by 3× washes with PBS. Coverslips were mounted with Fluoromount-G. Slides were imaged on a Zeiss LSM 800 confocal microscope using a Plan-Apochromat ×63/1.40 oil objective (1,024 × 1,024 pixels). Maximum intensity projections of *z*-stack images (typically 3–6 confocal planes over 1.0–1.5 µm, step size = 0.3 µm) were obtained. *z*-sections were collected in 0.27-μm steps over 6 µm. Images were extracted from .czi files using ImageJ, adjusted and cropped using Adobe Photoshop, and assembled in Microsoft PowerPoint.

### Electron microscopy

Cells grown on Permanox slides were washed in 100 mM phosphate buffer (KH_2_PO_4_/Na_2_HPO_4_; pH 7.4) and fixed for 20 min at room temperature in sodium cacodylate buffer supplemented with 2.5% glutaraldehyde. Cells were post-fixed for 20 min at room temperature in 2% osmium tetroxide, and pre-stained in 2% of uranyl acetate for 10 min at room temperature. After several washes in phosphate buffer, cells were dehydrated sequentially in 50%, 70%, 90% and 100% ethanol (for 10 min for each procedure). The samples were then infiltrated sequentially in 1:1 (vol/vol) ethanol:Epon resin (EMS), 1:3 ethanol:Epon resin for 30 min for each procedure, 100% Epon resin for 3 h, and finally covered with BEEM embedding capsules (EMS) filled with 100% Epon resin. Polymerization was initiated by raising the temperature to 60 °C and keeping them at this temperature during 48 h. Ultrathin sections were isolated on copper grids and stained for 10 min in 2% uranyl acetate and for 5 min in Reynold’s lead citrate, and examined at 120 kV using a Tecnai G2 transmission electron microscope.

### Western blotting

Protein content was quantified using the Pierce BCA protein assay kit (Thermo Fisher Scientific, 23227). Proteins of cell lysates were separated by SDS–PAGE or tricine–PAGE and immunoblotted according to standard techniques. Antibodies used are listed in Supplementary Table 7.

### Mitochondrial reactive oxygen species production

A total of 50,000 cells per well were seeded in six-well plates and treated with d-Cys. After 72 h, cells were stained with 5 µM mitoSOX (Molecular probes, M36008) diluted in Dulbecco′s phosphate buffered saline with MgCl_2_ and CaCl_2_ (DPBS Mg/Ca, Sigma-Aldrich, D8662) for 15 min at 37 °C. Cells were then harvested and resuspended in 300 µl PBS for flow cytometry analysis. A Gallios flow cytometer (Beckman Coulter) was used for the analysis.

### Lipid peroxide production

In total, 50,000 cells per well were seeded in six-well plates and treated with d-Cys. After 72 h, cells were harvested and resuspended in 1 ml DPBS Mg^2+/^Ca^2+^ containing 5 µM BODIPY 581/591 C11 (Invitrogen, D3861) for 20 min at 37 °C. Then, cells were washed and resuspended in 300 µl PBS for flow cytometry analysis. A Gallios flow cytometer (Beckman Coulter) was used for the analysis.

### Oxygen consumption measurements

A total of 40,000 cells per well previously cultured in the absence or presence of 500 µM d-Cys for 48 h were seeded in Agilent Seahorse XF24 cell culture microplates (Bucher Biotec AG, 100777-004) and cultured for an additional 15 h in the presence or absence of d-Cys. Then cells were incubated for 1 h in DMEM media (Sigma-Aldrich, D5030) supplemented with 25 mM D-glucose, 2 mM glutamine (PAN BIOTECH, P04-80100) and 25 mM Hepes (Thermo Fischer Scientific, 15630080) at pH 7.4 without CO_2_ before plate reading. Oxygen consumption rate was measured under basal conditions or following the addition of oligomycin (2 μM), the uncoupler FCCP (1 μM) and the electron transport inhibitors rotenone (1 μM) and antimycin A (1 μM) using a Seahorse XFe24 analyser.

### Biochemical analyses of tissue culture samples

Separation of cellular constituents into a crude mitochondria-containing organellar and a cytosolic fraction by digitonin-based plasma membrane permeabilization was performed as described^20^. Enzyme activities were analysed in multiwell plates based on established assays^27,58^.

### Metabolomics

#### Sample preparation

Cells were extracted by the addition of MeOH:H_2_O (4:1; 1 ml). This solution containing scraped lysed cells was further homogenized in the Cryolys Precellys 24-sample homogenizer (2 × 20 s at 10,000 rpm, Bertin Technologies) with ceramic beads. Homogenized extracts were centrifuged for 15 min at 4,000*g* at 4 °C, and the resulting supernatant was collected and evaporated to dryness in a vacuum concentrator (LabConco). Dried sample extracts were resuspended in MeOH:H_2_O (4:1, vol/vol) before liquid chromatography–tandem mass spectrometry (LC–MS/MS) analysis according to the total protein content.

#### LC–MS/MS

Cell lysates were analysed by hydrophilic interaction liquid chromatography coupled to tandem mass spectrometry (HILIC-MS/MS) in both positive and negative ionization modes using a 6495 triple-quadrupole system (QqQ) interfaced with a 1290 ultra-high-performance liquid chromatography (UHPLC) system (Agilent Technologies) In positive mode, the chromatographic separation was carried out in an Acquity BEH Amide, 1.7 μm, 100 mm × 2.1 mm internal diameter (i.d.) column (Waters). The mobile phase was composed of A = 20 mM ammonium formate and 0.1% formic acid in water and B = 0.1% formic acid in acetonitrile (ACN). The linear gradient elution from 95% B (0–1.5 min) down to 45% B (1.5–17 min) was applied, and these conditions were held for 2 min, followed by 5 min of column re-equilibration at the initial gradient conditions. The flow rate was 400 μl min^−1^, the column temperature was 25 °C, and the sample injection volume was 2 µl. In negative mode, a SeQuant ZIC-pHILIC (100 mm, 2.1 mm i.d. and 5-μm particle size, Merck) column was used. The mobile phase was composed of A = 20 mM ammonium acetate and 20 mM NH_4_OH in water at pH 9.7 and B = 100% ACN. The linear gradient elution from 90% B (0–1.5 min) to 50% B (8–11 min) down to 45% B (12–15 min) was applied, followed by a 9-min post-run routine for column re-equilibration. The flow rate was 300 μl min^−1^, the column temperature was 30 °C, and the sample injection volume was 2 µl. For both analyses, the ESI source conditions were set as follows: dry gas temperature, 290 °C; nebulizer 35 psi, flow rate 14 l min^−1^; sheath gas temperature 350 °C, flow 12 l min^−1^; nozzle voltage, 0 V; capillary voltage, ±2,000 V. Data were acquired in dynamic multiple reaction monitoring (DMRM) mode with a total cycle time of 600 ms. Pooled quality-control (QC) samples (representative of the entire sample set) were analysed periodically throughout the overall analytical run, to assess the quality of the data, correct the signal intensity drift and remove the peaks with poor reproducibility (coefficient of variation > 30%). In addition, a series of diluted QC samples were prepared by dilution with MeOH: 100% QC, 50% QC, 25% QC, 12.5% QC and 6.25% QC and analysed at the beginning and at the end of the sample batch. This QC dilution series served as a linearity filter to remove the features that do not respond linearly or where correlation with the dilution factor is <0.

#### Data processing and statistical analysis

Raw LC–MS/MS data were processed using the Agilent Quantitative analysis software (version B.07.00, MassHunter Agilent technologies). Relative quantification of metabolites was based on Extracted Ion Chromatogram areas for the monitored MRM transitions. Data quality assessment was done in R (http://cran.r-project.org/). Signal intensity drift correction was done within the LOWESS/Spline algorithm followed by filtering of ‘not-well behaving’ peaks (coefficient of variation (QC peaks) > 30% and *R*^2^ (QC dilution curve < 0.75). A *t*-test (on log_10_-transformed data) was used to test the significance of metabolite changes in different conditions with an arbitrary level of significance, *P* value = 0.05 (and adjusted *P* value corrected for multiple testing with the Benjamini–Hochberg method).

### Thiol quantification

#### Sample preparation

Cell culture was extracted by the addition of 1 ml of an extraction solution of MeOH:water:borate buffer 50 mM:iodoacetamide 1 M (80/9.6/8.8/1.6). This solution containing scraped lysed cells was further homogenized in the Cryolys Precellys 24-sample homogenizer (2 × 20 s at 10,000 rpm, Bertin Technologies) with ceramic beads. Homogenized extracts were centrifuged for 15 min at 4,000*g* at 4 °C, and the resulting supernatant was collected and evaporated to dryness in a vacuum concentrator (LabConco). Dried sample extracts were resuspended in water before LC–MS/MS analysis.

#### LC–MS/MS

Cell lysates were analysed by LC–MS/MS in both positive ionization mode using a 6495 triple-quadrupole system (QqQ) interfaced with a 1290 UHPLC system (Agilent Technologies). Chromatographic separation was carried out in an Acquity UPLC HSS T3 (2.1 mm × 100 mm × 1.8 µm) column (Waters). The mobile phase was composed of A = 0.1% formic acid in water and B = 0.1% formic acid in ACN. An isocratic step of 2.5 min at 100% A was applied, followed by a gradient elution down to 35% A and these conditions were held for 1.6 min, followed by 4 min of column re-equilibration at the initial gradient conditions. The flow rate was 400 μl min^−1^, the column temperature was 25 °C, and the sample injection volume was 2 µl. ESI source conditions were set as follows: dry gas temperature, 290 °C; nebulizer 35 psi, flow 14 l min^−1^; sheath gas temperature 350 °C, flow 12 l min^−1^; nozzle voltage, 0 V; capillary voltage, 4000 V. DMRM was used as acquisition mode with a total cycle time of 600 ms. Optimized collision energies for each metabolite were applied.

Raw LC–MS/MS data acquired in DMRM mode was processed using the Agilent Quantitative analysis software (version B.07.00, MassHunter Agilent technologies). Quantification of thiols was based on Extracted Ion Chromatogram areas for the monitored SRM transitions. For absolute quantification, calibration curves and the stable isotope-labelled internal standards were used to determine the response factor. Linearity of the standard curves was evaluated for each metabolite using 12 calibration points; in addition, peak area integration was manually curated and corrected when necessary.

#### Protein quantification

The protein pellets were evaporated and lysed in 20 mM Tris-HCl (pH 7.5), 4 M guanidine hydrochloride, 150 mM NaCl, 1 mM Na_2_EDTA, 1 mM EGTA, 1% Triton, 2.5 mM sodium pyrophosphate, 1 mM beta-glycerophosphate, 1 mM Na_3_VO_4_ and 1 µg ml^−1^ leupeptin using the Cryolys Precellys 24-sample homogenizer (2 × 20 s at 10,000 rpm, Bertin Technologies) with ceramic beads. BCA Protein Assay Kit (Thermo Scientific) was used to measure (A_562 nm_) total protein concentration (Hidex).

### d-Cys quantification using 4-fluoro-7-nitrobenzofurazan derivatization

#### In the plasma

Biological samples were processed following the protocol described by Ferré et al.^18^. Briefly, dl-Cys (3,3-d_2_, 98%) was used as internal standard, and samples were sequentially submitted to protein precipitation, Cys reduction and derivatization. The resulting extracts were analysed using UHPLC coupled to triple-quadrupole MS. Details on sample preparation and analysis are provided in ref. ^18^. The cysteine levels obtained through this procedure were derived from initial reduction of plasma cystine into cysteine. As we can consider that most cysteine in the plasma is oxidized into cystine, to get the plasma cystine levels, we divided the cysteine values obtained by UHPLC/MS by two. These values are reported in Extended Data Fig. 10a.

#### In cells

Biological samples were processed as described in ref. ^18^. In addition, in one experiment, d-Cys was measured using the d-Cys luciferase assay^6^.

### Whole-cell proteomic analysis

#### Sample preparation

Samples were digested with trypsin using the Filter-Aided Sample preparation^59^ protocol with minor modifications. Proteins were resuspended in 200 µl of 8 M urea and 100 mM Tris-HCl and deposited on top of Microcon-30K devices. Samples were centrifuged at 9,391*g* at 20 °C for 30 min. All subsequent centrifugation steps were performed using the same conditions. An additional 200 µl of 8 M urea and 100 mM Tris-HCl was added, and devices were centrifuged again. Reduction was performed by adding 100 µl of 10 mM TCEP in 8 M urea and 100 mM Tris-HCl on top of filters followed by a 60-min incubation at 37 °C, protected from light and with gentle shaking. Reduction solution was removed by centrifugation, and filters were washed with 200 µl of 8 M urea and 100 mM Tris-HCl. After removal of washing solution by centrifugation, alkylation was performed by adding 100 µl of 40 mM chloroacetamide in 8 M urea 100 mM Tris-HCl and incubating the filters at 37 °C for 45 min with gentle shaking and protection from light. The alkylation solution was removed by centrifugation and another washing/centrifugation step with 200 µl of 8 M urea and 100 mM Tris-HCl was performed. This last urea buffer washing step was repeated twice followed by three additional washing steps with 100 µl of 5 mM Tris-HCl. Proteolytic digestion was performed overnight at 37 °C by adding on top of filters 100 µl of Endoproteinase Lys-C and Trypsin Gold in an enzyme/protein ratio of 1:50 (wt/wt). Resulting peptides were recovered by centrifugation. The devices were then rinsed with 50 µl of 4% trifluoroacetic acid and centrifuged. This step was repeated three times, and peptides were finally desalted on C18 StageTips^60^.

For TMT labelling, dried peptides were first reconstituted in 10 μl 100 mM HEPES pH 8 and 4 μl of TMT solution (25 µg μl^−1^ in pure ACN) was then added. TMT labelling was performed at room temperature for 1.5 h, and reactions were quenched with hydroxylamine to a final concentration of 0.4% (vol/vol) for 15 min. TMT-labelled samples were then pooled at a 1:1 ratio across all samples. A single-shot control LC–MS run was performed to ensure similar peptide mixing across each TMT channel to avoid the need for further excessive normalization. The combined samples were then desalted using a 100 mg SEP-PAK C18 cartridge (Waters) and vacuum centrifuged. Pooled samples were fractionated into 12 fractions using an Agilent OFF-Gel 3100 system following the manufacturer’s instructions. Resulting fractions were dried by vacuum centrifugation and again desalted on C18 StageTips.

#### Data acquisition

For LC–MS/MS analysis, resuspended peptides were separated by reversed-phase chromatography on a Dionex Ultimate 3000 RSLC nano UPLC system connected in-line with an Orbitrap Q-exactive HF (Thermo Fisher Scientific). A capillary precolumn (Acclaim Pepmap C18, 3 μm 100 Å, 2 cm × 75-μm i.d.) was used for sample trapping and cleaning. A capillary column (75-μm i.d.; in-house packed using ReproSil-Pur C18-AQ 1.9-μm silica beads; Dr. Maisch; length, 50 cm) was then used for analytical separations at 250 nl min^−1^ over a gradient. Acquisitions were performed through Top 15 Data-Dependent acquisition. The first mass spectrometry scans were acquired at a resolution of 120,000 (at 200 *m/z*) and the most intense parent ions were selected and fragmented by high energy collision dissociation with a normalized collision energy of 32% using an isolation window of 0.7 *m/z*. Fragmented ion scans were acquired at a resolution of 30,000 (at 200 *m/z*) using a fixed maximum injection time of 100 ms, and selected ions were then excluded for the following 40 s.

#### Data analysis

Protein identification and isobaric quantification were performed using MaxQuant (1.6.10.43)^61^. The Human Uniprot reference proteome database (last modified on 5 Jul 2019; 74,468 sequences) was used for this search. Carbamidomethylation was set as a fixed modification, whereas oxidation (M), phosphorylation (S, T, Y) and acetylation (protein N-term) were considered as variable modifications. A maximum of two missed cleavages were allowed for this search. A minimum of two peptides were allowed for protein identification, and the FDR cut-off was set to 0.01 for both peptides and proteins.

#### Data processing

Resulting text files were processed through in-house written R scripts (version 3.6.3; https://www.R-project.org/). A first normalization step was applied according to the sample loading normalization^62^. Assuming that the total protein abundances were equal across the TMT channels, the reporter ion intensities of all spectra were summed, and each channel was scaled according to this sum, so that the sum of reporter ion signals per channel equals the average of the signals across samples. A trimmed M-mean normalization step was also applied using the package EdgeR^63^ (version 3.26.8). Assuming that samples contain a majority of non-differentially expressed proteins, this second step calculates normalization factors according to these presumed unchanged protein abundances. Proteins with high or low abundances and proteins with larger or smaller fold-change values are not considered.

### Purification of proteins for biochemical assays

For purification of the proteins used for biochemical assays, *E. coli* cells expressing the appropriate proteins^37,39^ were thawed at room temperature, resuspended in 35 mM Tris-HCl pH 8 containing 300 mM NaCl, 5% (wt/vol) glycerol and 10 mM imidazole (buffer P) and lysed by sonication (SONOPULS mini20, BANDELIN electronic GmbH & Co. KG). Cell debris was removed by centrifugation at 40,000*g* for 45 min. For all proteins except for FDX2 the supernatant containing the soluble protein was subjected to Ni-NTA affinity chromatography (Prepacked His-Trap 5-ml FF crude column; GE Healthcare) and subsequent size exclusion chromatography (SEC; 16/60 Superdex 75 or 200; GE Healthcare) on an Äkta Purifier 10 system (GE Healthcare). For SEC and protein storage, the buffer was adjusted to 35 mM Tris-HCl pH 8, 150 mM NaCl and 5% (wt/vol) glycerol (buffer S). Elution from Ni-NTA matrix was achieved by applying a linear gradient from 0.01 to 1 M imidazole. Proteins typically eluted between 120 mM and 250 mM of imidazole.

Exceptions from the standard procedure: NFS1–ISD11–ACP1 complex proteins were co-expressed (*NFS1* and *ISD11* genes were inserted into pET-Duet MCSI and MCSII, respectively, and *ACP1* into pRSF-Duet MCSI^39^, and co-purified using the His6-tag fused to ISD11 in buffer P additionally containing 5 mM PLP. Subsequently, the complex was purified to homogeneity by SEC in buffer S, and elution fractions contained a bright yellow protein. ISCU2 and variants were treated with EDTA, KCN and DTT before SEC to remove potentially bound metal ions and/or polysulfides. FXN was treated with self-made recombinant TEV protease, β-mercaptoethanol and DTT before SEC to cleave the N-terminal His6-tag (which renders the protein fully inactive) and to remove potential metal ion contaminations. FDX2 was purified using anion exchange chromatography and subsequent SEC. For anion exchange chromatography, 35 mM Tris-HCl pH 8 containing 20 mM NaCl (buffer A) was used. Elution was performed applying a linear gradient increasing the NaCl concentration from 0.02 M to 1.0 M. FDX2 typically eluted at a NaCl concentration of 150–300 mM. SEC was performed as outlined above and yielded a dark brown protein solution.

### Spectroscopic methods

UV/Vis absorption spectroscopy was performed on a Jasco V550 (JASCO Deutschland). (NIA)_2_ or the (NIA)_2_-p.Cys381Ser variant was diluted to 25 µM or 50 µM in 250 µl of buffer, respectively, and either buffer (for measuring the initial ground state of the enzyme) or l-Cys or d-Cys was added to the sample to reach a final concentration of 5 mM. Stock solutions of Cys (100 mM) were buffered in 1 M Tris-HCl pH 8.0. To generate the (NIAX)_2_ or (NIAUX)_2_ complex, (NIA)_2_ was supplemented with two equivalents of either FXN or both FXN and ISCU2, respectively. Full spectra of the reaction mixtures were recorded approximately every 2 min at room temperature. Plots were created with Excel by plotting the absorption value of the indicated wavelength for each timepoint.

### Persulfide transfer assay

Persulfide transfer experiments (Supplementary Fig. 5) were done anaerobically at room temperature using degassed buffer T1 (50 mM Tris-HCl pH 7.4, 150 mM NaCl, 5% wt/vol glycerol) in a final reaction volume of 20 µl. Reactions containing 20 µM (NIA)_2_ were initiated by addition of the indicated amounts of (pre-mixed) l-Cys and d-Cys (200 µM final Cys concentration). As a negative control, no Cys was added. Persulfidation was terminated after 10 s using 2.72 mM EZ-Link maleimide-PEG_11_-biotin (MPB, Thermo Scientific), that is, eight equivalents over total thiol concentration. After 20 s, SDS was added to a final concentration of 1% (wt/vol), and after another 10 min of incubation, samples were removed from the anaerobic chamber for analysis by SDS–PAGE (8% acrylamide gel). To this end, reaction aliquots containing 2 µg (NIA)_2_ were incubated in sample buffer with 50 mM TCEP for 15 min at room temperature. Gels were stained using InstantBlue (Expedeon, ISB1L) according to the manufacturer’s instruction.

### Affinity measurements

Protein–Cys interactions were measured using microscale thermophoresis on a Monolith 1.15 (Nanotemper Technologies). Measurement settings were: LED power at 30% ((NIA)_2_ and (NIA)_2_-p.Cys381Ser), laser power at 75%, temperature fixed at 21 °C. Either (NIA)_2_ or (NIA)_2_ containing NFS1-p.Cys381Ser were labelled according to the manufacturer’s instructions (Monolith Protein Labeling Kit RED-NHS 2nd Generation, MO-L011, Nanotemper Technologies). Around 200 nM of labelled protein was titrated with a 1:1 dilution series of either l-Cys or d-Cys starting from 2 mM. Data were analysed using Origin 8 G (OriginLab Corporation).

### Enzymatic Fe–S cluster reconstitution on ISCU2 proteins

Enzymatic reconstitution by the core ISC complex was followed by circular dichroism spectroscopy^37,38^ in reconstitution buffer (35 mM Tris pH 8.0, 150 mM NaCl, 0.8 mM sodium ascorbate, 0.3 mM FeCl_2_, 0.5 mM NADPH, 0.2 mM MgCl_2_), and 5 mM glutathione and 5 µM mouse Grx1 were added to the sample. Protein concentrations used were: 150 µM ISCU2; 5 µM each of FXN, FDX2, (NIA)_2_; 1 µM FDXR. The reaction was started by the addition of 3 µl of l-Cys or d-Cys or a mixture of both. Final concentration of Cys was adjusted to 1 mM. In the case of 10× and 20× excess of d-Cys, the concentration of l-Cys was adjusted to 0.5 mM and d-Cys was supplied to a final concentration of 5 mM and 10 mM, respectively, while the volume of added Cys was kept constant at 3 µl (see above).

### NFS1 desulfurase activity assay

Cysteine desulfurase activity of purified (NIA)_2_ and (NIA)_2_ with NFS1-p.Cys381Ser in complex with FXN and ISCU2 was determined by the DTT-dependent sulfide generation assay^40,64^ with minor modifications. In this non-physiological assay, purified protein was incubated at 30 °C in 25 mM tricine, pH 8.0, 1 mM DTT and 1 mM l-Cys or d-Cys. After 20 min, the reaction was stopped by addition of 4 mM *N*,*N*-dimethyl-*p*-phenylenediamine sulfate (in 7.2 N HCl) and 3 mM FeCl_3_ (in 1.2 N HCl). Samples were incubated for another 20 min in the dark and the amount of methylene blue was determined spectrophotometrically at 670 nm.

### Structure of (NIA)_2_ in complex with ISCU2 and the Cys-analogue l-propargylglycine

#### Protein purification and l-propargylglycine incorporation

The (NIAU)_2_ complex was purified and concentrated to 17–20 mg ml^−1^ (ref. ^38^). l-propargylglycine (Sigma, 81838) was incorporated by incubation of purified (NIAU)_2_ complex with 1 mM l-propargylglycine on ice for 2 h.

#### Protein crystallization and structure solution

Prepared (NIAU)_2_ complex was crystallized similarly to a published protocol^38^. Diffraction data were collected at the CMCF sector of the Canadian Light Source using a Pilatus 6M detector. The initial structure was obtained by molecular replacement method, within the PHENIX software package^65^ using PDB 6W1D coordinates as a starting model. The structure was refined using PHENIX^65^ and manually rebuilt with COOT^66^.

### Cystathionine beta-synthase assay

Cystathionine beta-synthase (CBS) activity was measured as described in ref. ^67^. The assay solution contained Tris-HCl (50 mM, pH 8.0), human recombinant CBS (1 µg per well), PLP (5 µM final concentration), SAM (100 µM final concentration) and the H_2_S-specific fluorescent probe 7-azido-4-methylcoumarin (AzMC,10 µM final concentration) in a 96-black flat-well plate format. The plates were incubated at 25 °C for 10 min, followed by CBS activity triggered by adding the substrates homocysteine (1 mM) and l-Cys or d-Cys (at increasing concentrations). The increase in the AzMc fluorescence in each well was read at an excitation of 365 nm and an emission of 450 nm (over a 2-h time course at 37 °C).

### Animal experiments

Eleven-week-old female immunodeficient mice (Athymic Nude-Foxn1nu) were obtained from Envigo (France) and housed in a pathogen-free environment at TransCure bioService (Archamps, France), at 55% ± 10% humidity and 22 °C ± 2 °C with a 12–12-h light–dark cycle (7:00:19:00). Water and food were provided at libitum. The mice were surgically engrafted into the mammary fat pad with 5 × 10^6^ MDA-MB-231 tumour cells in 50% basement membrane matrix (Geltrex). When the average tumour volume reached approximately 50 mm^3^, mice were randomized and some were fed on a chow diet containing 6 g d-cystine and no l-cystine (Research Diets) and received an i.p. injection of 200 μl d-Cys (15 mg ml^−1^ in PBS, pH 7–7.4), between 8:00 and 9:00. Eight hours later, mice received a s.c. injection of 200 μl d-Cys (15 mg ml^−1^) or vehicle until euthanasia. Control mice were fed on the same diet as administered to the d-cystine-treated mice, except that l-cystine replaced d-cystine and that the control mice received i.p. and s.c. injection of PBS. All parenteral treatments were administered on Mondays to Fridays only. Mice were euthanized when the tumour volume reached 1,000 mm^3^. The experiment was stopped when a majority of the mice had to be euthanized according to ethical reasons in place at TransCure bioService, where all these experiments were performed. TransCure bioService is a Contract Research Organization and its authorization number for experiments on animals is DAP: 2022082413416895. Data collection and analysis were not performed blind to the conditions of the experiments. The technicians responsible for handling the mice were aware of the general nature of the treatments they administered but did not know their exact composition or intended effects.

Experiments to assess d-Cys plasma levels after i.p. or oral administration of d-Cys or d-cystine were performed at the Medical University of Geneva on 2-month-old female athymic nude-Foxn1nu mice (Envigo). Experiments were performed in accordance with the Institutional Animal Care and Use Committee of the University of Geneva and with permission of the Geneva cantonal authorities (authorization number GE14420).

The effects of d-Cys on body weight, creatinine and plasmatic levels of liver enzymes alanine aminotransferase and aspartate aminotransferase were assessed using adult nude mice treated for 28 days with d-Cys as described above. Experiments were performed at C3M, Inserm, Nice in accordance with the Animal Care and Use Committee (PEA804). Creatinine and liver enzymes were measured with a Cobas c 111 instrument.

### Statistics and reproducibility

Most data are presented as the mean ± s.d. Pairwise comparisons were performed by Student’s *t*-test, for multiple-comparisons ANOVA, and post hoc tests were applied as indicated. Dependent data were analysed using paired and repeated-measures methods. Data distribution was assumed to be normal, but this was not formally tested. For the in vivo experiments, comparisons of the tumour growth in control and d-Cys-treated mice were analysed by two-way ANOVA followed by Sidak’s multiple-comparisons test or unpaired two-tailed Student’s *t*-test. For animal survival analyses, Kaplan–Meier survival curves were analysed by log-rank (Mantel–Cox) test. One control mouse was found dead in its cage 2 days after treatment randomization. The tumour was poorly developed, and the cause of death was not further investigated. This animal was excluded from the final analysis.

### Reporting summary

Further information on research design is available in the Nature Portfolio Reporting Summary linked to this article.

## Supplementary information

Supplementary Information Supplementary Figs. 1–9, legends for Supplementary Tables 1–7 and Supplementary File 1, and supplementary references.

Reporting Summary

Supplementary File 1 Chimera X file (structural data).

Supplementary Table 1 Supplementary Table 1.

Supplementary Table 2 Supplementary Table 2.

Supplementary Table 3 Supplementary Table 3.

Supplementary Table 4 Supplementary Table 4.

Supplementary Table 5 Supplementary Table 5.

## Source data

Source Data Fig. 1 Statistical source data.

Source Data Fig. 2 DStatistical source data.

Source Data Fig. 3 Unprocessed western Blots.

Source Data Fig. 3 DStatistical source data.

Source Data Fig. 5 Statistical source data.

Source Data Fig. 6 DStatistical source data.

Source Data Extended Data Fig. 1 Statistical source data. Extended Data Fig. 1.

Source Data Extended Data Fig. 2 Unprocessed western blots.

Source Data Extended Data Fig. 2 Statistical source data.

Source Data Extended Data Fig. 3 Unprocessed western blots.

Source Data Extended Data Fig. 3 DStatistical source data.

Source Data Extended Data Fig. 4 Unprocessed western blots.

Source Data Extended Data Fig. 5 Original flow cytometry data.

Source Data Extended Data Fig. 5 Statistical source data.

Source Data Extended Data Fig. 6 Unprocessed western blots.

Source Data Extended Data Fig. 6 Statistical source data.

Source Data Extended Data Fig. 9 Unprocessed western blots.

Source Data Extended Data Fig. 9 Statistical source data.

Source Data Extended Data Fig. 10 Statistical source data.

## Data Availability

Original data and images and western blots have been deposited in Zenodo (https://zenodo.org/uploads/15569071)^68^ or are supplied as source data. The mass spectrometry proteomics data have been deposited in the ProteomeXchange Consortium via the PRIDE [1] partner repository under dataset identifier PXD063479. Structural data have been deposited at the PDB (8TVT). Any additional information required to reanalyse the data reported in this paper is available from the corresponding authors upon request. Source data are provided with this paper.
