# Supplementary material for: d-cysteine impairs tumour growth by inhibiting cysteine desulfurase NFS1
Source: Nat Metab. 2025 Aug 12;7(8):1646–62. doi: 10.1038/s42255-025-01339-1 (PMC12373508; doi:10.1038/s42255-025-01339-1)
Supplement: Supplementary file 1 — Supplementary Figs. 1–9, legends for Supplementary Tables 1–7 and Supplementary File 1, and supplementary references. [file 42255_2025_1339_MOESM1_ESM.pdf]

nature metabolism

Supplementary information https://doi.org/10.1038/s42255-025-01339-1

D-cysteine impairs tumour growth by inhibiting cysteine desulfurase NFS1

In the format provided by the authors and unedited

Supplementary information

Content:

Supplementary Figures 1 – 9

(Legends for) Supplementary Tables 1 – 7

Legend for Supplementary File 1

Supplementary References

Supplementary Figures

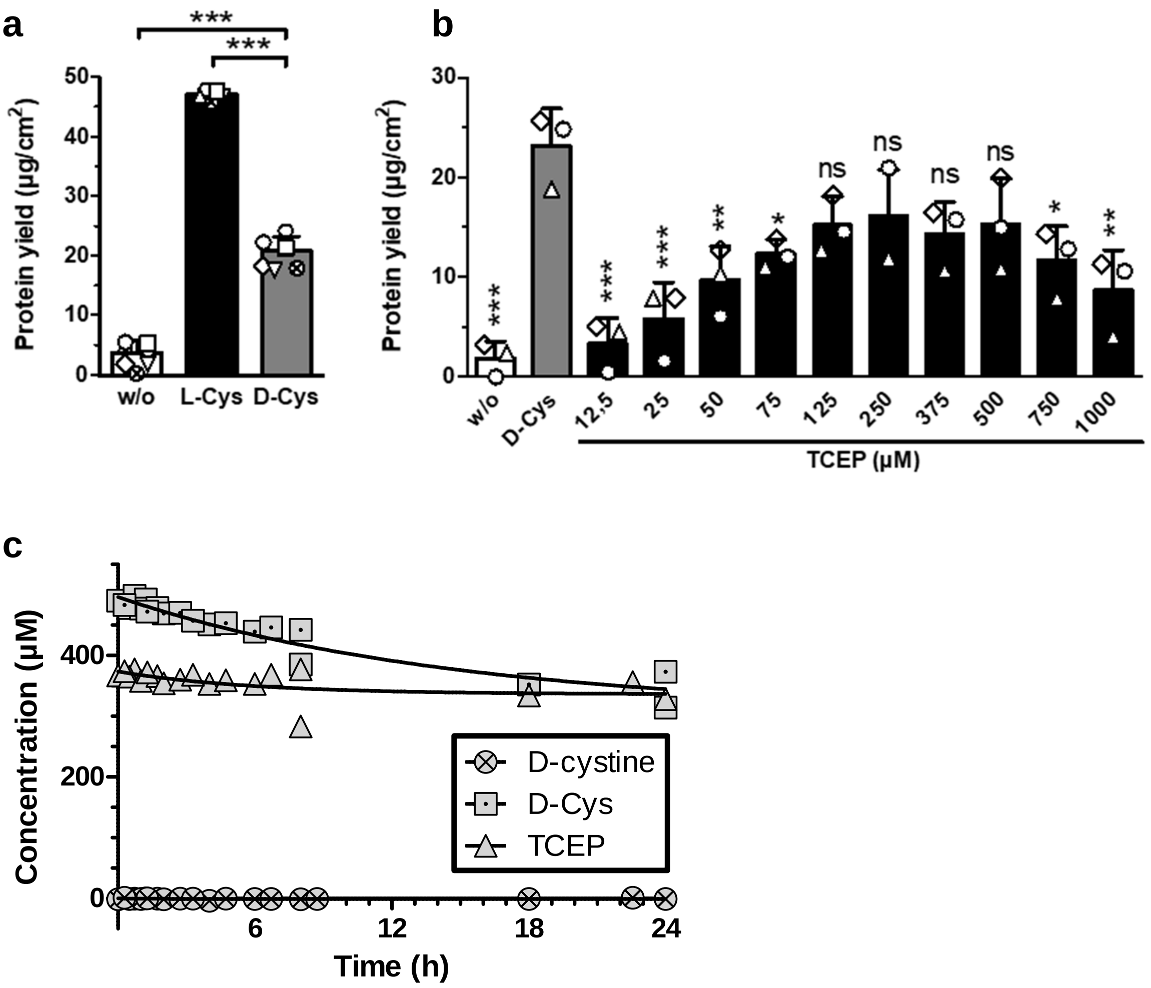

Supplementary Fig. 1. Reducing agents maintain cell viability of Cys-depleted cells. **a,** A549 cells were cultured in Cys-free but FCS-containing tissue culture medium for three days without further supplementation (w/o), or with supplementation of either 500 µM L-Cys or D-Cys. The tissue culture supernatant was exchanged daily to maintain constant levels of freshly prepared Cys. Eventually, cells were harvested and total protein yield was determined (n=7 for w/o and D-Cys; n=5 for L-Cys). **b,** A549 cells were cultured and analyzed as in (**a**) but instead of L-Cys in presence of various concentrations of the reductant TCEP (n=3). Comparisons in (**a**,**b**) were performed by 1-way ANOVA and Bonferroni posttests; symbols indicate matching samples of n biological replicates; data are presented as mean ± SD; *P < 0.05; **P < 0.01; ***P < 0.001; ns, not significant. **c,** Time course of the spontaneous oxidation of TCEP (starting concentration 375 µM) and D-Cys (starting concentration 500 µM) in normoxic tissue culture atmosphere. Analysis of D-cystine served as control. Note the slow oxidation rate of D-Cys and TCEP of 25% and 8% per 24 h, respectively.

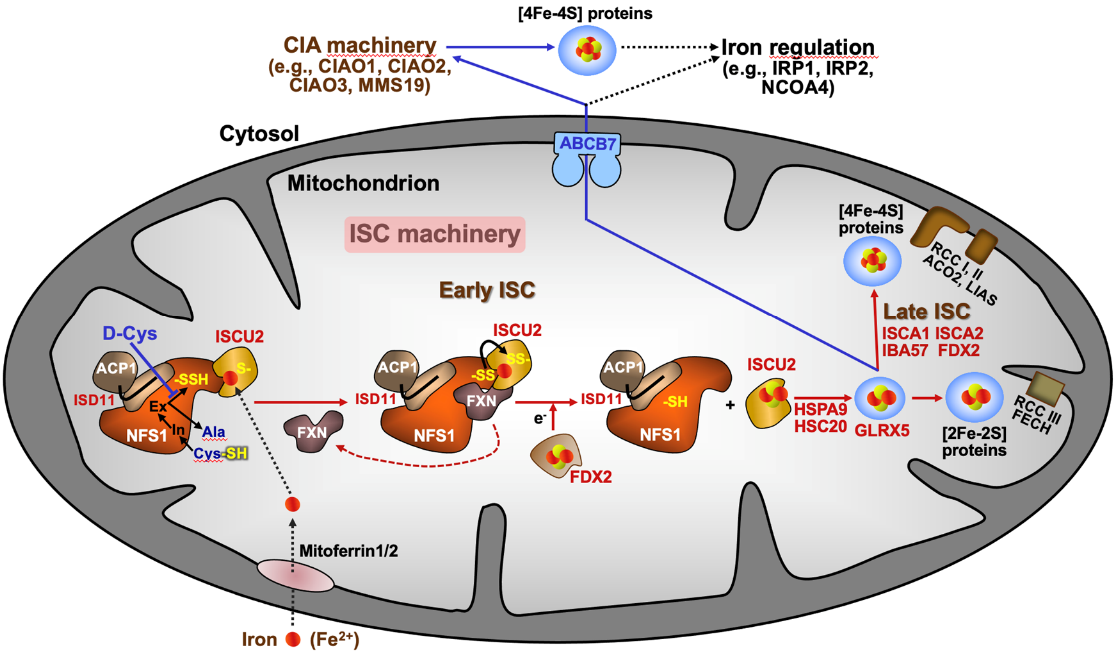

Supplementary Fig. 2. Simplified model of eukaryotic Fe/S protein biogenesis and the mode of D-Cys inhibition of NFS1. The biogenesis of cellular Fe/S proteins starts in mitochondria, where the early components of the ISC machinery assemble a [2Fe-2S] cluster de novo on the scaffold protein ISCU2 in several steps[1]. First, the cysteine desulfurase complex NFS1-ISD11-ACP1 converts the internal aldimine of pyridoxal phosphate (In) with free cysteine to an external Cys-aldimine/ketimine (Ex; see Fig. 5d and Extended Data Fig. 7a for mechanistic details). Cys-sulfur transfer from 'Ex' to an active-site cysteine of NFS1 generates a persulfide (-SSH) and alanine (Ala). As shown in this work, this step is specifically inhibited by D-Cys (Fig. 5d and Extended Data Fig. 7d). Second, the NFS1 persulfide is transferred to ISCU2 which binds iron imported into mitochondria by Mitoferrin1/2. Sulfur transfer is accelerated by frataxin (FXN) binding. Third, electron transfer from ferredoxin FDX2 leads to the reduction of the persulfide sulfur to sulfide and subsequent generation of a [2Fe-2S] cluster on ISCU2 by dimerization of two [1Fe-1S] intermediate-carrying ISC complexes (not shown). Fourth, the chaperone system HSPA9-HSC20B facilitates the transfer of the [2Fe-2S] cluster from ISCU2 to the glutaredoxin GLRX5, which then spontaneously assembles [2Fe-2S] target proteins (e.g., ferrochelatase, respiratory chain complex III). Fifth, the late ISC system (including ISCA1, ISCA2, IBA57, and FDX2) converts two [2Fe-2S] to a [4Fe-4S] cluster which then is inserted into target proteins (e.g., respiratory chain complexes I and II, aconitase, lipoyl synthase). The early ISC machinery together with the mitochondrial inner membrane ABC transporter ABCB7 is further required for assisting the cytosolic iron–sulfur protein assembly (CIA) machinery in the maturation of cytosolic and nuclear Fe/S proteins. Additionally, this latter pathway is involved in cellular iron regulation, e.g., by maturing the [4Fe-4S] cluster-binding iron regulatory protein 1 (IRP1; for details see Refs. [2,3].

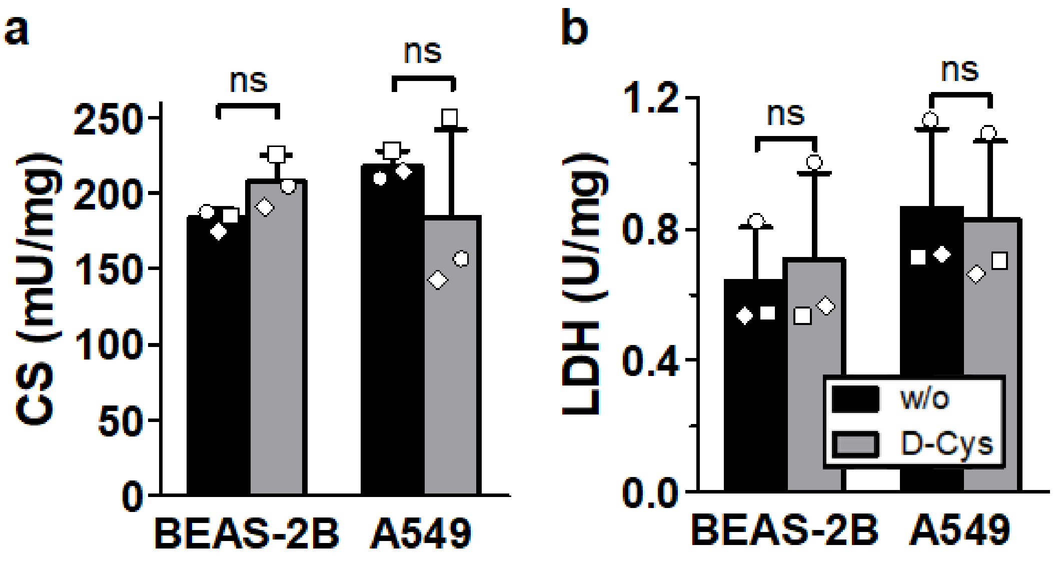

Supplementary Fig. 3. Control enzyme activities for D-Cys treated cells. Mitochondria-containing organellar (**a**) and cytosolic (**b**) fractions obtained by digitonin-based cell separation were analysed for the specific enzyme activities of mitochondrial citrate synthase (CS) and cytosolic lactate dehydrogenase (LDH), respectively (mean ± SD). Samples were taken from the experiment described in Fig. 3 and analysed by 2-way repeated measures ANOVA and Bonferroni posttests; symbols indicate matching samples of n=3 biological replicates. ns, not significant.

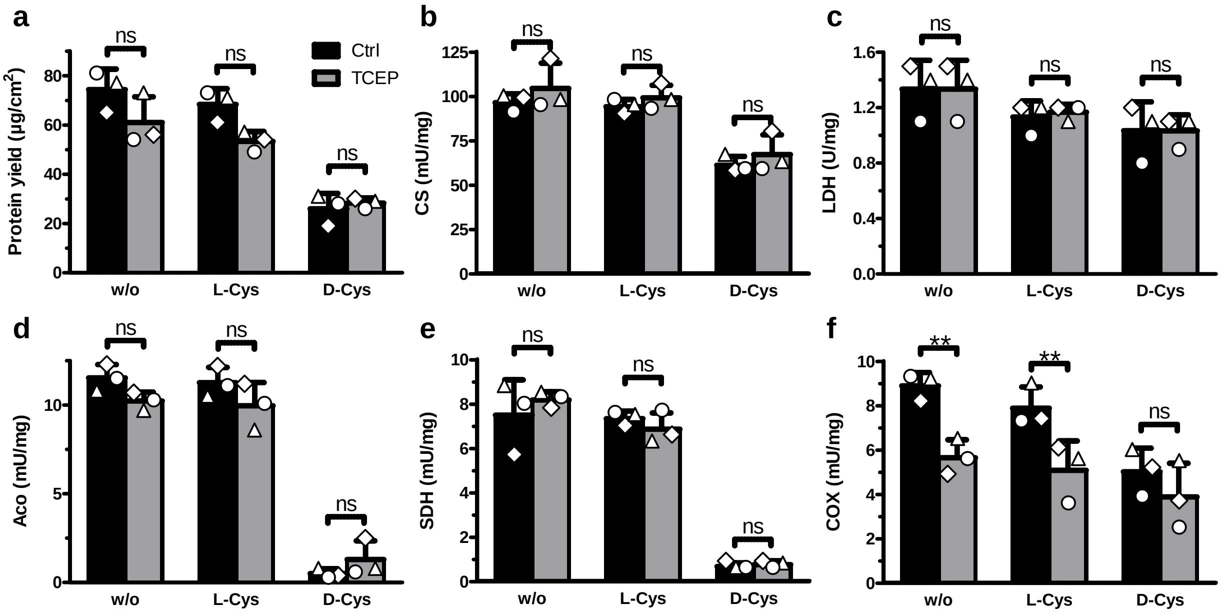

Supplementary Fig. 4. TCEP does not modify D-Cys-mediated toxicity. A549 cell cultures were maintained similar to Fig. 3, but in the absence (Ctrl) or presence of 375 µM TCEP, and were additionally supplemented with 500 µM L-Cys or D-Cys, or remained without Cys supplementation (w/o). Total protein yield after harvest was determined (**a**). Total cell lysates were analysed for the specific enzymes activities of the non-Fe-S reference proteins mitochondrial citrate synthase (CS; **b**) and cytosolic lactate dehydrogenase (LDH; **c**), as well as of the Fe-S-dependent protein aconitase (Aco; **d**), the Fe-S- and heme-dependent protein succinate dehydrogenase (SDH; **e**), and the heme dependent cytochrome *c* oxidase (COX; **f**). Data are presented as mean ± SD, and comparisons were performed by 2-way repeated measures ANOVA and Bonferroni posttests; symbols indicate matching samples of n=3 biological replicates. ***$P < 0.01$; ns, not significant.

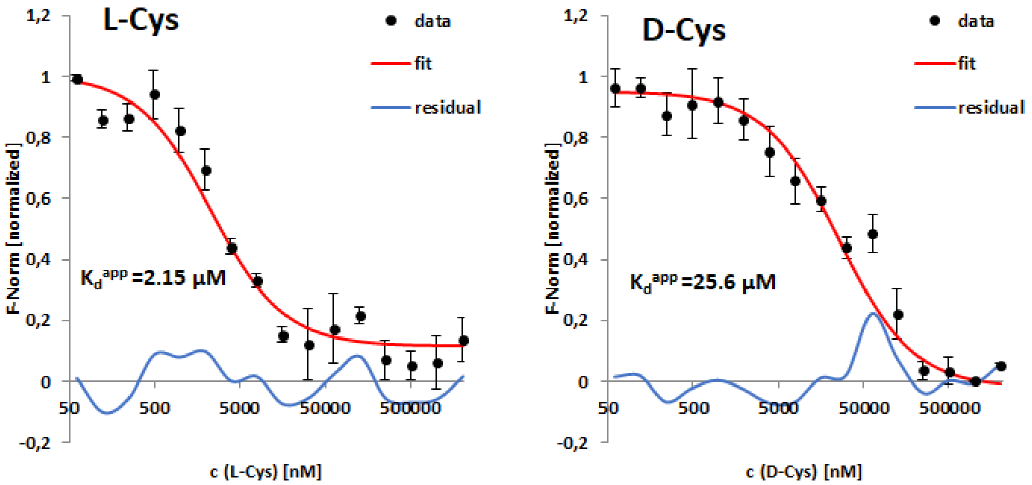

Supplementary Fig. 5. Original data for apparent affinity determinations of L-Cys or D-Cys for the NFS1-ISD11-ACP1 complex by Microscale Thermophoresis. The NFS1-C381S variant was used for these experiments to avoid turnover of L-Cys by $(NIA)_2$. Mean values ± SD of n=3 biological replicates are shown.

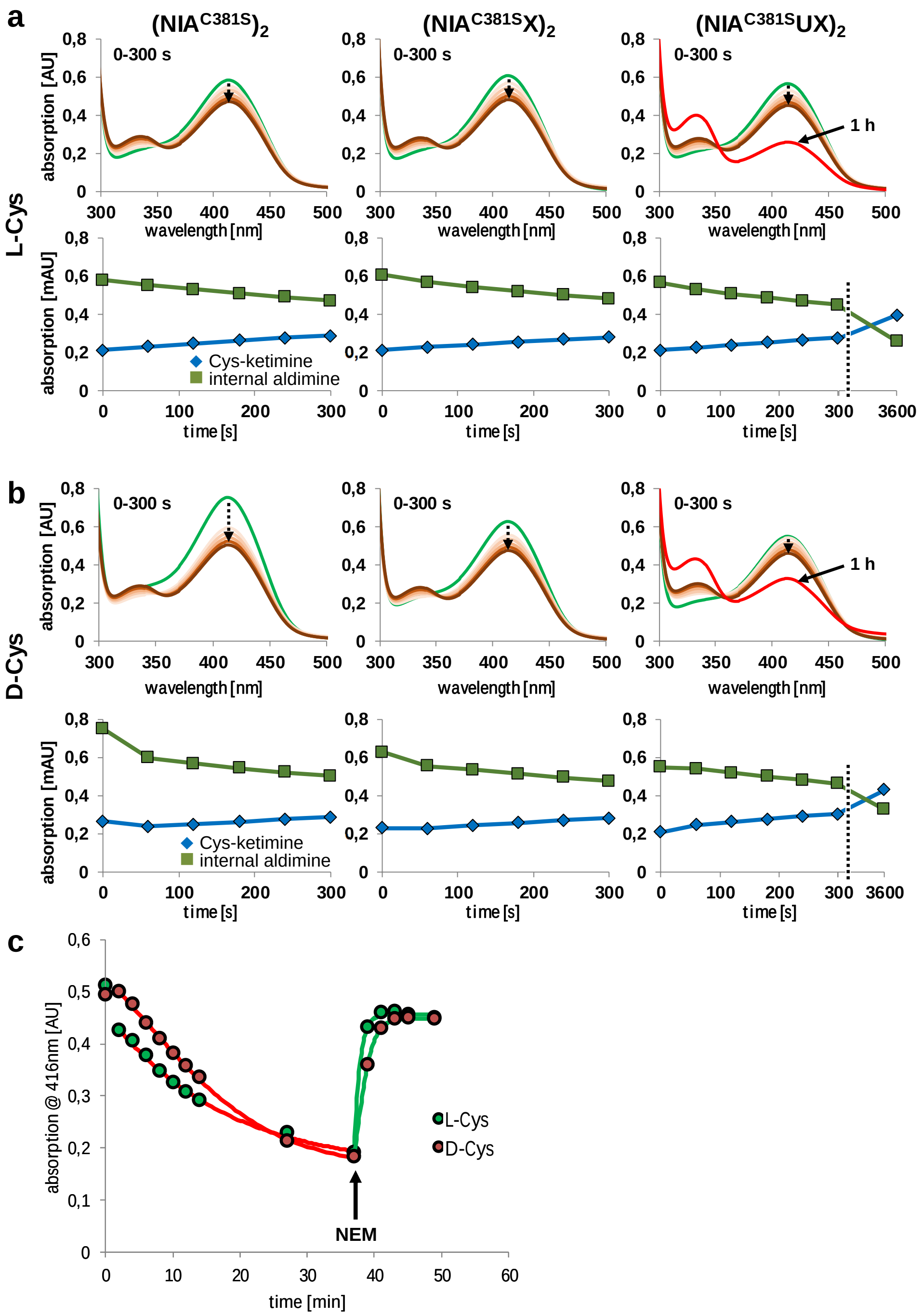

Supplementary Fig. 6. NFS1 can generate both the L- and D-Cys-ketimine independently of the active-site cysteine residue 381 and in reversible fashion. a,b, UV-Vis spectra (top panels) and time courses (bottom panels) for the generation of the L-Cys-ketimine (**a**) and D-Cys-ketimine (**b**) intermediates (absorption at 340 nm, blue) from the internal aldimine (416 nm, green) by the indicated $(NIA)_2$ complexes containing the NFS1-C381S variant unable to form a persulfide. **c**, The wild-type $(NIA)_2$ complex (25 µM) was incubated with 1 mM L- or D-Cys, and the loss of the internal aldimine was recorded at 416 nm. Addition of 5 mM thiol-reactive N-ethyl-maleimide (↑; NEM) was able to fully regenerate the internal aldimine indicating reversibility of Cys-ketimine formation.

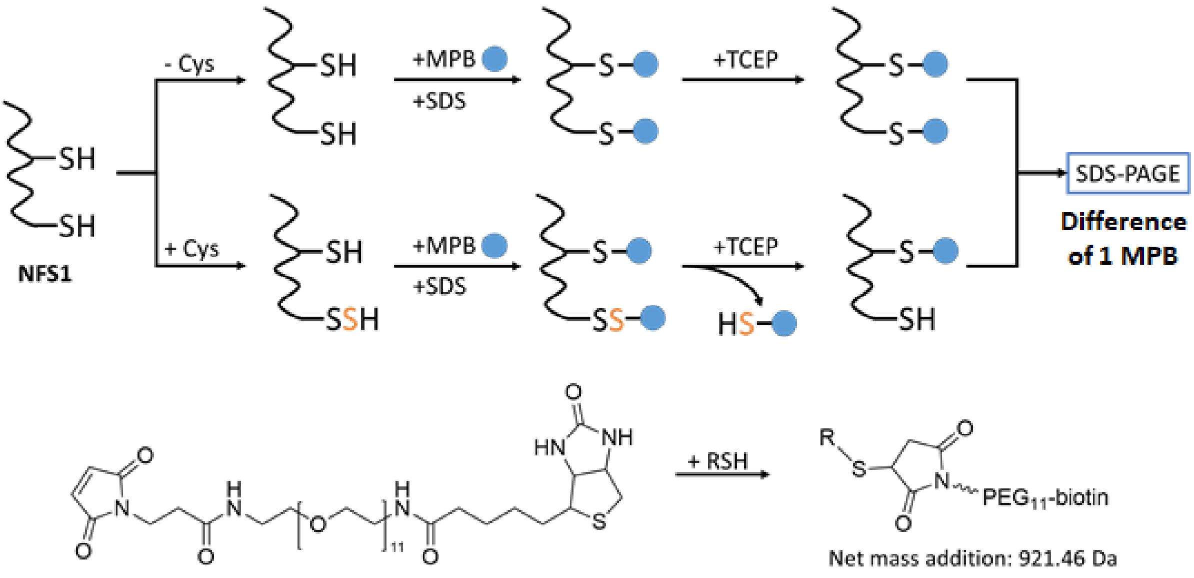

Supplementary Fig. 7. Schematic outline for persulfide detection on NFS1 via an alkylation-based band-shift gel assay. Maleimide-polyethylene-glycol$_{11}$-biotin (MPB; bottom) specifically reacts with free thiol moieties including persulfides at neutral pH. Alkylation by MPB leads to a net mass addition of 921.46 Da per thiol. The reaction scheme on top shows only two of the seven Cys residues of NFS1 for simplicity. Upon incubation with free Cys only one residue, the active-site Cys381, is persulfidated. After MPB labeling of all Cys thiols and the persulfide, the reaction is quenched with SDS. Samples are treated with TCEP for persulfide reduction, and analysed by SDS-PAGE to determine the mass loss by one MPB, i.e. the shift from seven- to six-fold MPB-modified NFS1.

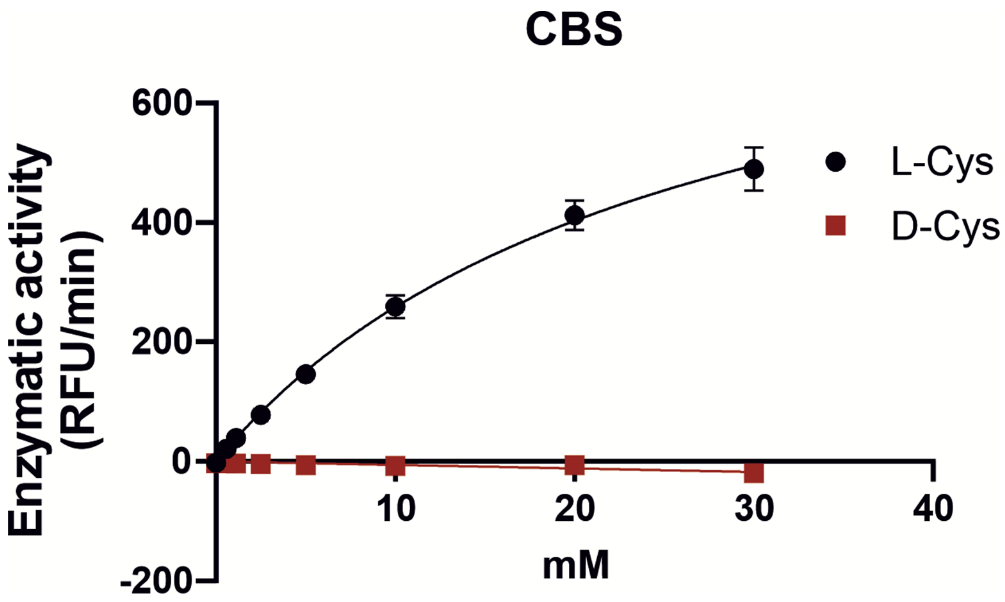

Supplementary Fig. 8. D-Cys is not a substrate of cystathionine β-synthase (CBS). CBS activity was tested as described in Methods in the presence of varying concentration of L- or D-Cys (0-30 mM) and homocysteine (1 mM) in a 96-well plate. H2S production was measured using the H_2S-specific fluorescent probe 7-azido-4-methylcoumarin. Fluorescence (excitation: 365 nm, emission: 450 nm) was measured over two hours at 37 °C. Mean values ± SEM of n=3 biological replicates are shown.

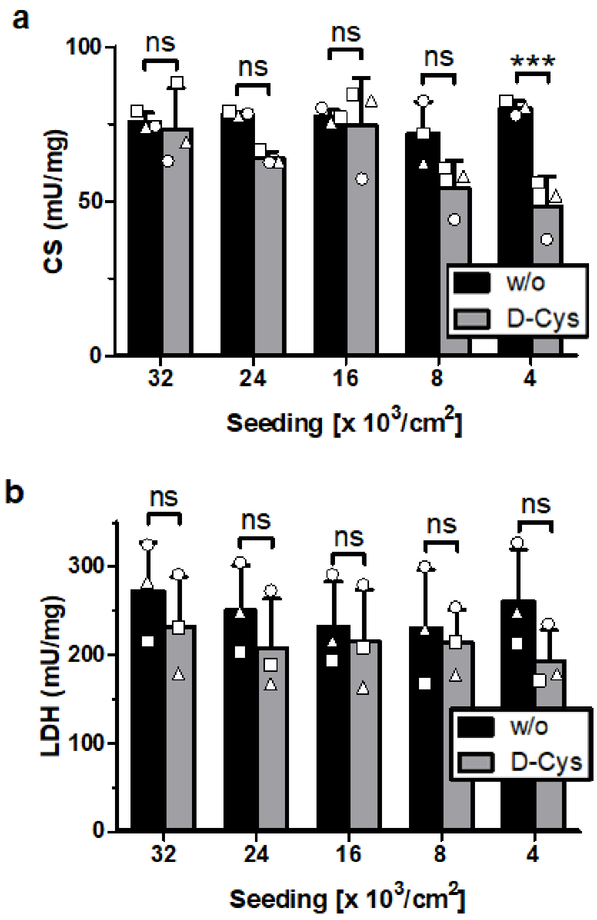

Supplementary Fig. 9. Control enzyme activities for cell density dependence of Fe-S protein defects in D-Cys treated cells. Total cell lysates of cells from Extended Data Fig. 9b were analysed for the specific enzyme activities of (**a**) mitochondrial citrate synthase (CS) and (**b**) cytosolic lactate dehydrogenase (LDH). Data are presented as mean ± SD an analysed by 2-way repeated measures ANOVA and Bonferroni posttests; symbols indicate matching samples of n=3 biological replicates. ****P* < 0.001; ns, not significant.

Supplementary Tables

Supplementary Table 1: Results from the CRISPR/Cas9 screen. A549 cells were transduced, selected and cultured in the presence of 500 µM D-Cys or L-Cys (w/o), as described in Methods. Genomic DNA of the surviving cell clones was extracted and gRNA inserts were identified as described in Methods. The genes identified are ranked in descending order of abundance. See file Suppl. Table S1.xlsx.

Supplementary Table 2: Results from the CRISPR/Cas9 screen as in Supplementary Table 1 except that the top genes are those whose depletion sensitize A549 cells to D-Cys toxicity. The genes are ranked in descending order of abundance. See file Suppl. Table S2.xlsx.

Supplementary Table 3: Analysis of thiol containing metabolites. Thiol containing metabolites including cysteine, cystine, homocysteine, homocystine, reduced glutathione (GSH) and oxidized glutathione (GSSG) from A549 cells treated for 3 days in the absence (w/o) or presence of 500 µM D-Cys have been analysed as explained in Methods. The results expressed in the bottom list are normalized by the amount of protein. Note that this analysis does not allow to differentiate between D- and L-enantiomers of cysteine. See file Suppl. Table S3.xlsx.

Supplementary Table 4: Results from proteomics analysis. A549 cells were cultured for three days in DMEM (w/o) or in the same medium supplemented with either 500 µM D-Cys or 500 µM L-Cys and a proteomic analysis was performed. Page 1 shows comparisons of the proteomes of cells cultured in the absence of D-Cys (w/o) *vs.* with supplemented L-Cys (highlighted in yellow), D-Cys *vs.* w/o (highlighted in green) and L-Cys *vs.* D-Cys (highlighted in blue). On page 2 and 3, the most significant differences between D-Cys *vs.* w/o and D-Cys *vs.* L-Cys are shown respectively. See file Suppl. Table S4.xlsx.

Supplementary Table 5: Results from metabolomics. Comparison of metabolites from A549 cells cultured for three days in the presence of D-Cys or on its absence (w/o). The most enriched metabolites are on an orange background, whereas the most downregulated metabolites are on a green background. Malate and succinate are highlighted in red. See file Suppl. Table S5.xlsx.

Supplementary Table 6: Data collection and refinement statistics of crystallographic experiment.

Wavelength (nm)	0.9796
Resolution range (Å)	48.95 – 2.0 (2.075 – 2.0)
Space group	P 4_1 2_1 2
Unit cell (Å)	*a* = 86.27<br>*b* = 86.27<br>*c* = 246.03
Total reflections	501,654 (48,182)
Unique reflections	63,786 (6269)
Multiplicity	7.9 (7.7)
Completeness (%)	99.97 (99.97)
Mean I/sigma (I)	12.47 (2.86)
Wilson B-factor	25.91
R-merge	0.138 (0.85)
CC ½	0.997 (0.818)
Reflections used in refinement	63782 (6269)
R-work	0.150 (0.247)
R-free	0.186 (0.293)
Number of non-hydrogen atoms<br>Macromolecules<br>Ligands	6126<br>5346<br>363
Protein residues	684
RMS (bonds)	0.019
RMS (angles)	1.6
Ramachandran plot<br>Favored (%)<br>Allowed (%)<br>Outliers (%)	 96.75<br>2.66<br>0.59
Rotamer outliers (%)	1.24
Clashscore	9.22
Average B-factor<br>Macromolecules<br>Ligands	33.88<br>32.59<br>47.02
Number of TLS groups	4
PDB code	8TVT

Supplementary Table 7: Antibodies used in the study.

rabbit anti-solute carrier family 7 member 11 (SLC7A11/xCT):
clone D2M7A, CST (Danvers, USA), cat. # 12691S, validated by the manufacturer and in house by cell phenotyping (this study), dilution 1:1000 to 1500

rabbit anti-solute carrier family 3 member 2 (SLC3A2/CD98):
clone D6O3P, CST (Danvers, USA), cat. # 13180S, validated by the manufacturer and in house by cell phenotyping (this study), dilution 1:200

mouse anti-solute carrier family 3 member 2 (SLC3A2/CD98):
clone E-5, SCBT (Dallas, USA), cat. # sc-376815, validated by the manufacturer and in house by cell phenotyping (this study), dilution 1:2,000

rabbit anti-Nuclear factor erythroid-2-related factor 2 (NRF2):
polyclonal serum, Abcam (Cambridge, GB), cat. # ab137550, validated by the manufacturer and in house by cell phenotyping (this study), dilution 1:1,000

mouse anti-total oxidative phosphorylation complexes (OXPHOS):
antibody Cocktail, Abcam (Cambridge, GB), cat. # ab11041 validated by the manufacturer and in house by cell phenotyping (this study), dilution 1:2,000

mouse anti-NADH:ubiquinone oxidoreductase core subunit S1 (NDUFS1):
clone G-6, SCBT (Dallas, USA), cat. # sc-271510, validated by the manufacturer and in house by cell phenotyping[4], dilution 1:500

mouse anti-NADH:ubiquinone oxidoreductase core subunit S8 (NDUFS8):
clone A-6, SCBT (Dallas, USA), cat. # sc-515527, validated by the manufacturer and in house by cell phenotyping[4], dilution 1:1500

mouse anti-NADH:ubiquinone oxidoreductase core subunit V2 (NDUFV2):
clone B-11, SCBT (Dallas, USA), cat. # sc-515589, validated by the manufacturer and in house by cell phenotyping[4], dilution 1:2500

mouse anti-NADH:ubiquinone oxidoreductase subunit A9 (NDUFA9):
clone 20C11B11B11, Abcam (Cambridge, GB), cat. # ab14713, validated by the manufacturer and in house by cell phenotyping (e.g.,[5] and this study), dilution 1:1,000

mouse anti-NADH:ubiquinone oxidoreductase subunit A13 (NDUFA13, aka GRIM-19):
clone H-10, SCBT (Dallas, USA), cat. # sc-514111, validated by the manufacturer and in house by cell phenotyping (this study), dilution 1:2,000

mouse anti-NADH:ubiquinone oxidoreductase subunit B4 (NDUFB4):
clone 17G3D9E12, Abcam (Cambridge, GB), cat. # ab110243, validated by the manufacturer and in house by cell phenotyping (e.g.,[6] and this study), dilution 1:500

mouse anti-NADH:ubiquinone oxidoreductase subunit B6 (NDUFB6):
clone 21C11BC11, Abcam (Cambridge, GB), cat. # ab110244, validated by the manufacturer and in house by cell phenotyping (e.g.,[5] and this study), dilution 1:2,000

mouse anti-Complex II subunit 30 kDa Ip (SDHB):
clone G-10, SCBT (Dallas, USA), cat. # sc-271548, validated by the manufacturer and in house by cell phenotyping[4], dilution 1:1,000

rabbit anti-Ubiquinol-Cytochrome C Reductase - Rieske Iron-Sulfur Polypeptide 1 (UQCRFS1):
polyclonal serum, raised against bovine UQCRFS1, validated and provided by H. Schägger and I. Wittig (Frankfurt, Germany), validated in house[7], final dilution 1:2,000

rabbit anti- ubiquinol-cytochrome c reductase core protein 2 (UQCRC2):
polyclonal serum, raised against bovine UQCRC2, validated and provided by H. Schägger and I. Wittig (Frankfurt, Germany), validated in house (unpublished), final dilution 1:2500

rabbit anti-cytochrome c oxidase II (mitochondrially encoded, MT-CO2):
polyclonal serum, raised against bovine MT-CO2, validated and provided by H. Schägger and I. Wittig (Frankfurt, Germany), validated in house[7], final dilution 1:2,000

rabbit anti-cytochrome c oxidase subunit 6A/B (COX6A/B):
polyclonal serum, raised against bovine COX6A/B, validated and provided by H. Schägger and I. Wittig (Frankfurt, Germany), validated in house[7], final dilution 1:5,000

rabbit anti-Complex V subunits ATP5F1A/B:
polyclonal serum, raised against bovine ATP5F1A/B, validated and provided by H. Schägger and I. Wittig (Frankfurt, Germany), validated in house (e.g.,[7]), dilution 1:1500

rabbit anti-Complex V subunit ATP8 (mitochondrially encoded):
polyclonal serum (affinity purified), Protein Tech Group (Rosemont, USA), cat. # 26723-1-AP, validated by the manufacturer and in house by cell phenotyping (unpublished), dilution 1:1,000

mouse anti-cysteine desulfurase 1 (NFS1):
clone B-7, SCBT (Dallas, USA), cat. # sc-365308, validated by the manufacturer and in house by cell phenotyping (unpublished), dilution 1:400

rabbit anti-aconitase 2 (ACO2):
polyclonal serum, Invitrogen (Waltham, USA), cat. # PA5-29037, validated by the manufacturer and in house[6] and this study, dilution 1:2,500

rabbit anti-ferrochelatase (FECH):
polyclonal serum, raised against human FECH, validated and provided by H. and T. Dailey (Athens, Georgia, USA), validated in house (e.g.,[8]), dilution 1:2,000

rabbit anti-lipoic acid synthetase (LIAS):
polyclonal serum, Protein Tech Group (Rosemont, USA), cat. # 11577-1-AP, validated by the manufacturer and in house[4], dilution 1:500

rabbit anti-lipoic acid:
polyclonal serum, Merck-Calbiochem (Darmstadt, Germany), cat. # 1077-28-7, validated in house[4], dilution 1:1,000

mouse anti-dihydrolipoamide S-acetyltransferase (DLAT, PDC-E2):
clone 4A4-B6-C10, CST (Danvers, USA), cat. # 12362S, validated by the manufacturer and in house by cell phenotyping (unpublished), dilution 1:2,500

rabbit anti-voltage dependent anion channel 1 (VDAC1, Porin):
polyclonal serum, Protein Tech Group (Rosemont, USA), cat. # 55259-1-AP, validated by the manufacturer and in house[6], dilution 1:1500 and polyclonal serum, CST (Danvers, USA), cat. # 4661S, validated by the manufacturer and in house by cell phenotyping (unpublished), dilution 1:1,000

rabbit anti-cytosolic iron-sulfur assembly component 3 (CIAO3, aka NARF-L / IOP1):
affinity-purified polyclonal serum, raised against human CIAO3, validated in house by CIAO3 overproduction and immunoprecipitation [9]), dilution 1:25

rabbit anti-cytosolic iron-sulfur assembly component 1 (CIAO1):
affinity-purified polyclonal serum, raised against human CIAO1, validated in house by CIAO1 depletion, overproduction, immunoprecipitation, and cell fractionation[10], dilution 1:500

rabbit anti-cytosolic iron-sulfur assembly component MMS19:
polyclonal serum, raised against human MMS19, validated in house by MMS19 depletion[11], dilution 1:750

rabbit anti-glutamine phosphoribosyl pyrophosphate amidotransferase (GPAT, aka PPAT):
polyclonal serum, raised against human GPAT, validated in house by comparison with affinity purified anti-GPAT serum from H. Puccio[12], dilution 1:500

rabbit anti-dihydropyrimidine dehydrogenase (DPYD):
polyclonal serum, Protein Tech Group (Rosemont, USA), cat. # 27662-1-AP, validated by the manufacturer and in house by cell phenotyping (unpublished), dilution 1:1,000

rabbit anti-DNA polymerase delta catalytic subunit 1 (POLD1):
polyclonal serum, Protein Tech Group (Rosemont, USA), cat. # 15646-1-AP, validated by the manufacturer and in house by cell phenotyping[10], dilution 1:1,000

rabbit anti-nth like DNA glycosylase 1 (NTHL1):
affinity-purified polyclonal serum, raised against human NTHL1, validated in house by cell phenotyping, NTHL1 overproduction and immunoprecipitation[9], dilution 1:1,000

mouse anti-iron regulatory protein 1 (IRP1):
clone 295B; validated and provided by R. Eisenstein (Wisconsin, USA), validated in house, e.g., by detecting an IRE-binding antigen (unpublished), dilution 1:3,000

mouse anti-iron regulatory protein 2 (IRP2):
clone 7H6, SCBT (Dallas, USA), cat. # sc-33682, validated by the manufacturer and in house by cell phenotyping, dilution 1:250

mouse anti-E-cadherin (ECAD):
clone 36, BD Biosciences (Franklin Lakes, USA), cat. # 610181, validated by the manufacturer and in house by cell phenotyping (unpublished), dilution 1:1,000

rabbit anti–γ-H2AX
Polyclonal serum raised against a synthetic peptide from Histone γ-H2AX
ab11175 (Abcam, Cambridge, UK), validated in[13], dilution: 1:200

mouse anti-53BP1
Home made hybridoma supernatant, dilution 1:50, validated in[14].

mouse anti-β-actin−Peroxidase (ACTB):
clone AC-15, Sigma-Aldrich (St. Louis, USA), cat. # A3854, validated by the manufacturer and in[15], dilution 1:50,000

mouse anti-tubulin (TUBA):
clone DM1A, Sigma-Aldrich (St. Louis, USA), cat. # T9026, validated by the manufacturer and in house by immunofluorescence (unpublished), dilution 1:3,000

mouse anti-β-tubulin (TUBB):
clone Tub 2.1, Sigma-Aldrich (St. Louis, USA), cat. # T4026, validated by the manufacturer and in house by cell phenotyping (unpublished), dilution 1:2,000

mouse anti-DYKDDDDK (FLAG):
clone M2, Sigma-Aldrich (St. Louis, USA), cat. # F1804, validated by the manufacturer and in house (this study), dilution 1:1,000

Donkey anti-rabbit IgG Alexa Fluor® 488:
Polyclonal serum cat. #711-545-152, Jackson ImmunoResearch (West Grove, USA), validated by the manufacturer, dilution 1:300

Legend for Supplementary File 1

The Chimera X file (L_D-Cys_Cycle_final.X.py) can be used for a 3D rendition of the cartoons presented in Fig. 5b,d and Extended Data Fig. 7a,d. The presentation compares the relative steric positioning of the external L- and D-Cys-aldimines and -ketimines (sulfur donor) to the catalytic Cys381^{NFS1} (sulfur-acceptor). The 3D scenario also shows the positioning of the catalytic His156^{NFS1} in the various structures.

Supplementary References

1 Lill, R. & Freibert, S. A. Mechanisms of Mitochondrial Iron-Sulfur Protein Biogenesis. *Annu Rev Biochem* **89**, 471-499, doi:10.1146/annurev-biochem-013118-111540 (2020).

2 Netz, D. J., Mascarenhas, J., Stehling, O., Pierik, A. J. & Lill, R. Maturation of cytosolic and nuclear iron-sulfur proteins. *Trends Cell Biol* **24**, 303-312, doi:10.1016/j.tcb.2013.11.005 (2014).

3 Anderson, C. P., Shen, M., Eisenstein, R. S. & Leibold, E. A. Mammalian iron metabolism and its control by iron regulatory proteins. *Biochim Biophys Acta* **1823**, 1468-1483, doi:10.1016/j.bbamcr.2012.05.010 (2012).

4 Schulz, V. *et al.* Functional spectrum and specificity of mitochondrial ferredoxins FDX1 and FDX2. *Nat Chem Biol* **19**, 206-217, doi:10.1038/s41589-022-01159-4 (2023).

5 Sheftel, A. D. *et al.* Human ind1, an iron-sulfur cluster assembly factor for respiratory complex I. *Mol Cell Biol* **29**, 6059-6073, doi:10.1128/MCB.00817-09 (2009).

6 Torraco, A. *et al.* ISCA1 mutation in a patient with infantile-onset leukodystrophy causes defects in mitochondrial [4Fe-4S] proteins. *Hum Mol Genet* **27**, 3650, doi:10.1093/hmg/ddy273 (2018).

7 Sheftel, A. D. *et al.* The human mitochondrial ISCA1, ISCA2, and IBA57 proteins are required for [4Fe-4S] protein maturation. *Mol Biol Cell* **23**, 1157-1166, doi:10.1091/mbc.E11-09-0772 (2012).

8 Sheftel, A. D. *et al.* Humans possess two mitochondrial ferredoxins, Fdx1 and Fdx2, with distinct roles in steroidogenesis, heme, and Fe/S cluster biosynthesis. *Proc Natl Acad Sci U S A* **107**, 11775-11780, doi:10.1073/pnas.1004250107 (2010).

9 Stehling, O. *et al.* Function and crystal structure of the dimeric P-loop ATPase CFD1 coordinating an exposed [4Fe-4S] cluster for transfer to apoproteins. *Proc Natl Acad Sci U S A* **115**, E9085-E9094, doi:10.1073/pnas.1807762115 (2018).

10 Stehling, O. *et al.* Human CIA2A-FAM96A and CIA2B-FAM96B integrate iron homeostasis and maturation of different subsets of cytosolic-nuclear iron-sulfur proteins. *Cell Metab* **18**, 187-198, doi:10.1016/j.cmet.2013.06.015 (2013).

11 Stehling, O. *et al.* MMS19 assembles iron-sulfur proteins required for DNA metabolism and genomic integrity. *Science* **337**, 195-199, doi:10.1126/science.1219723 (2012).

12 Martelli, A. *et al.* Frataxin is essential for extramitochondrial Fe-S cluster proteins in mammalian tissues. *Hum Mol Genet* **16**, 2651-2658, doi:10.1093/hmg/ddm163 (2007).

13 Xiao, S. Y. *et al.* LncRNA DLGAP1-AS2 promotes the radioresistance of rectal cancer stem cells by upregulating CD151 expression via E2F1. *Transl Oncol* **18**, 101304, doi:10.1016/j.tranon.2021.101304 (2022).

14 Schultz, L. B., Chehab, N. H., Malikzay, A. & Halazonetis, T. D. p53 binding protein 1 (53BP1) is an early participant in the cellular response to DNA double-strand breaks. *J Cell Biol* **151**, 1381-1390, doi:10.1083/jcb.151.7.1381 (2000).

15 De La Rossa, A. *et al.* Paradoxical neuronal hyperexcitability in a mouse model of mitochondrial pyruvate import deficiency. *Elife* **11**, doi:10.7554/eLife.72595 (2022).
