# Supplementary material for: d-cysteine impairs tumour growth by inhibiting cysteine desulfurase NFS1
Source: Nat Metab. 2025 Aug 12;7(8):1646–62. doi: 10.1038/s42255-025-01339-1 (PMC12373508; doi:10.1038/s42255-025-01339-1)
Supplement: Supplementary file 2 — Reporting Summary [file 42255_2025_1339_MOESM2_ESM.pdf]

nature portfolio

Corresponding author(s): Roland Lill and Jean-Claude Martinou

Last updated by author(s): May 28, 2025

Reporting Summary

Nature Portfolio wishes to improve the reproducibility of the work that we publish. This form provides structure for consistency and transparency in reporting. For further information on Nature Portfolio policies, see our Editorial Policies and the Editorial Policy Checklist.

Statistics

For all statistical analyses, confirm that the following items are present in the figure legend, table legend, main text, or Methods section.

n/a	Confirmed	
☐	☒	The exact sample size (*n*) for each experimental group/condition, given as a discrete number and unit of measurement
☐	☒	A statement on whether measurements were taken from distinct samples or whether the same sample was measured repeatedly
☐	☒	The statistical test(s) used AND whether they are one- or two-sided<br>*Only common tests should be described solely by name; describe more complex techniques in the Methods section.*
☒	☐	A description of all covariates tested
☒	☐	A description of any assumptions or corrections, such as tests of normality and adjustment for multiple comparisons
☐	☒	A full description of the statistical parameters including central tendency (e.g. means) or other basic estimates (e.g. regression coefficient) AND variation (e.g. standard deviation) or associated estimates of uncertainty (e.g. confidence intervals)
☐	☒	For null hypothesis testing, the test statistic (e.g. *F*, *t*, *r*) with confidence intervals, effect sizes, degrees of freedom and *P* value noted<br>*Give P values as exact values whenever suitable.*
☒	☐	For Bayesian analysis, information on the choice of priors and Markov chain Monte Carlo settings
☒	☐	For hierarchical and complex designs, identification of the appropriate level for tests and full reporting of outcomes
☒	☐	Estimates of effect sizes (e.g. Cohen's *d*, Pearson's *r*), indicating how they were calculated

Our web collection on statistics for biologists contains articles on many of the points above.

Software and code

Policy information about availability of computer code

Data collection	ESRF BEAMLINE ID14-4
Data analysis	XDS and CCP4 package, SHELX-CDE, ARP/wARP version 7, COOT 0.8.1, REFMAC as part of the CCP4 package, Origin 8G, Prism 5; Agilent Quantitative analysis software (version B.07.00, MassHunter Agilent technologies) ; LOWESS/Spline algorithm; MaxQuant 1.6.10.43 ; PHENIX software package.

For manuscripts utilizing custom algorithms or software that are central to the research but not yet described in published literature, software must be made available to editors and reviewers. We strongly encourage code deposition in a community repository (e.g. GitHub). See the Nature Portfolio guidelines for submitting code & software for further information.

Data

Policy information about availability of data

All manuscripts must include a data availability statement. This statement should provide the following information, where applicable:

- Accession codes, unique identifiers, or web links for publicly available datasets
- A description of any restrictions on data availability
- For clinical datasets or third party data, please ensure that the statement adheres to our policy

Provide your data availability statement here.

Research involving human participants, their data, or biological material

Policy information about studies with human participants or human data. See also policy information about sex, gender (identity/presentation), and sexual orientation and race, ethnicity and racism.

Reporting on sex and gender	N/A
Reporting on race, ethnicity, or other socially relevant groupings	N/A
Population characteristics	N/A
Recruitment	N/A
Ethics oversight	N/A

Note that full information on the approval of the study protocol must also be provided in the manuscript.

Field-specific reporting

Please select the one below that is the best fit for your research. If you are not sure, read the appropriate sections before making your selection.

☒ Life sciences ☐ Behavioural & social sciences ☐ Ecological, evolutionary & environmental sciences

For a reference copy of the document with all sections, see nature.com/documents/nr-reporting-summary-flat.pdf

Life sciences study design

All studies must disclose on these points even when the disclosure is negative.

Sample size	Sample size (n) was counted and represents biological repetitions of the respective experiment. Routine sample size was n=3, but often experiments were performed more frequently.
Data exclusions	One control mouse was found dead in its cage two days after treatment randomization. The tumor was poorly developed, and the cause of death was not further investigated. This animal was excluded from the final analysis.
Replication	All attempts at replication were successful. The number of replications is stated for each experiment individually in the figure legends
Randomization	Mice were randomized after tumour cell implantation, before treatment.
Blinding	No blinding was done. However, at Transcure, the technicians responsible for handling the mice were aware of the general nature of the treatments they administered, but did not know their exact composition or intended effects.

Behavioural & social sciences study design

All studies must disclose on these points even when the disclosure is negative.

Study description	*Briefly describe the study type including whether data are quantitative, qualitative, or mixed-methods (e.g. qualitative cross-sectional, quantitative experimental, mixed-methods case study).*
Research sample	*State the research sample (e.g. Harvard university undergraduates, villagers in rural India) and provide relevant demographic information (e.g. age, sex) and indicate whether the sample is representative. Provide a rationale for the study sample chosen. For studies involving existing datasets, please describe the dataset and source.*

Sampling strategy	*Describe the sampling procedure (e.g. random, snowball, stratified, convenience). Describe the statistical methods that were used to predetermine sample size OR if no sample-size calculation was performed, describe how sample sizes were chosen and provide a rationale for why these sample sizes are sufficient. For qualitative data, please indicate whether data saturation was considered, and what criteria were used to decide that no further sampling was needed.*
Data collection	*Provide details about the data collection procedure, including the instruments or devices used to record the data (e.g. pen and paper, computer, eye tracker, video or audio equipment) whether anyone was present besides the participant(s) and the researcher, and whether the researcher was blind to experimental condition and/or the study hypothesis during data collection.*
Timing	*Indicate the start and stop dates of data collection. If there is a gap between collection periods, state the dates for each sample cohort.*
Data exclusions	*If no data were excluded from the analyses, state so OR if data were excluded, provide the exact number of exclusions and the rationale behind them, indicating whether exclusion criteria were pre-established.*
Non-participation	*State how many participants dropped out/declined participation and the reason(s) given OR provide response rate OR state that no participants dropped out/declined participation.*
Randomization	*If participants were not allocated into experimental groups, state so OR describe how participants were allocated to groups, and if allocation was not random, describe how covariates were controlled.*

Ecological, evolutionary & environmental sciences study design

All studies must disclose on these points even when the disclosure is negative.

Study description	*Briefly describe the study. For quantitative data include treatment factors and interactions, design structure (e.g. factorial, nested, hierarchical), nature and number of experimental units and replicates.*
Research sample	*Describe the research sample (e.g. a group of tagged Passer domesticus, all Stenocereus thurberi within Organ Pipe Cactus National Monument), and provide a rationale for the sample choice. When relevant, describe the organism taxa, source, sex, age range and any manipulations. State what population the sample is meant to represent when applicable. For studies involving existing datasets, describe the data and its source.*
Sampling strategy	*Note the sampling procedure. Describe the statistical methods that were used to predetermine sample size OR if no sample-size calculation was performed, describe how sample sizes were chosen and provide a rationale for why these sample sizes are sufficient.*
Data collection	*Describe the data collection procedure, including who recorded the data and how.*
Timing and spatial scale	*Indicate the start and stop dates of data collection, noting the frequency and periodicity of sampling and providing a rationale for these choices. If there is a gap between collection periods, state the dates for each sample cohort. Specify the spatial scale from which the data are taken*
Data exclusions	*If no data were excluded from the analyses, state so OR if data were excluded, describe the exclusions and the rationale behind them, indicating whether exclusion criteria were pre-established.*
Reproducibility	*Describe the measures taken to verify the reproducibility of experimental findings. For each experiment, note whether any attempts to repeat the experiment failed OR state that all attempts to repeat the experiment were successful.*
Randomization	Mice were randomized after tumour cell implantation, before treatment.
Blinding	*Describe the extent of blinding used during data acquisition and analysis. If blinding was not possible, describe why OR explain why blinding was not relevant to your study.*

Did the study involve field work? ☐ Yes ☐ No

Field work, collection and transport

Field conditions	*Describe the study conditions for field work, providing relevant parameters (e.g. temperature, rainfall).*
Location	*State the location of the sampling or experiment, providing relevant parameters (e.g. latitude and longitude, elevation, water depth).*
Access & import/export	*Describe the efforts you have made to access habitats and to collect and import/export your samples in a responsible manner and in compliance with local, national and international laws, noting any permits that were obtained (give the name of the issuing authority, the date of issue, and any identifying information).*
Disturbance	*Describe any disturbance caused by the study and how it was minimized.*

Reporting for specific materials, systems and methods

We require information from authors about some types of materials, experimental systems and methods used in many studies. Here, indicate whether each material, system or method listed is relevant to your study. If you are not sure if a list item applies to your research, read the appropriate section before selecting a response.

Materials & experimental systems

n/a	Involved in the study
☐	☒ Antibodies
☐	☒ Eukaryotic cell lines
☒	☐ Palaeontology and archaeology
☐	☒ Animals and other organisms
☒	☐ Clinical data
☒	☐ Dual use research of concern
☒	☐ Plants

Methods

n/a	Involved in the study
☒	☐ ChIP-seq
☐	☒ Flow cytometry
☒	☐ MRI-based neuroimaging

Antibodies

Antibodies used	rabbit anti-solute carrier family 7 member 11 (SLC7A11/xCT), clone D2M7A, CST (Danvers, USA), cat. # 12691S, dilution 1:1500; clone 12691S (Cell Signaling Technology), dilution 1:1,000; rabbit anti-solute carrier family 3 member 2 (SLC3A2/CD98), clone D6O3P, CST (Danvers, USA), cat. # 13180S, dilution 1:200; mouse anti-solute carrier family 3 member 2 (SLC3A2/CD98), clone E-5, SCBT (Dallas, USA), cat. # sc-376815, dilution 1:2,000; rabbit anti-Nuclear factor erythroid-2-related factor 2 (NRF2), polyclonal serum, Abcam (Cambridge, GB), cat. # ab137550, dilution 1:1,000; mouse anti-total oxidative phosphorylation complexes (OXPHOS), antibody Cocktail, Abcam (Cambridge, GB), cat. # ab11041, dilution 1:2,000; mouse anti-NADH:ubiquinone oxidoreductase core subunit S1 (NDUFS1), clone G-6, SCBT (Dallas, USA), cat. # sc-271510, dilution 1:500; mouse anti-NADH:ubiquinone oxidoreductase core subunit S8 (NDUFS8), clone A-6, SCBT (Dallas, USA), cat. # sc-515527, dilution 1:1500; mouse anti-NADH:ubiquinone oxidoreductase core subunit V2 (NDUFV2), clone B-11, SCBT (Dallas, USA), cat. # sc-515589, dilution 1:2500; mouse anti-NADH:ubiquinone oxidoreductase subunit A9 (NDUFA9), clone 20C11B11B11, Abcam (Cambridge, GB), cat. # ab14713, dilution 1:1,000; mouse anti-NADH:ubiquinone oxidoreductase subunit A13 (NDUFA13, aka GRIM-19), clone H-10, SCBT (Dallas, USA), cat. # sc-514111, dilution 1:2,000; mouse anti-NADH:ubiquinone oxidoreductase subunit B4 (NDUFB4), clone 17G3D9E12, Abcam (Cambridge, GB), cat. # ab110243, dilution 1:500; mouse anti-NADH:ubiquinone oxidoreductase subunit B6 (NDUFB6), clone 21C11BC11, Abcam (Cambridge, GB), cat. # ab110244,dilution 1:2,000; mouse anti-Complex II subunit 30 kDa Ip (SDHB),clone G-10, SCBT (Dallas, USA), cat. # sc-271548, dilution 1:1,000; rabbit anti-Ubiquinol-Cytochrome C Reductase - Rieske Iron-Sulfur Polypeptide 1 (UQCRFS1), polyclonal serum, raised against bovine UQCRFS1, final dilution 1:2,000; rabbit anti- ubiquinol-cytochrome c reductase core protein 2 (UQCRC2), polyclonal serum, raised against bovine UQCRC2, final dilution 1:2500; rabbit anti-cytochrome c oxidase II (mitochondrially encoded, MT-CO2), polyclonal serum, raised against bovine MT-CO2, final dilution 1:2,000; rabbit anti-cytochrome c oxidase subunit 6A/B (COX6A/B), polyclonal serum, raised against bovine COX6A/B, final dilution 1:5,000; rabbit anti-Complex V subunits ATP5F1A/B, polyclonal serum, raised against bovine ATP5F1A/B, dilution 1:1500; rabbit anti-Complex V subunit ATP8 (mitochondrially encoded), polyclonal serum (affinity purified), Protein Tech Group (Rosemont, USA), cat. # 26723-1-AP, dilution 1:1,000; mouse anti-cysteine desulfurase 1 (NFS1), clone B-7, SCBT (Dallas, USA), cat. # sc-365308, dilution 1:400; rabbit anti-aconitase 2 (ACO2), polyclonal serum, Invitrogen (Waltham, USA), cat. # PA5-29037, dilution 1:2,500; rabbit anti-ferrochelatase (FECH), polyclonal serum, raised against human FECH, validated in house (e. g. 10.1073/pnas.1004250107), dilution 1:2,000; rabbit anti-lipoic acid synthetase (LIAS), polyclonal serum, Protein Tech Group (Rosemont, USA), cat. # 11577-1-AP, dilution 1:500; rabbit anti-lipoic acid, polyclonal serum, Merck-Calbiochem (Darmstadt, Germany), cat. # 1077-28-7, dilution 1:1,000; mouse anti-dihydrolipoamide S-acetyltransferase (DLAT, PDC-E2), clone 4A4-B6-C10, CST (Danvers, USA), cat. # 12362S, dilution 1:2,500; rabbit anti-voltage dependent anion channel 1 (VDAC1, Porin), polyclonal serum, Protein Tech Group (Rosemont, USA), cat. # 55259-1-AP, dilution 1:1500 and polyclonal serum, CST (Danvers, USA), cat. # 4661S, dilution 1:1,000; rabbit anti-cytosolic iron-sulfur assembly component 3 (CIAO3, aka NARF-L / IOP1), affinity-purified polyclonal serum, raised against human CIAO3, dilution 1:25; rabbit anti-cytosolic iron-sulfur assembly component 1 (CIAO1), affinity-purified polyclonal serum, raised against human CIAO1, dilution 1:500; rabbit anti-cytosolic iron-sulfur assembly component MMS19; polyclonal serum, raised against human MMS19, dilution 1:750; rabbit anti-glutamine phosphoribosyl pyrophosphate amidotransferase (GPAT, aka PPAT):<br>polyclonal serum, raised against human GPAT, dilution 1:500; rabbit anti-dihydropyrimidine dehydrogenase (DPYD), polyclonal serum, Protein Tech Group (Rosemont, USA), cat. # 27662-1-AP, dilution 1:1,000; rabbit anti-DNA polymerase delta catalytic subunit 1 (POLD1), polyclonal serum, Protein Tech Group (Rosemont, USA), cat. # 15646-1-AP, dilution 1:1,000; rabbit anti-nth like DNA glycosylase 1 (NTHL1), affinity-purified polyclonal serum, raised against human NTHL1, dilution 1:1,000; mouse anti-iron regulatory protein 1 (IRP1), clone 295B, dilution 1:3,000; mouse anti-iron regulatory protein 2 (IRP2),clone 7H6, SCBT (Dallas, USA), cat. # sc-33682, dilution 1:250; mouse anti-E-cadherin (ECAD), clone 36, BD Biosciences (Franklin Lakes, USA), cat. # 610181, dilution 1:1,000; rabbit anti–H2AX, Polyclonal serum raised against a synthetic peptide from Histone H2AX, # ab11175 (Abcam, Cambridge, UK), dilution: 1:200; mouse anti-53BP; home made hybridoma supernatant, dilution 1:50.; mouse anti-β-actin–Peroxidase (ACTB); clone AC-15, Sigma-Aldrich (St. Louis, USA), cat. # A3854, dilution 1:50,000; mouse anti-tubulin (TUBA),clone DM1A, Sigma-Aldrich (St. Louis, USA), cat. # T9026, dilution 1:3,000; mouse anti-β-tubulin (TUBB), clone Tub 2.1, Sigma-Aldrich (St. Louis, USA), cat. # T4026, dilution 1:2,000; mouse anti-DYKDDDDK (FLAG), clone M2, Sigma-Aldrich (St. Louis, USA), cat. # F1804; Donkey anti-rabbit IgG Alexa Fluor® 488, cat. #711-545-152, Jackson ImmunoResearch (West Grove, USA), dilution 1:300
Validation	rabbit anti-solute carrier family 7 member 11 (SLC7A11/xCT) validated by the manufacturer and in house by cell phenotyping (this study) and clone 12691S (Cell Signaling Technology) validated by the manufacturer and in house by cell phenotyping (this study);

rabbit anti-solute carrier family 3 member 2 (SLC3A2/CD98) validated by the manufacturer and in house by cell phenotyping (this study); mouse anti-solute carrier family 3 member 2 (SLC3A2/CD98) validated by the manufacturer and in house by cell phenotyping (this study); rabbit anti-Nuclear factor erythroid-2-related factor 2 (NRF2), validated by the manufacturer and in house by cell phenotyping (this study); mouse anti-total oxidative phosphorylation complexes (OXPHOS) validated by the manufacturer and in house by cell phenotyping (this study); mouse anti-NADH:ubiquinone oxidoreductase core subunit S1 (NDUFS1): validated by the manufacturer and in house by cell phenotyping (10.1038/s41589-022-01159-4); mouse anti-NADH:ubiquinone oxidoreductase core subunit S8 (NDUFS8) validated by the manufacturer and in house by cell phenotyping (10.1038/s41589-022-01159-4); mouse anti-NADH:ubiquinone oxidoreductase core subunit V2 (NDUFV2) validated by the manufacturer and in house by cell phenotyping (10.1038/s41589-022-01159-4); mouse anti-NADH:ubiquinone oxidoreductase subunit A9 (NDUFA9) validated by the manufacturer and in house by cell phenotyping (e. g. 10.1128/MCB.00817-09 and this study); mouse anti-NADH:ubiquinone oxidoreductase subunit A13 (NDUFA13, aka GRIM-19) validated by the manufacturer and in house by cell phenotyping (this study); mouse anti-NADH:ubiquinone oxidoreductase subunit B4 (NDUFB4), validated by the manufacturer and in house by cell phenotyping (e.g., 10.1093/hmg/ddy183 and this study); mouse anti-NADH:ubiquinone oxidoreductase subunit B6 (NDUFB6) validated by the manufacturer and in house by cell phenotyping (e.g., 10.1128/MCB.00817-09 and this study); mouse anti-Complex II subunit 30 kDa Ip (SDHB):
validated by the manufacturer and in house by cell phenotyping (10.1038/s41589-022-01159-4);
rabbit anti-Ubiquinol-Cytochrome C Reductase - Rieske Iron-Sulfur Polypeptide 1 (UQCRFS1):
validated and provided by H. Schägger and I. Wittig (Frankfurt, Germany), validated in house (10.1091/mbc.E11-09-0772); rabbit anti-ubiquinol-cytochrome c reductase core protein 2 (UQCRC2), validated and provided by H. Schägger and I. Wittig (Frankfurt, Germany), validated in house (unpublished); rabbit anti-cytochrome c oxidase II (mitochondrially encoded, MT-CO2):
validated and provided by H. Schägger and I. Wittig (Frankfurt, Germany), validated in house (10.1091/mbc.E11-09-0772); rabbit anti-cytochrome c oxidase subunit 6A/B (COX6A/B):
validated and provided by H. Schägger and I. Wittig (Frankfurt, Germany), validated in house (10.1091/mbc.E11-09-0772); rabbit anti-Complex V subunits ATP5F1A/B validated and provided by H. Schägger and I. Wittig (Frankfurt, Germany), validated in house (e. g. 10.1091/mbc.E11-09-0772); rabbit anti-Complex V subunit ATP8 (mitochondrially encoded) validated by the manufacturer and in house by cell phenotyping (unpublished); mouse anti-cysteine desulfurase 1 (NFS1) validated by the manufacturer and in house by cell phenotyping (unpublished); rabbit anti-aconitase 2 (ACO2):
polyclonal serum, Invitrogen (Waltham, USA), cat. # PA5-29037, validated by the manufacturer and in house (10.1093/hmg/ddy183 and this study); rabbit anti-ferrochelatase (FECH) validated and provided by H. and T. Dailey (Athens, Georgia, USA), validated in house (e. g. 10.1073/pnas.1004250107); rabbit anti-lipoic acid synthetase (LIAS) validated by the manufacturer and in house (10.1038/s41589-022-01159-4), dilution 1:500; rabbit anti-lipoic acid validated in house (10.1038/s41589-022-01159-4); mouse anti-dihydrolipoamide S-acetyltransferase (DLAT, PDC-E2) validated by the manufacturer and in house by cell phenotyping (unpublished); rabbit anti-voltage dependent anion channel 1 (VDAC1, Porin) validated by the manufacturer and in house (doi: 10.1093/hmg/ddy183), dilution 1:1500 and polyclonal serum validated by the manufacturer and in house by cell phenotyping (unpublished); rabbit anti-cytosolic iron-sulfur assembly component 3 (CIAO3, aka NARF-L / IOP1) validated in house by CIAO3 overproduction and immunoprecipitation (10.1073/pnas.1807762115); rabbit anti-cytosolic iron-sulfur assembly component 1 (CIAO1) validated in house by CIAO1 depletion, overproduction, immunoprecipitation, and cell fractionation (10.1016/j.cmet.2013.06.015); rabbit anti-cytosolic iron-sulfur assembly component MMS19 validated in house by MMS19 depletion (10.1126/science.1219723); rabbit anti-glutamine phosphoribosyl pyrophosphate amidotransferase (GPAT, aka PPAT), validated in house by comparison with affinity purified anti-GPAT serum from H. Puccio (Illkirch, France; 10.1093/hmg/ddm163); rabbit anti-dihydropyrimidine dehydrogenase (DPYD) validated by the manufacturer and in house by cell phenotyping (unpublished); rabbit anti-DNA polymerase delta catalytic subunit 1 (POLD1) validated by the manufacturer and in house by cell phenotyping (10.1016/j.cmet.2013.06.015); rabbit anti-nth like DNA glycosylase 1 (NTHL1) validated in house by cell phenotyping, NTHL1 overproduction and immunoprecipitation (10.1073/pnas.1807762115); mouse anti-iron regulatory protein 1 (IRP1) validated in house e. g. by detecting an IRE-binding antigen (unpublished); mouse anti-iron regulatory protein 2 (IRP2) validated by the manufacturer and in house by cell phenotyping; mouse anti-E-cadherin (ECAD) validated by the manufacturer and in house by cell phenotyping (unpublished); rabbit anti–H2AX, validated in 10.1016/j.tranon.2021.101304
mouse anti-53BP1 in house made hybridoma supernatant, validated by Linda B. et al., J. Cell Biol. 151(7): 1381–1390, 2000, 10.1083/jcb.151.7.1381); mouse anti-β-actin–Peroxidase (ACTB):
validated by the manufacturer and in 10.7554/eLife.72595; mouse anti-tubulin (TUBA), validated by the manufacturer and in house by immunofluorescence (unpublished), dilution 1:3,000; mouse anti-β-tubulin (TUBB) validated by the manufacturer and in house by cell phenotyping (unpublished); mouse anti-DYKDDDDK (FLAG) validated by the manufacturer and in house (this study); Donkey anti-rabbit IgG Alexa Fluor® 488, validated by the manufacturer.

Eukaryotic cell lines

Policy information about cell lines and Sex and Gender in Research

Cell line source(s)	Human lung adenocarcinoma A549 cells (ATCC CCL-185, gift from Pr Paul Hofman lab, IRCAN, Nice, France), human breast adenocarcinoma MDA-MB-231 cells (gift from Pr Didier Picard lab, University of Geneva, Switzerland), human bronchial epithelial BEAS-2B cells (ATCC CRL-9609, gift from Pr Paul Hofman lab, IRCAN, Nice, France), human tumourigenic lung BZR cells obtained by transfer of v-Ha-ras oncogene into BEAS-2B (ATCC CRL-9483, gift from Pr Paul Hofman lab, IRCAN, Nice, France) obtained by transfer of v-Ha-ras oncogene into BEAS-2B cells, human lung epidermoid carcinoma Calu1 cells, human melanoma A375 cells (ATCC CRL-1619, gift from Pr Paul Hofman lab, IRCAN, Nice, France), human cervix cancer HeLa cells, human colon cancer HCT-116 and DLD-1 cells, lung adenocarcinoma LuCa62 cells derived from a patient (gift from Dr Véronique Serre-Beinier, CMU, Geneva, Switzerland).
Authentication	None of the cell lines used were authenticated
Mycoplasma contamination	All cell lines were tested negative for mycoplasma

Commonly misidentified lines (See ICLAC register) | No commonly misidentified cell lines were used

Palaeontology and Archaeology

Specimen provenance | *Provide provenance information for specimens and describe permits that were obtained for the work (including the name of the issuing authority, the date of issue, and any identifying information). Permits should encompass collection and, where applicable, export.*

Specimen deposition | *Indicate where the specimens have been deposited to permit free access by other researchers.*

Dating methods | *If new dates are provided, describe how they were obtained (e.g. collection, storage, sample pretreatment and measurement), where they were obtained (i.e. lab name), the calibration program and the protocol for quality assurance OR state that no new dates are provided.*

☐ Tick this box to confirm that the raw and calibrated dates are available in the paper or in Supplementary Information.

Ethics oversight | *Identify the organization(s) that approved or provided guidance on the study protocol, OR state that no ethical approval or guidance was required and explain why not.*

Note that full information on the approval of the study protocol must also be provided in the manuscript.

Animals and other research organisms

Policy information about studies involving animals; ARRIVE guidelines recommended for reporting animal research, and Sex and Gender in Research

Laboratory animals | 8 to 9 week-old female athymic nude-Foxn1nu mice mice were obtained from Envigen. Tumour cells were implanted in the mammary gland of eleven month-old animals.

Wild animals | No wild animals were used in this study

Reporting on sex | Sex was not considered in this study

Field-collected samples | No field-collected samples were used in this study.

Ethics oversight | For experiments performed at the University of Geneva: Institutional Animal Care and Use Committee of the University of Geneva and with permission of the Geneva cantonal authorities (Authorization number GE14420)
For experiments performed at TransCure, Archamps France: DAP: 2022082413416895

Note that full information on the approval of the study protocol must also be provided in the manuscript.

Clinical data

Policy information about clinical studies
All manuscripts should comply with the ICMJE guidelines for publication of clinical research and a completed CONSORT checklist must be included with all submissions.

Clinical trial registration | *Provide the trial registration number from ClinicalTrials.gov or an equivalent agency.*

Study protocol | *Note where the full trial protocol can be accessed OR if not available, explain why.*

Data collection | *Describe the settings and locales of data collection, noting the time periods of recruitment and data collection.*

Outcomes | *Describe how you pre-defined primary and secondary outcome measures and how you assessed these measures.*

Dual use research of concern

Policy information about dual use research of concern

Hazards

Could the accidental, deliberate or reckless misuse of agents or technologies generated in the work, or the application of information presented in the manuscript, pose a threat to:

No	Yes	
☐	☐	Public health
☐	☐	National security
☐	☐	Crops and/or livestock
☐	☐	Ecosystems
☐	☐	Any other significant area

Experiments of concern

Does the work involve any of these experiments of concern:

No	Yes	
☐	☐	Demonstrate how to render a vaccine ineffective
☐	☐	Confer resistance to therapeutically useful antibiotics or antiviral agents
☐	☐	Enhance the virulence of a pathogen or render a nonpathogen virulent
☐	☐	Increase transmissibility of a pathogen
☐	☐	Alter the host range of a pathogen
☐	☐	Enable evasion of diagnostic/detection modalities
☐	☐	Enable the weaponization of a biological agent or toxin
☐	☐	Any other potentially harmful combination of experiments and agents

Plants

Seed stocks	*Report on the source of all seed stocks or other plant material used. If applicable, state the seed stock centre and catalogue number. If plant specimens were collected from the field, describe the collection location, date and sampling procedures.*
Novel plant genotypes	*Describe the methods by which all novel plant genotypes were produced. This includes those generated by transgenic approaches, gene editing, chemical/radiation-based mutagenesis and hybridization. For transgenic lines, describe the transformation method, the number of independent lines analyzed and the generation upon which experiments were performed. For gene-edited lines, describe the editor used, the endogenous sequence targeted for editing, the targeting guide RNA sequence (if applicable) and how the editor was applied.*
Authentication	*Describe any authentication procedures for each seed stock used or novel genotype generated. Describe any experiments used to assess the effect of a mutation and, where applicable, how potential secondary effects (e.g. second site T-DNA insertions, mosiacism, off-target gene editing) were examined.*

ChIP-seq

Data deposition

☐ Confirm that both raw and final processed data have been deposited in a public database such as GEO.

☐ Confirm that you have deposited or provided access to graph files (e.g. BED files) for the called peaks.

Data access links<br>*May remain private before publication.*	*For "Initial submission" or "Revised version" documents, provide reviewer access links. For your "Final submission" document, provide a link to the deposited data.*
Files in database submission	*Provide a list of all files available in the database submission.*
Genome browser session<br>(e.g. UCSC)	*Provide a link to an anonymized genome browser session for "Initial submission" and "Revised version" documents only, to enable peer review. Write "no longer applicable" for "Final submission" documents.*

Methodology

Replicates	*Describe the experimental replicates, specifying number, type and replicate agreement.*
Sequencing depth	*Describe the sequencing depth for each experiment, providing the total number of reads, uniquely mapped reads, length of reads and whether they were paired- or single-end.*
Antibodies	*Describe the antibodies used for the ChIP-seq experiments; as applicable, provide supplier name, catalog number, clone name, and lot number.*
Peak calling parameters	*Specify the command line program and parameters used for read mapping and peak calling, including the ChIP, control and index files used.*

Data quality	*Describe the methods used to ensure data quality in full detail, including how many peaks are at FDR 5% and above 5-fold enrichment.*
Software	*Describe the software used to collect and analyze the ChIP-seq data. For custom code that has been deposited into a community repository, provide accession details.*

Flow Cytometry

Plots

Confirm that:

☐ The axis labels state the marker and fluorochrome used (e.g. CD4-FITC).

☐ The axis scales are clearly visible. Include numbers along axes only for bottom left plot of group (a 'group' is an analysis of identical markers).

☐ All plots are contour plots with outliers or pseudocolor plots.

☐ A numerical value for number of cells or percentage (with statistics) is provided.

Methodology

Sample preparation	*Describe the sample preparation, detailing the biological source of the cells and any tissue processing steps used.*
Instrument	*Identify the instrument used for data collection, specifying make and model number.*
Software	*Describe the software used to collect and analyze the flow cytometry data. For custom code that has been deposited into a community repository, provide accession details.*
Cell population abundance	*Describe the abundance of the relevant cell populations within post-sort fractions, providing details on the purity of the samples and how it was determined.*
Gating strategy	*Describe the gating strategy used for all relevant experiments, specifying the preliminary FSC/SSC gates of the starting cell population, indicating where boundaries between "positive" and "negative" staining cell populations are defined.*

☐ Tick this box to confirm that a figure exemplifying the gating strategy is provided in the Supplementary Information.

Magnetic resonance imaging

Experimental design

Design type	*Indicate task or resting state; event-related or block design.*
Design specifications	*Specify the number of blocks, trials or experimental units per session and/or subject, and specify the length of each trial or block (if trials are blocked) and interval between trials.*
Behavioral performance measures	*State number and/or type of variables recorded (e.g. correct button press, response time) and what statistics were used to establish that the subjects were performing the task as expected (e.g. mean, range, and/or standard deviation across subjects).*

Acquisition

Imaging type(s)	*Specify: functional, structural, diffusion, perfusion.*
Field strength	*Specify in Tesla*
Sequence & imaging parameters	*Specify the pulse sequence type (gradient echo, spin echo, etc.), imaging type (EPI, spiral, etc.), field of view, matrix size, slice thickness, orientation and TE/TR/flip angle.*
Area of acquisition	*State whether a whole brain scan was used OR define the area of acquisition, describing how the region was determined.*

Diffusion MRI ☐ Used ☐ Not used

Preprocessing

Preprocessing software	*Provide detail on software version and revision number and on specific parameters (model/functions, brain extraction, segmentation, smoothing kernel size, etc.).*
Normalization	*If data were normalized/standardized, describe the approach(es): specify linear or non-linear and define image types used for transformation OR indicate that data were not normalized and explain rationale for lack of normalization.*

Normalization template | *Describe the template used for normalization/transformation, specifying subject space or group standardized space (e.g. original Talairach, MNI305, ICBM152) OR indicate that the data were not normalized.*

Noise and artifact removal | *Describe your procedure(s) for artifact and structured noise removal, specifying motion parameters, tissue signals and physiological signals (heart rate, respiration).*

Volume censoring | *Define your software and/or method and criteria for volume censoring, and state the extent of such censoring.*

Statistical modeling & inference

Model type and settings | *Specify type (mass univariate, multivariate, RSA, predictive, etc.) and describe essential details of the model at the first and second levels (e.g. fixed, random or mixed effects; drift or auto-correlation).*

Effect(s) tested | *Define precise effect in terms of the task or stimulus conditions instead of psychological concepts and indicate whether ANOVA or factorial designs were used.*

Specify type of analysis: ☐ Whole brain ☐ ROI-based ☐ Both

Statistic type for inference | *Specify voxel-wise or cluster-wise and report all relevant parameters for cluster-wise methods.*

(See Eklund et al. 2016)

Correction | *Describe the type of correction and how it is obtained for multiple comparisons (e.g. FWE, FDR, permutation or Monte Carlo).*

Models & analysis

n/a	Involved in the study
☐	☐ Functional and/or effective connectivity
☐	☐ Graph analysis
☐	☐ Multivariate modeling or predictive analysis

Functional and/or effective connectivity | *Report the measures of dependence used and the model details (e.g. Pearson correlation, partial correlation, mutual information).*

Graph analysis | *Report the dependent variable and connectivity measure, specifying weighted graph or binarized graph, subject- or group-level, and the global and/or node summaries used (e.g. clustering coefficient, efficiency, etc.).*

Multivariate modeling and predictive analysis | *Specify independent variables, features extraction and dimension reduction, model, training and evaluation metrics.*
