# Supplementary material for: d-cysteine impairs tumour growth by inhibiting cysteine desulfurase NFS1
Source: Nat Metab. 2025 Aug 12;7(8):1646–62. doi: 10.1038/s42255-025-01339-1 (PMC12373508; doi:10.1038/s42255-025-01339-1)
Supplement: Supplementary file 11 — Unprocessed western Blots. [file 42255_2025_1339_MOESM11_ESM.pdf]

SourceData_Fig. 3abch

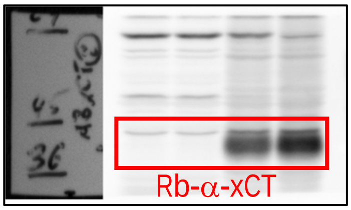

AB_xCT#07-11-22_Round5(20sec),8-8. scan_raw

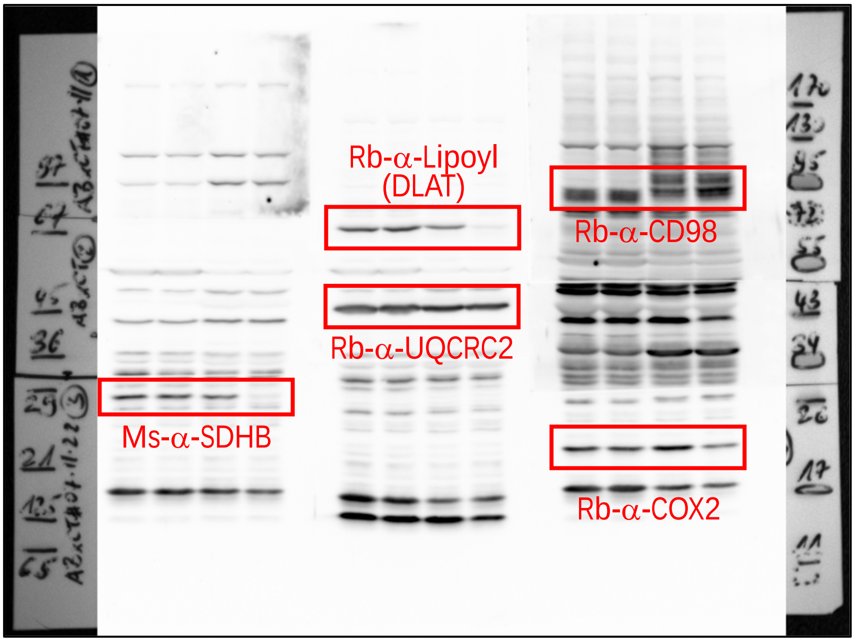

AB_xCT#07-11-22_Round1,3-3. scan_raw

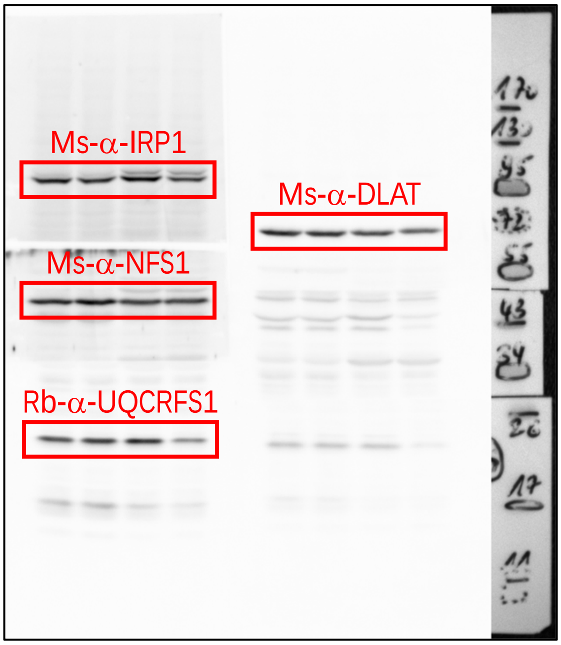

AB_xCT#07-11-22_Round2,1-1. scan_raw

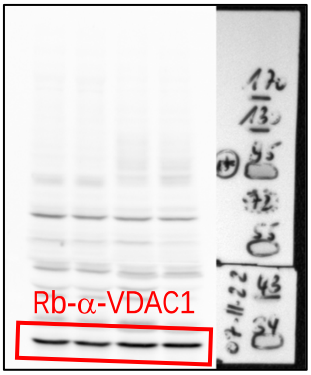

AB_xCT#07-11-22_Round5
(20sec),5-5. scan_raw

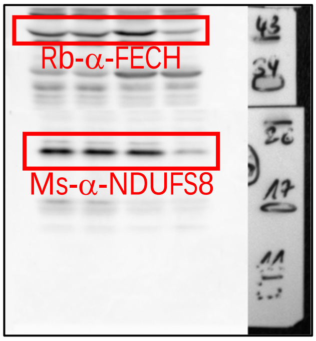

AB_xCT#07-11-22_Round2,
12-12. scan_raw

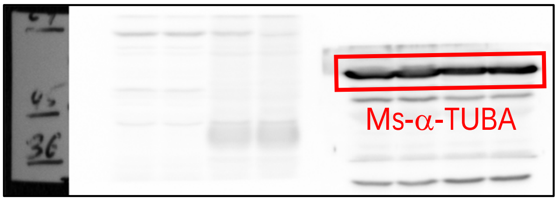

AB_xCT#07-11-22_Round5(10sec),4-4. scan_raw

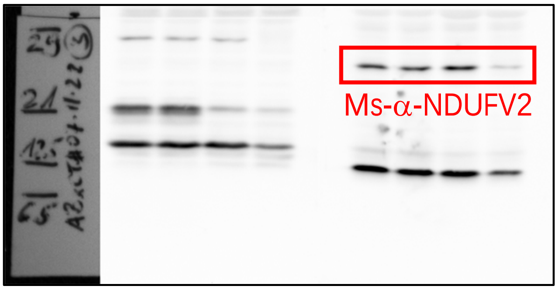

AB_xCT#07-11-22_Round3,5-5. scan_raw

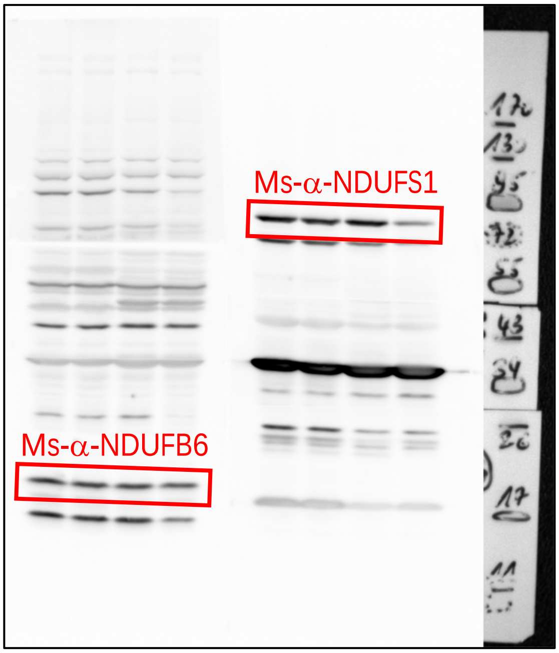

AB_xCT#07-11-22_Round4,4-4. scan_raw

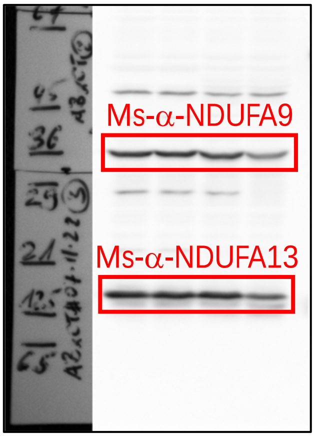

AB_xCT#07-11-22_Round2,
3-3. scan_raw

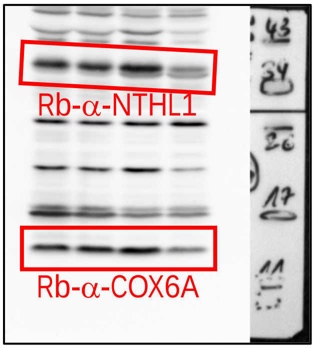

AB_xCT#07-11-22_Round3,
12-12. scan_raw

SourceData_Fig. 3abch

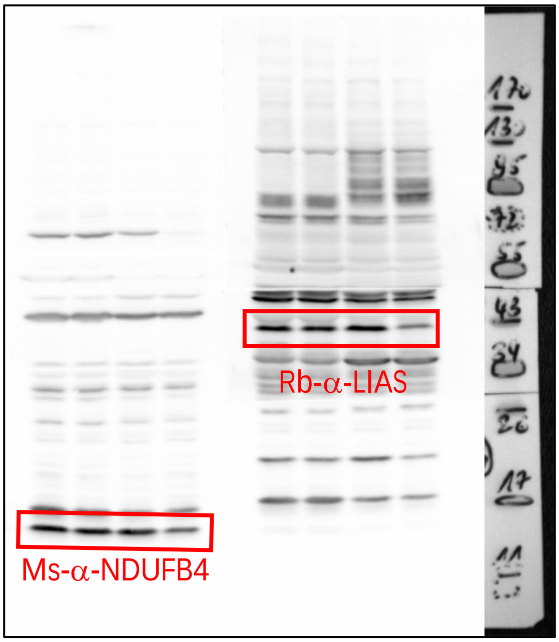

AB_xCT#07-11-22_Round1,1-1. scan_raw

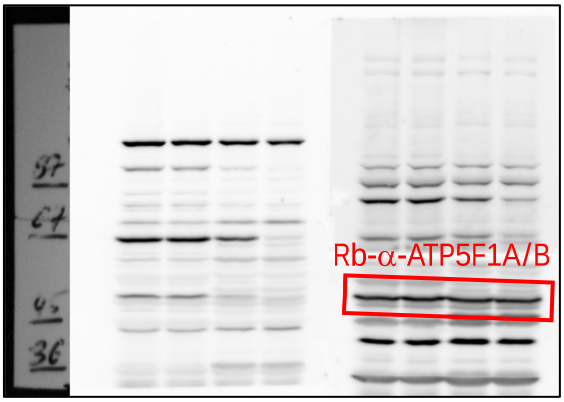

AB_xCT#07-11-22_Round4,10-10. scan_raw

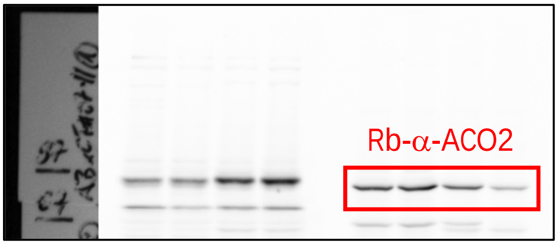

AB_xCT#07-11-22_Round3,3-3. scan_raw

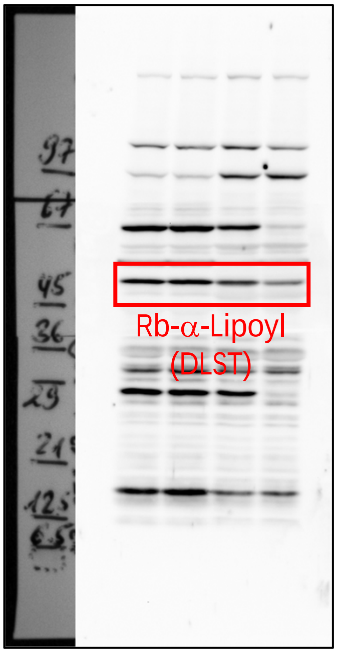

AB_xCT#17-10-22_Round1,
14-14. scan_raw

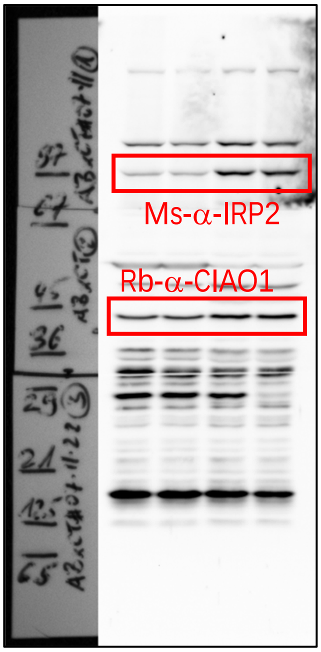

AB_xCT#07-11-22_Round1,
8-8. scan_raw

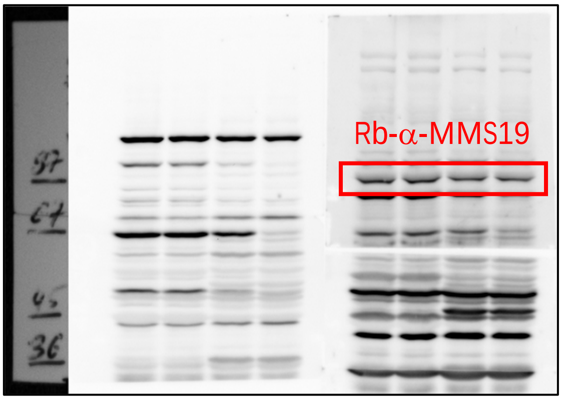

AB_xCT#07-11-22_Round4,16-16. scan_raw

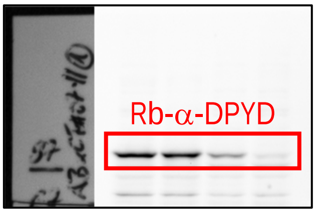

AB_xCT#07-11-22_Round2,
8-8. scan_raw

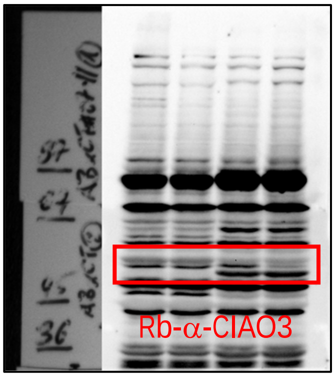

AB_xCT#07-11-22_Round3,
22-22. scan_raw

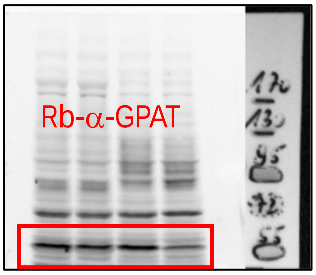

AB_xCT#07-11-22_Round3,
22-22. scan_raw

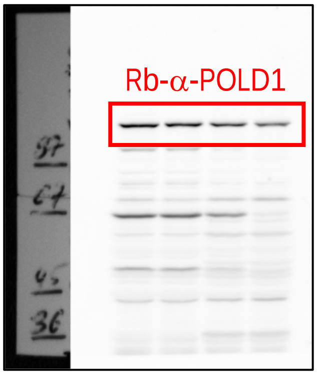

AB_xCT#07-11-22_Round4,
5-5. scan_raw
