# Supplementary figures and images for: d-cysteine impairs tumour growth by inhibiting cysteine desulfurase NFS1
Source: Nat Metab. 2025 Aug 12;7(8):1646–62. doi: 10.1038/s42255-025-01339-1 (PMC12373508; doi:10.1038/s42255-025-01339-1)

Extended Data Figure 2b

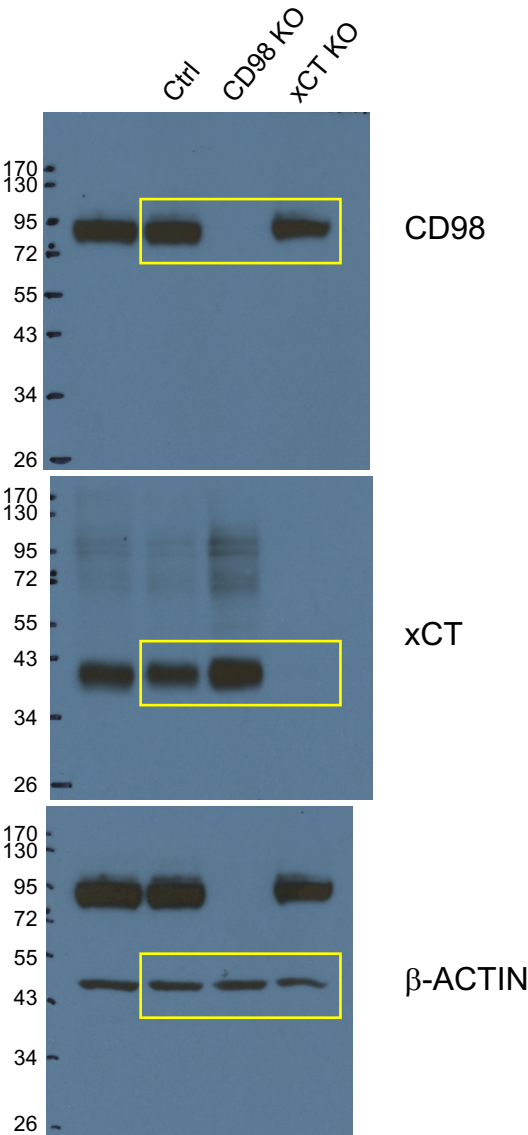

Extended Data Figure 2c

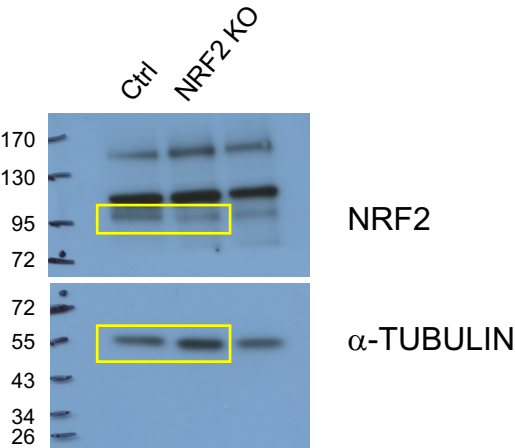

Supplement: Supplementary file 16 — Unprocessed western blots. [file 42255_2025_1339_MOESM16_ESM.pdf]

Extended Data Figure 3a

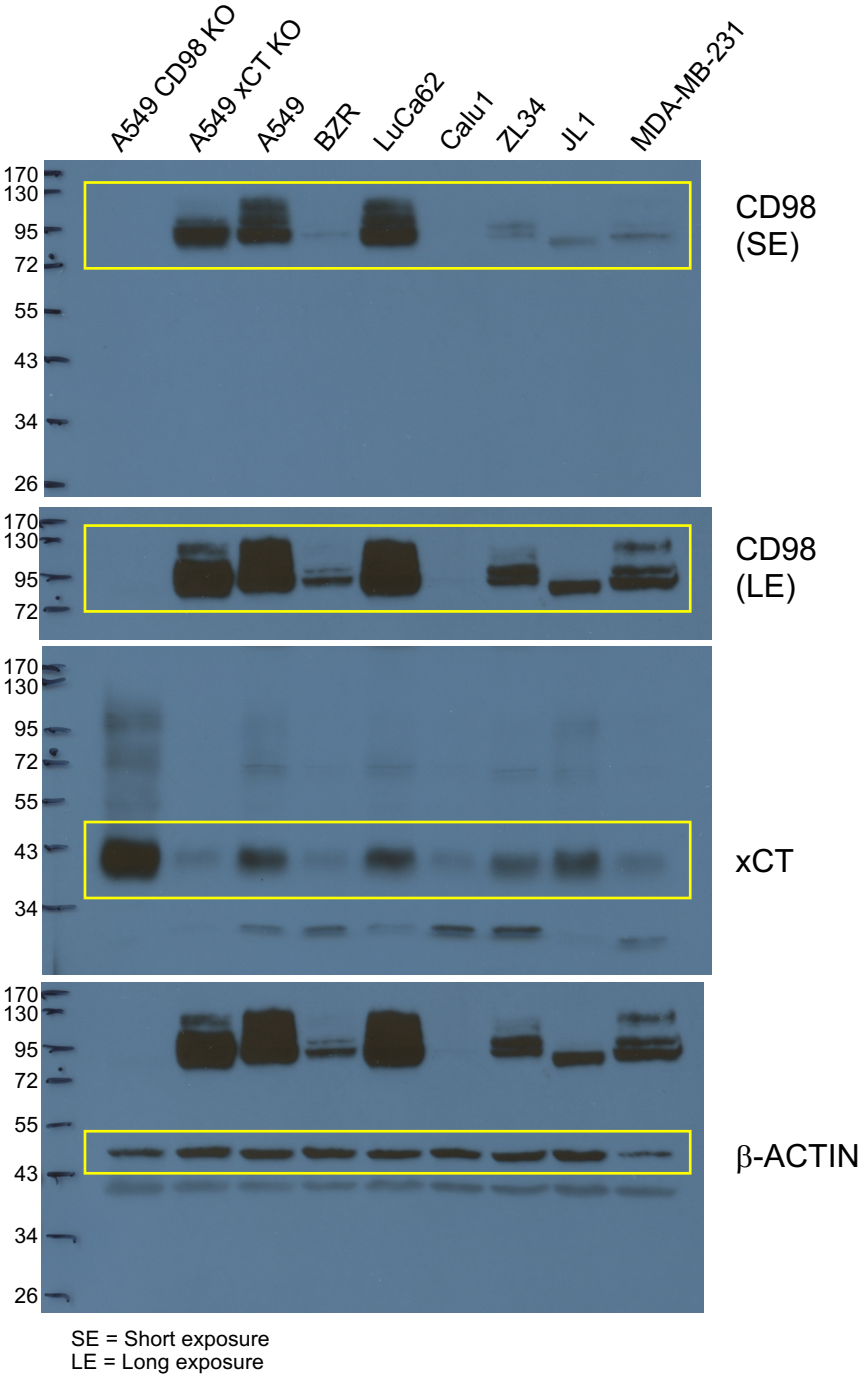

Extended Data Figure 3b

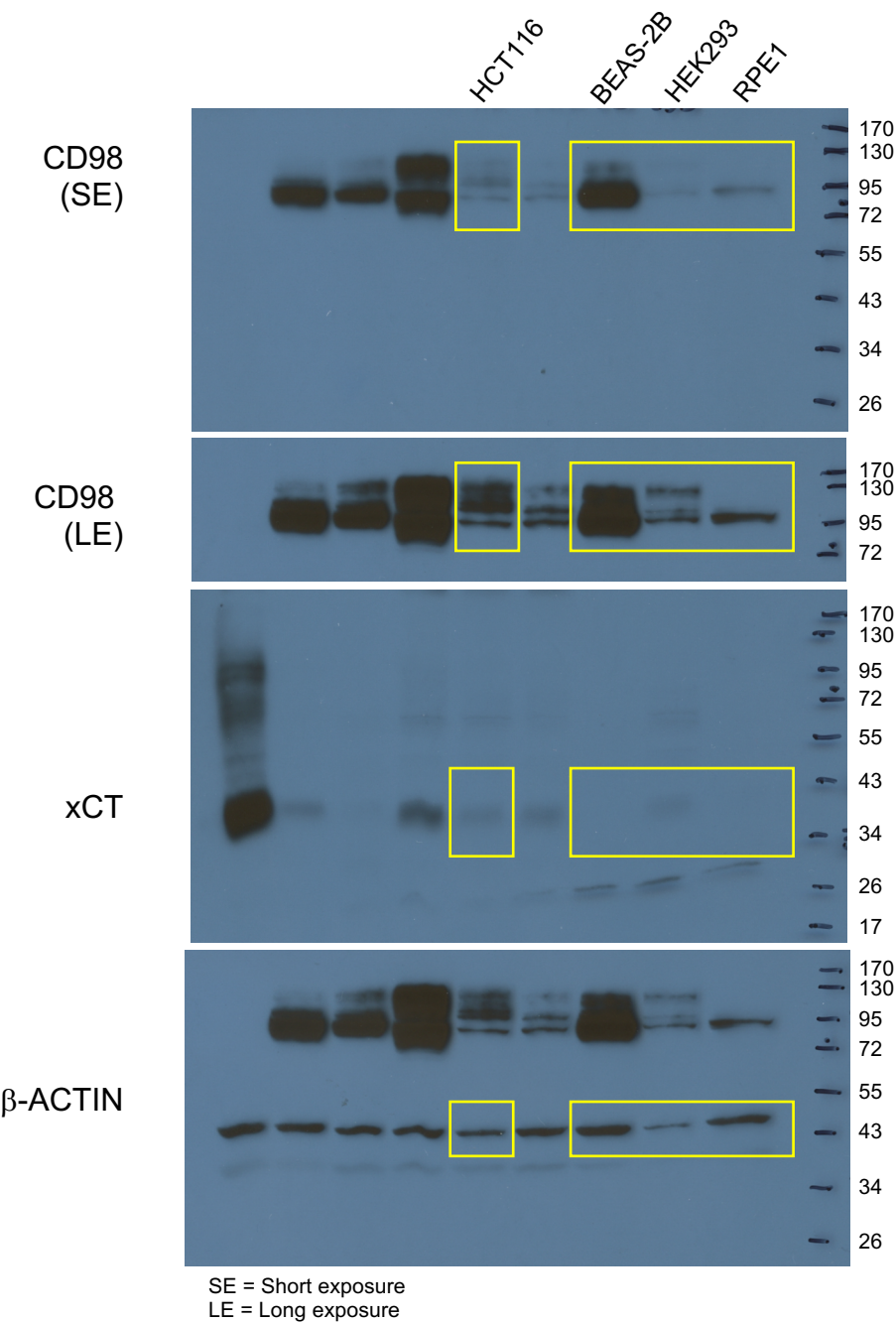

Extended Data Figure 3c

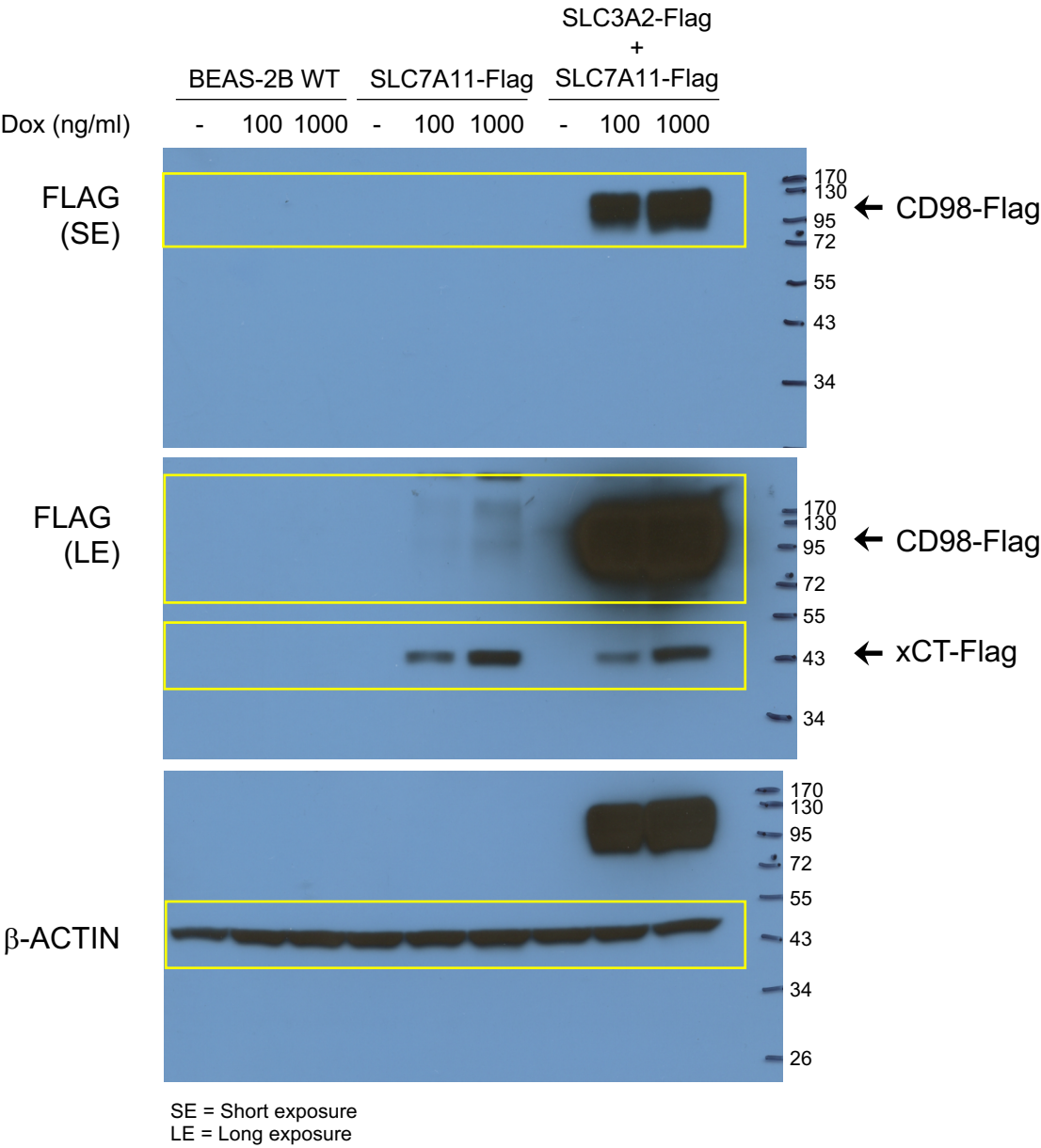

Supplement: Supplementary file 18 — Unprocessed western blots. [file 42255_2025_1339_MOESM18_ESM.pdf]

Extended Data Figure 4c

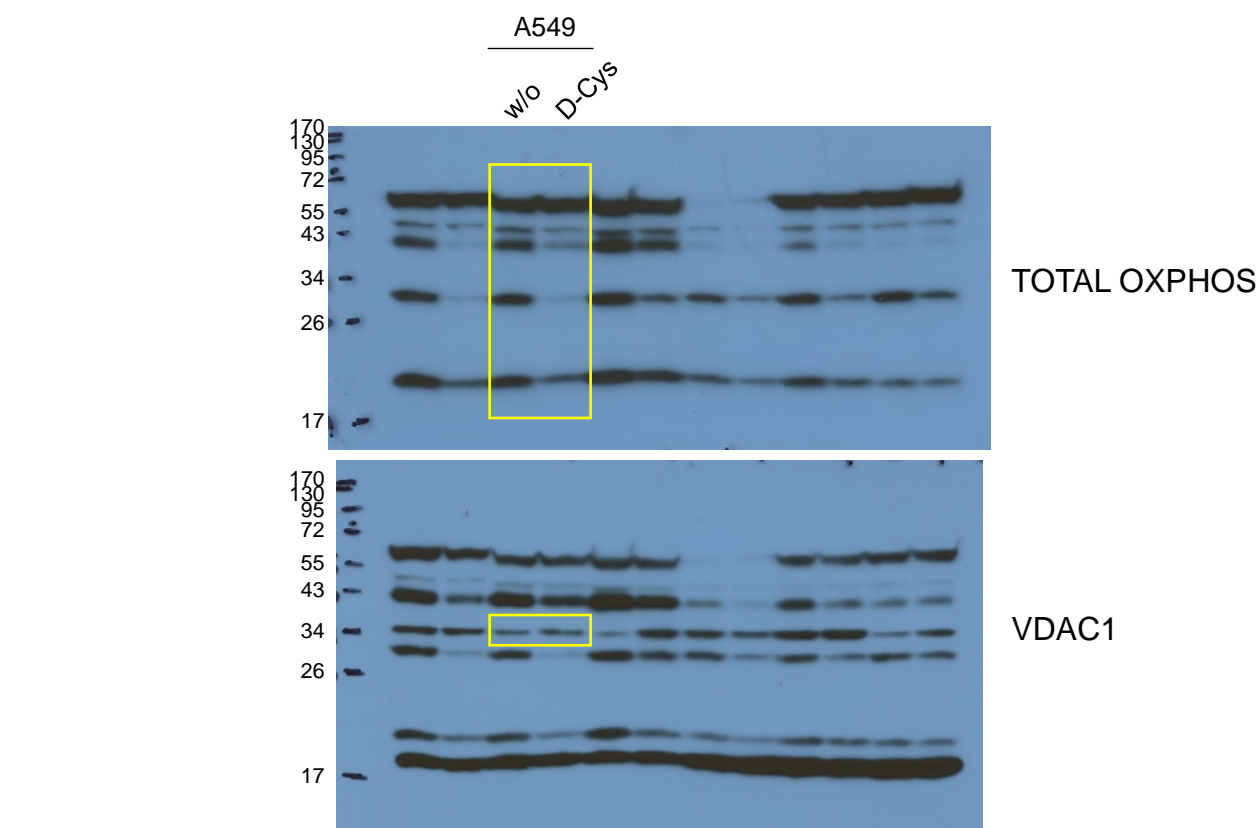

Extended Data Figure 4c

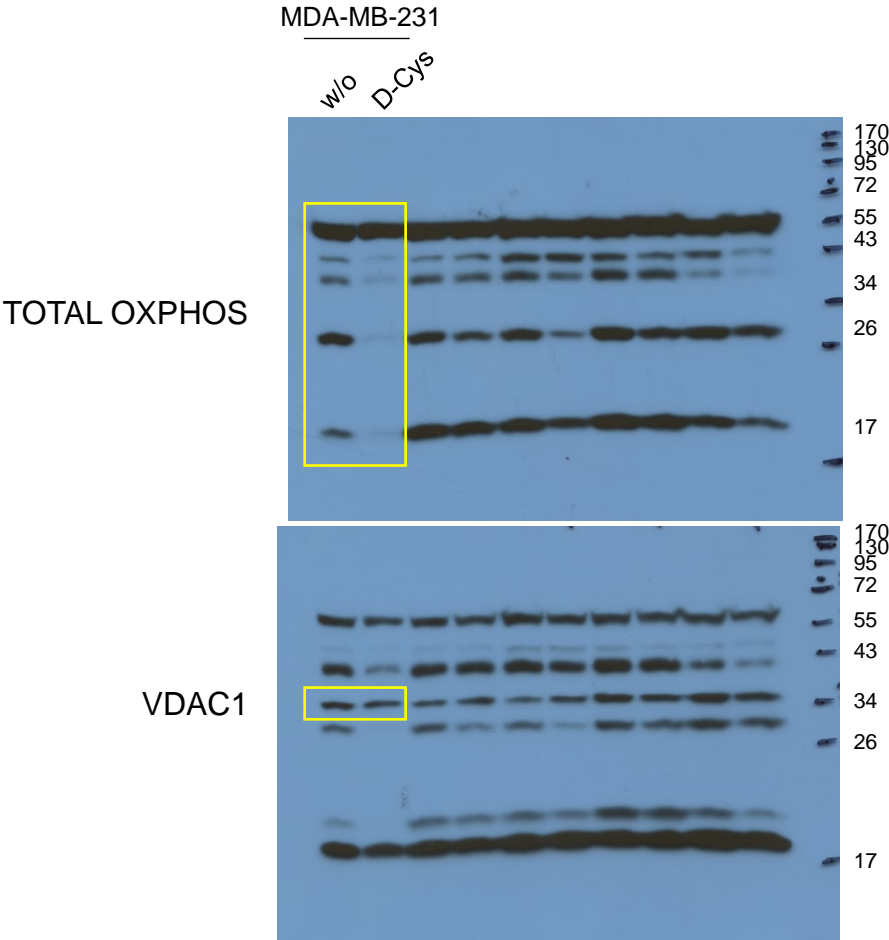

Extended Data Figure 4c

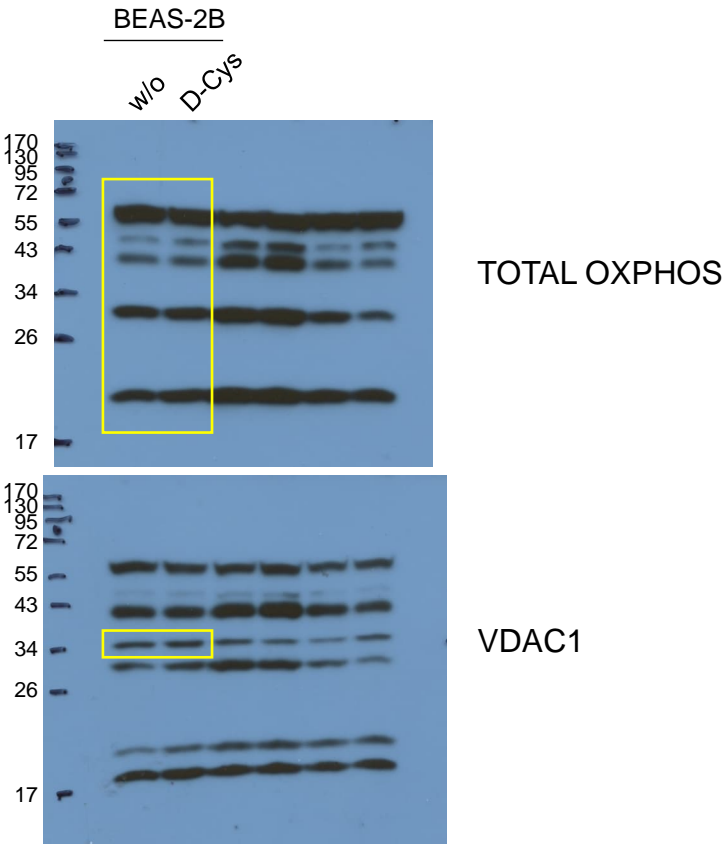

Supplement: Supplementary file 20 — Unprocessed western blots. [file 42255_2025_1339_MOESM20_ESM.pdf]
