# Supplementary material for: d-cysteine impairs tumour growth by inhibiting cysteine desulfurase NFS1
Source: Nat Metab. 2025 Aug 12;7(8):1646–62. doi: 10.1038/s42255-025-01339-1 (PMC12373508; doi:10.1038/s42255-025-01339-1)
Supplement: Supplementary file 21 — Original flow cytometry data. [file 42255_2025_1339_MOESM21_ESM.pdf]

a

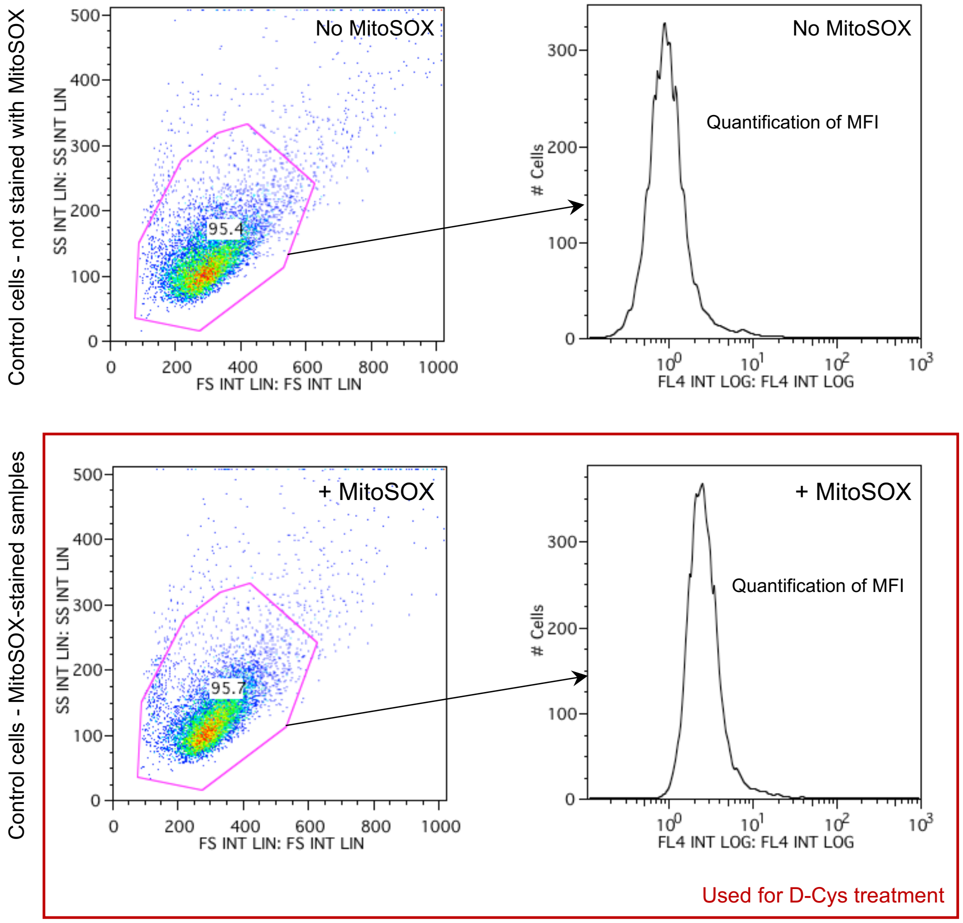
Control cells - not stained with MitoSOX
No MitoSOX
SS INT LIN: SS INT LIN
FS INT LIN: FS INT LIN
95.4
Cells
Quantification of MFI
FL4 INT LOG: FL4 INT LOG
Control cells - MitoSOX-stained samples
+ MitoSOX
95.7
Used for D-Cys treatment

b

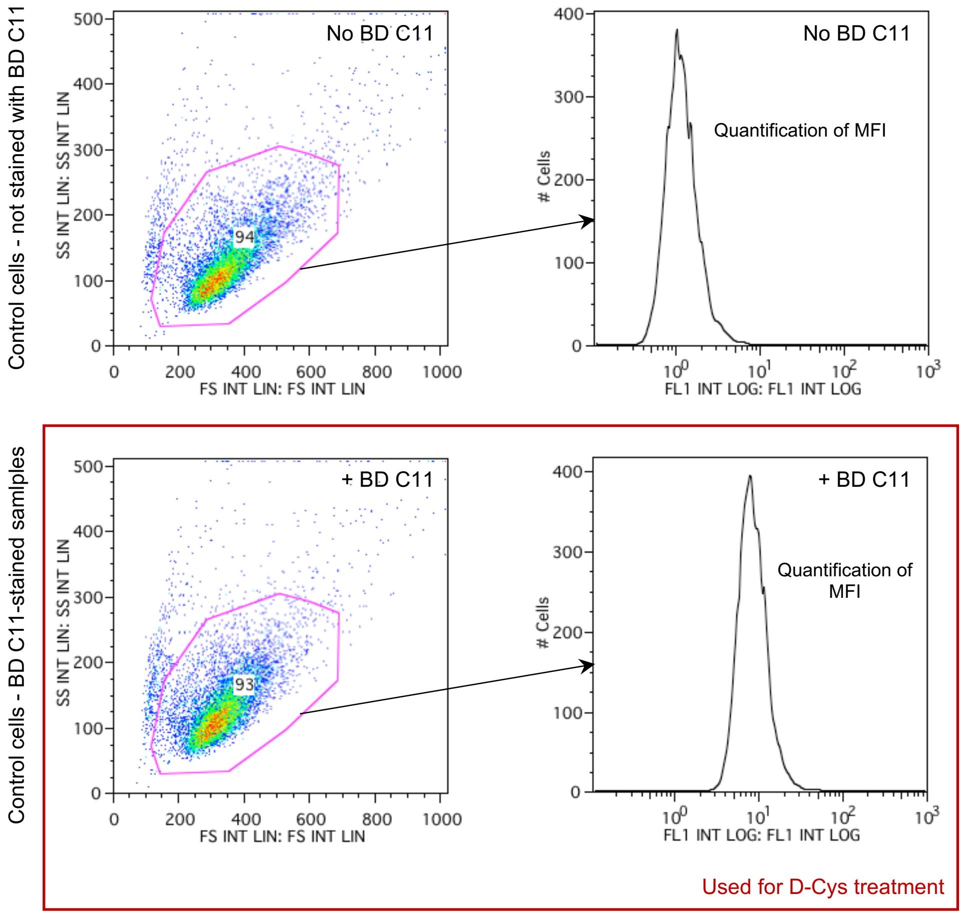
Control cells - not stained with BD C11
No BD C11
SS INT LIN: SS INT LIN
FS INT LIN: FS INT LIN
94
Cells
Quantification of MFI
FL1 INT LOG: FL1 INT LOG
Control cells - BD C11-stained samples
+ BD C11
93
Quantification of MFI
Used for D-Cys treatment
