# Supplementary material for: d-cysteine impairs tumour growth by inhibiting cysteine desulfurase NFS1
Source: Nat Metab. 2025 Aug 12;7(8):1646–62. doi: 10.1038/s42255-025-01339-1 (PMC12373508; doi:10.1038/s42255-025-01339-1)
Supplement: Supplementary file 23 — Unprocessed western blots. [file 42255_2025_1339_MOESM23_ESM.pdf]

SourceData_ExtFig6abg

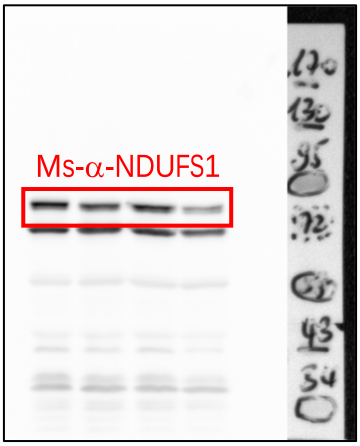

HeLa_xCT#29-08-22_Round3, 2-2. scan_raw

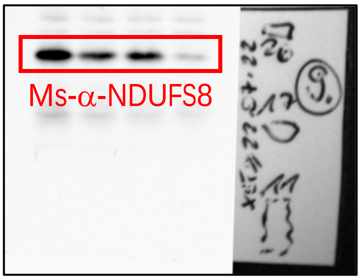

HeLa_xCT#22-07-22_Round2, 7-7. scan_raw

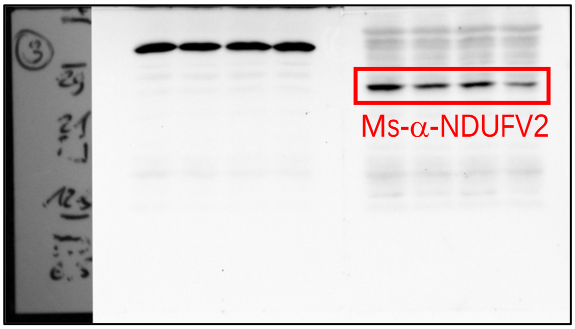

HeLa_xCT#22-07-22_Round3,7-7. scan_raw

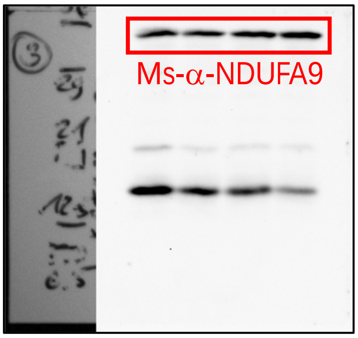

HeLa_xCT#22-07-22_Round5, 7-7. scan_raw

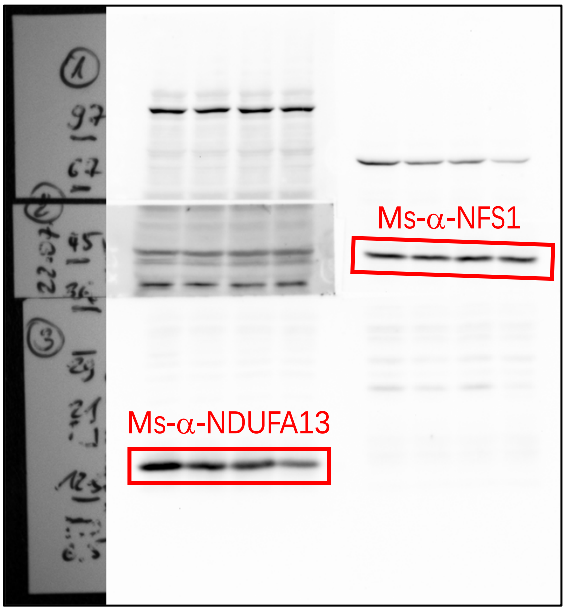

HeLa_xCT#22-07-22_Round2,2-2. scan_raw

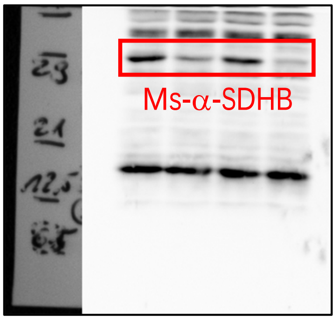

HeLa_xCT#29-08-22_ Round1(1min),19-19. scan_raw

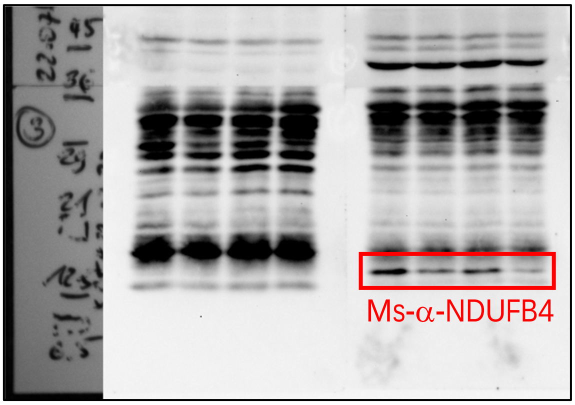

HeLa_xCT#22-07-22_Round1,7-7. scan_raw

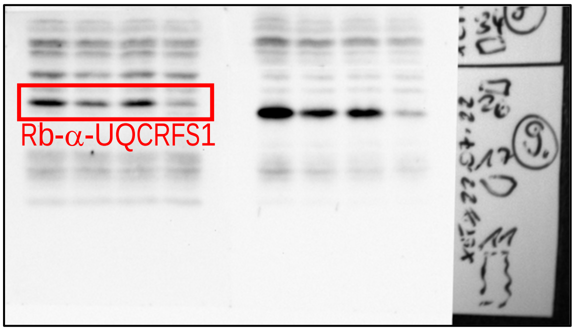

HeLa_xCT#22-07-22_Round2,7-7. scan_raw

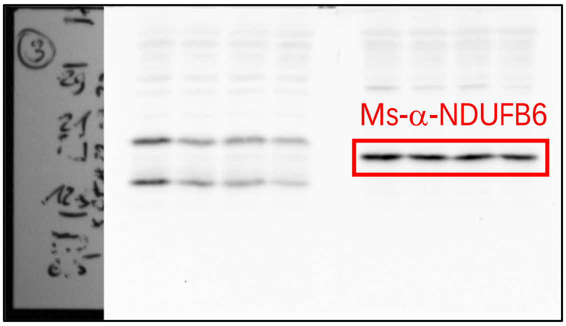

HeLa_xCT#22-07-22_Round4,7-7. scan_raw

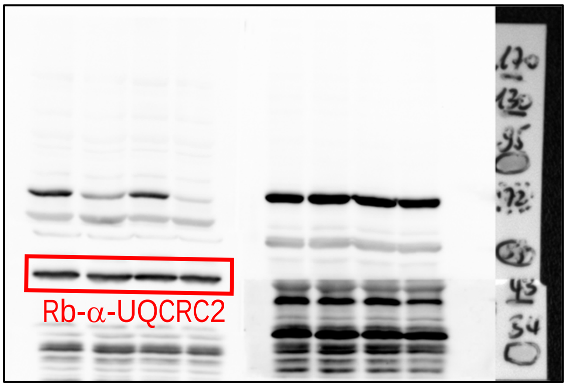

HeLa_xCT#29-08-22_Round1(1min),5-5. scan_raw

# SourceData_ExtFig6abg

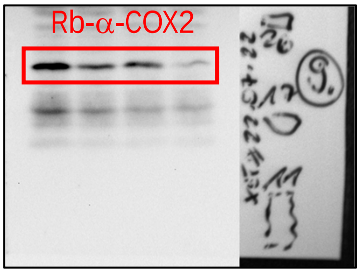


HeLa_xCT#22-07-22_Round1, 7-7. scan_raw

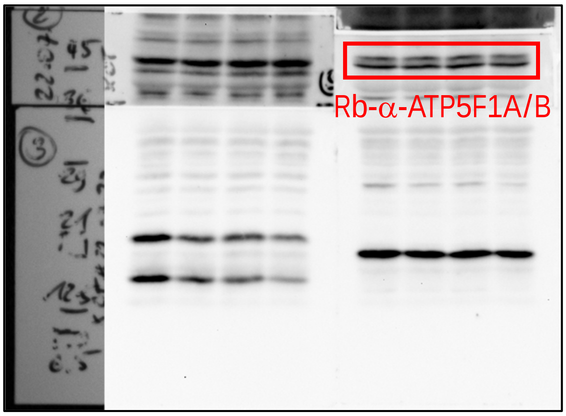


HeLa_xCT#22-07-22_Round4,7-7. scan_raw

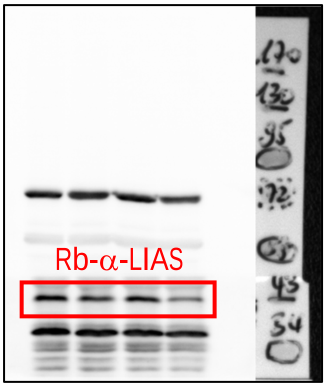


HeLa_xCT#29-08-22_ Round1(1min),1-1. scan_raw

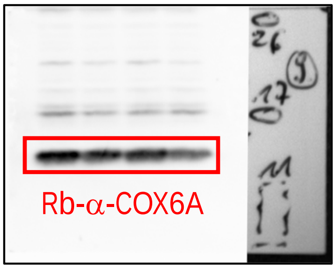


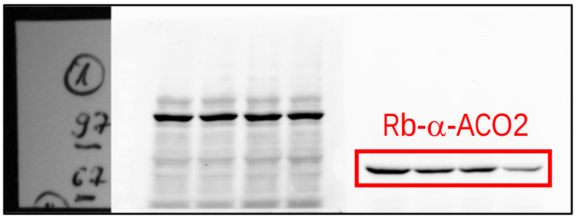


HeLa_xCT#22-07-22_Round2,7-7. scan_raw

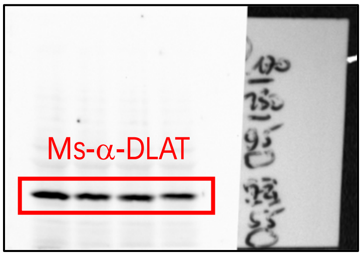


HeLa_xCT#22-07-22_Round2, 7-7. scan_raw

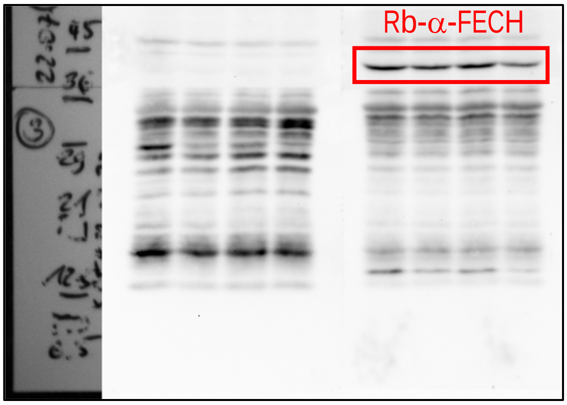


HeLa_xCT#22-07-22_Round1,7-7. scan_raw

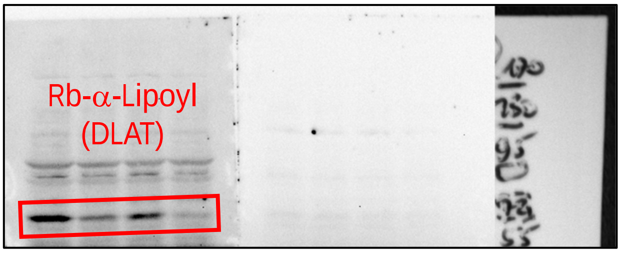


HeLa_xCT#22-07-22_Round1,7-7. scan_raw

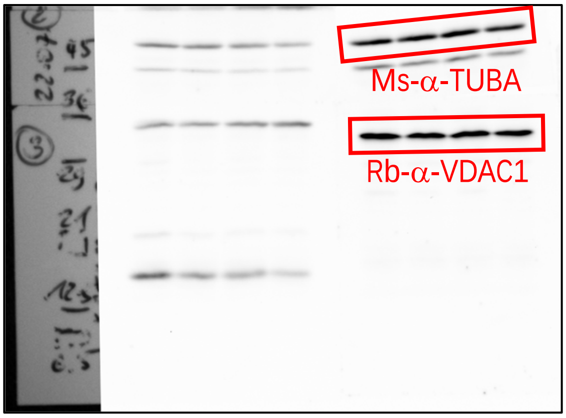


HeLa_xCT#22-07-22_Round5,4-4. scan_raw

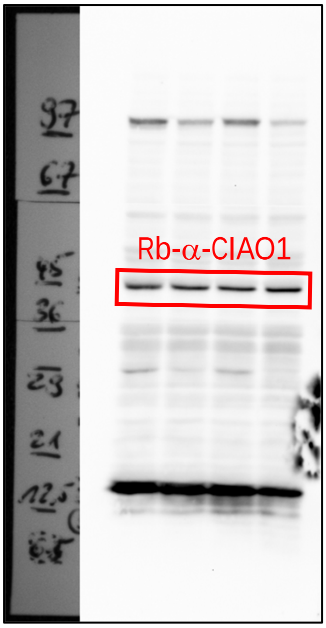


HeLa_xCT#29-08-22_Round2, 9-9. scan_raw

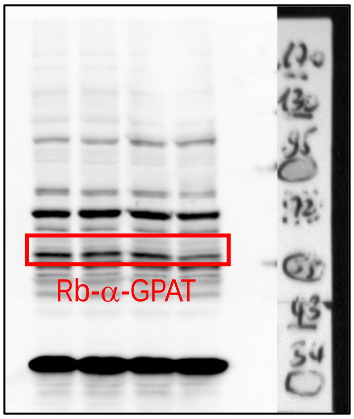


HeLa_xCT#29-08-22_Round4(1min), 5-5. scan_raw

# SourceData_ExtFig6abg

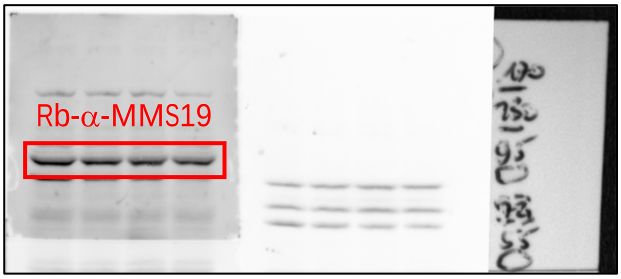


HeLa_xCT#22-07-22_Round3,7-7. scan_raw

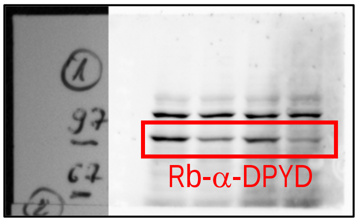


HeLa_xCT#22-07-22_Round5, 7-7. scan_raw

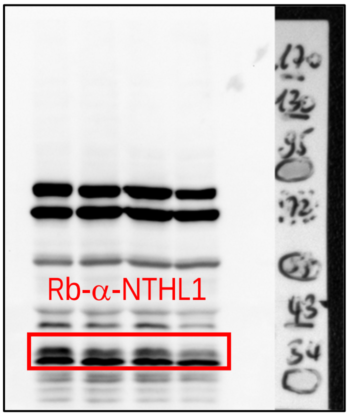


HeLa_xCT#29-08-22_Round3, 20-20. scan_raw

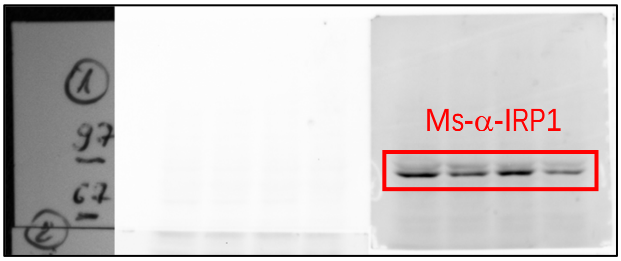


HeLa_xCT#22-07-22_Round4,3-3. scan_raw

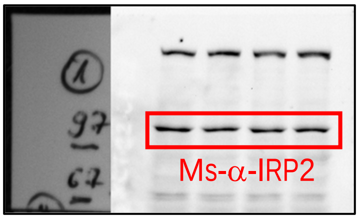


HeLa_xCT#22-07-22_Round1, 7-7. scan_raw
