# Supplementary material for: d-cysteine impairs tumour growth by inhibiting cysteine desulfurase NFS1
Source: Nat Metab. 2025 Aug 12;7(8):1646–62. doi: 10.1038/s42255-025-01339-1 (PMC12373508; doi:10.1038/s42255-025-01339-1)
Supplement: Supplementary file 25 — Unprocessed western blots. [file 42255_2025_1339_MOESM25_ESM.pdf]

SourceData_ExtFig9eghk_
left(D-Cys)

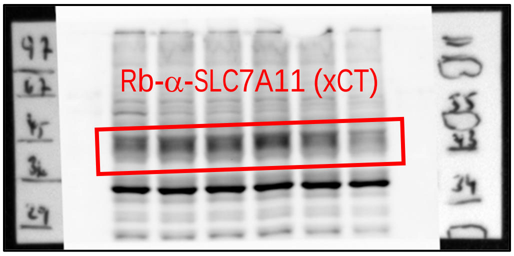

A549_xCT#30-01-23ab_Tricine_Round2[r],
15-15. scan_raw

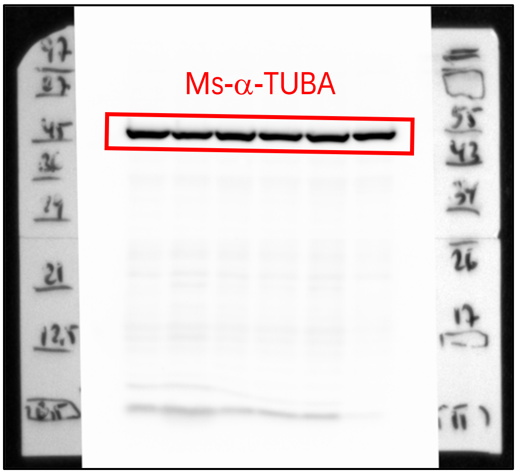

A549_xCT#06-02-23a_Tricine_
round3,2-2. scan_raw

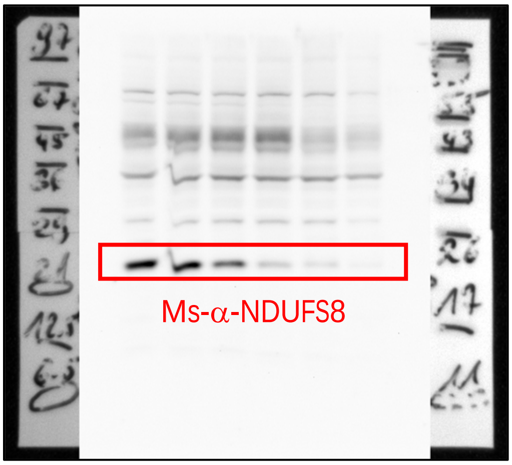

A549_xCT#28-11-22a_Round2,
15-15. scan_raw

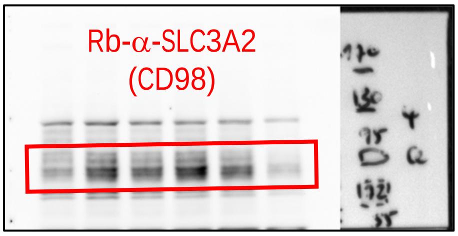

A549_xCT#30-01-23a_Gradient_Round1,
9-9. scan_raw

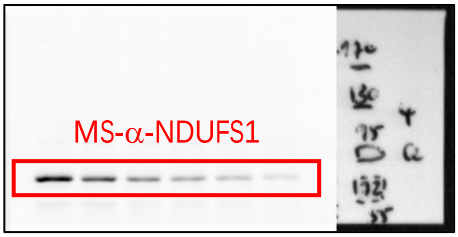

A549_xCT#30-01-23a_Gradient_Round2,
3-3. scan_raw

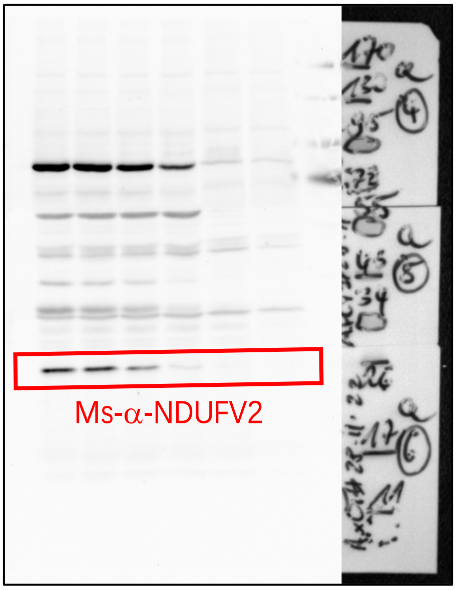

A549_xCT#28-11-22a_Gradient_
Round2,15-15. scan_raw

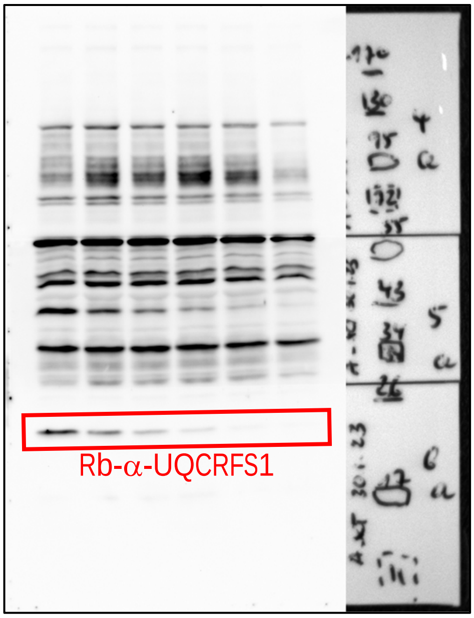

A549_xCT#30-01-23a_Gradient_Round1,
12-12. scan_raw

SourceData_ExtFig9eghk_left(D-Cys)

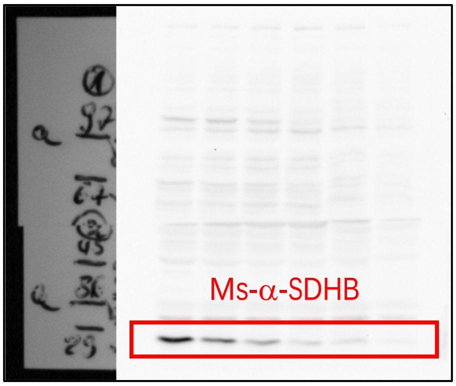

A549_xCT#28-11-22a_Gradient_
Round3,15-15. scan_raw

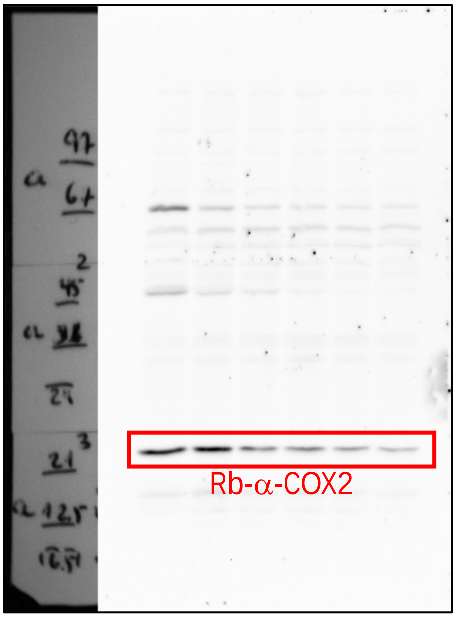

A549_xCT#30-01-23a_Gradient_Round1,
13-13. scan_raw

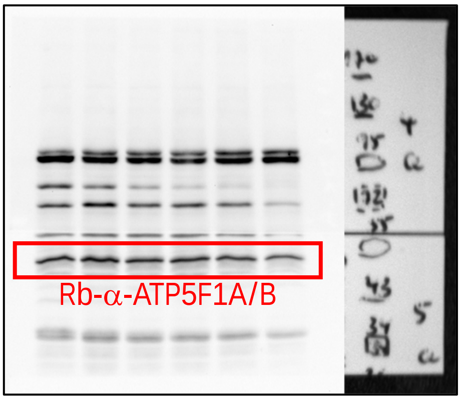

A549_xCT#30-01-23a_Gradient_Round4,
15-15. scan_raw

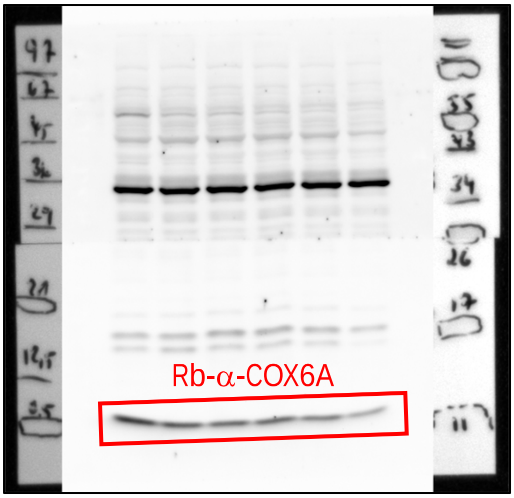

A549_xCT#30-01-23a_Tricine_Round1,
7-7. scan_raw

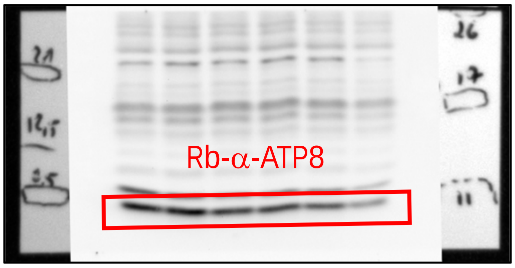

A549_xCT#30-01-23a_Tricine_Round3,
20-20. scan_raw

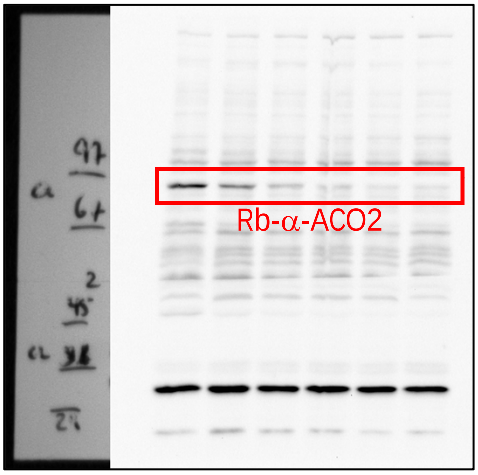

A549_xCT#30-01-23a_Gradient_Round4,
15-15. scan_raw

SourceData_ExtFig9eghk_
left(D-Cys)

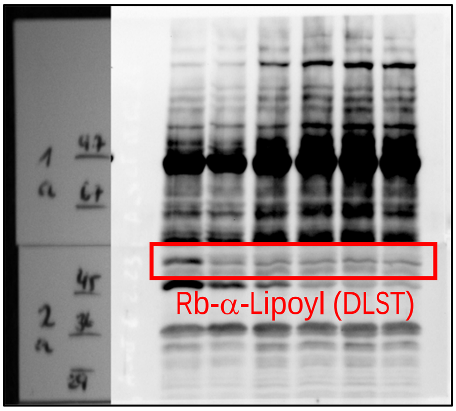

A549_xCT#06-02-23a_Gradient_
Round2,15-15. scan_raw

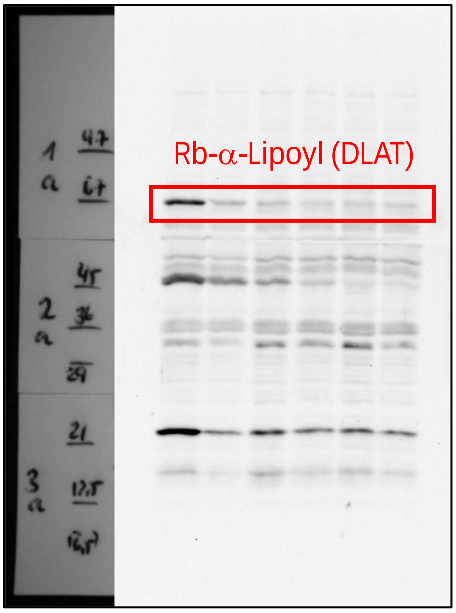

A549_xCT#06-02-23a_Gradient_
Round1,15-15. scan_raw

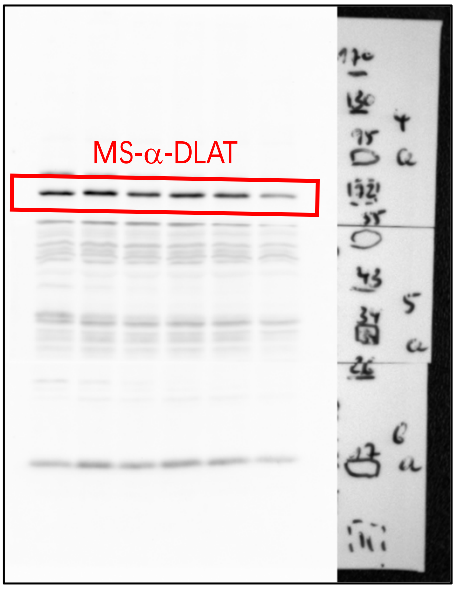

A549_xCT#30-01-23a_Gradient_Round3,
7-7. scan_raw

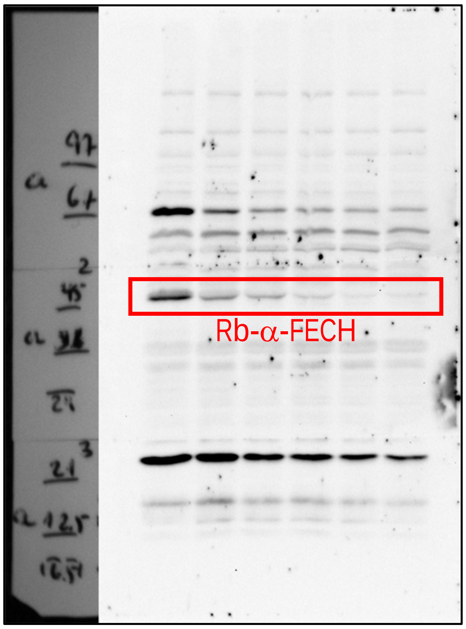

A549_xCT#30-01-23a_Gradient_Round1,
15-15. scan_raw

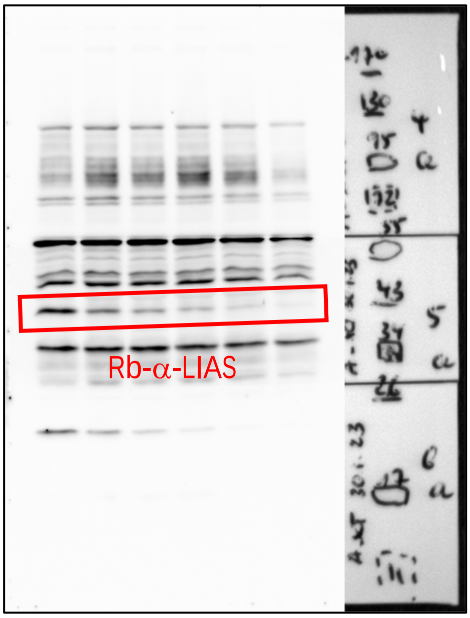

A549_xCT#30-01-23a_Gradient_Round1,
7-7. scan_raw

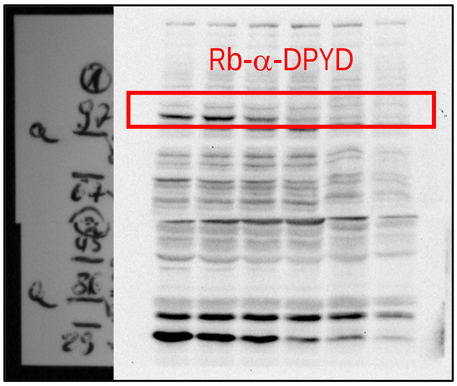

A549_xCT#28-11-22a_Gradient_
Round3,15-15. scan_raw

SourceData_ExtFig9eghk_left(D-Cys)

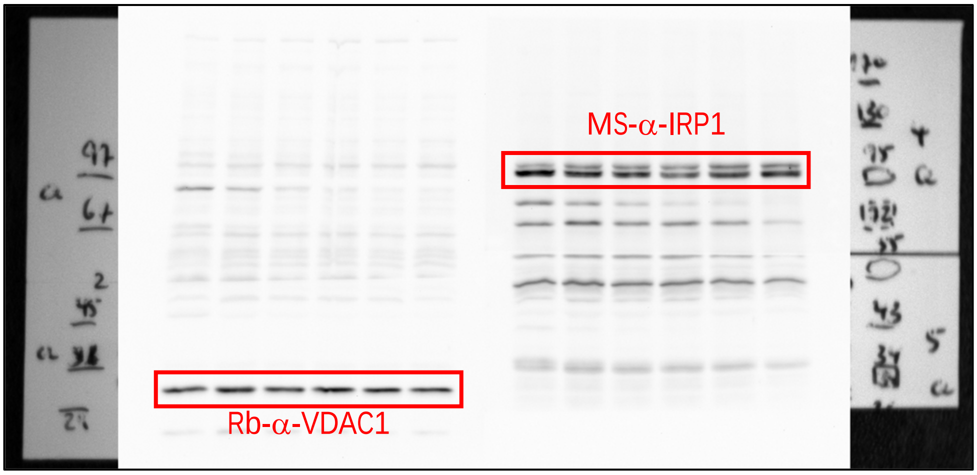

A549_xCT#30-01-23a_Gradient_Round4,15-15. scan_raw

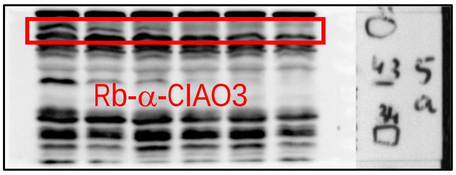

A549_xCT#06-02-23a_Gradient_
Round3,15-15. scan_raw

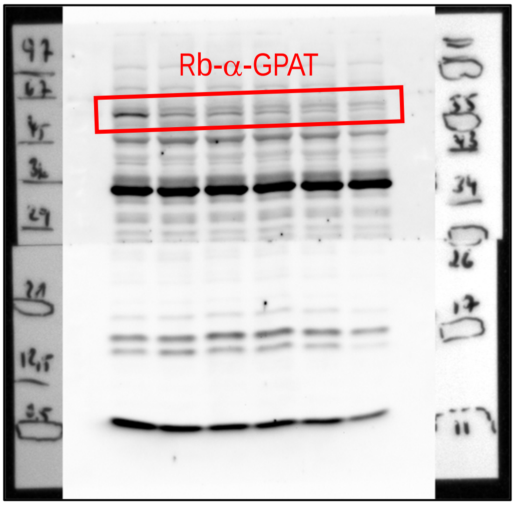

A549_xCT#30-01-23a_Tricine_Round1,
15-15. scan_raw

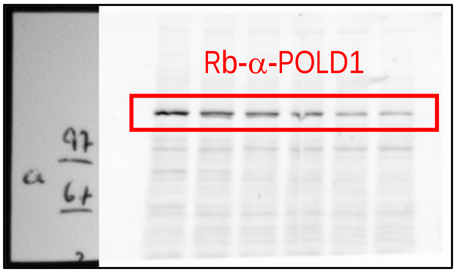

A549_xCT#30-01-23ab_Gradient_
Round5(WDH),3-3. scan_raw

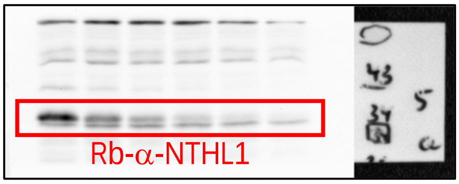

A549_xCT#30-01-23ab_Gradient_Round2[r],
15-15. scan_raw

SourceData_ExtFig9eghk_right(wo)

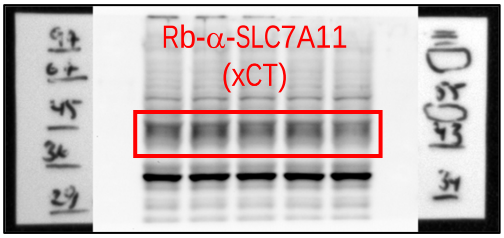

A549_xCT#30-01-23ab_Tricine_
Round2[r],15-15. scan_raw

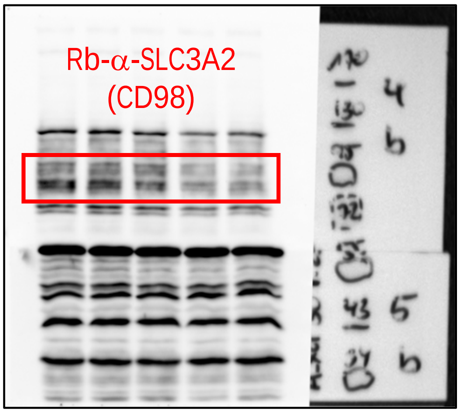

A549_xCT#30-01-23b_Gradient_
Round1,13-13. scan_raw

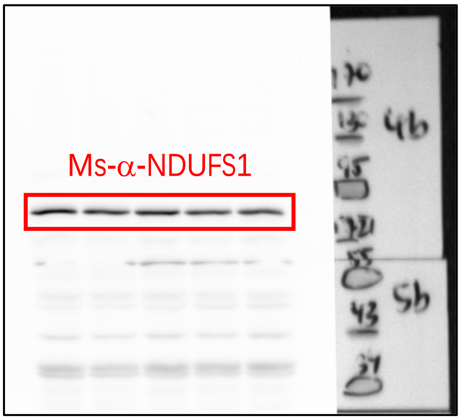

A549_xCT#06-02-23b_Gradient_
Round2,2-2. scan_raw

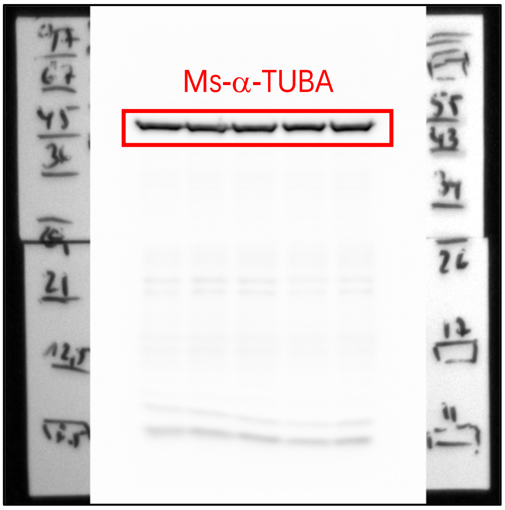

A549_xCT#06-02-23b_Tricine_
round3,1-1. scan_raw

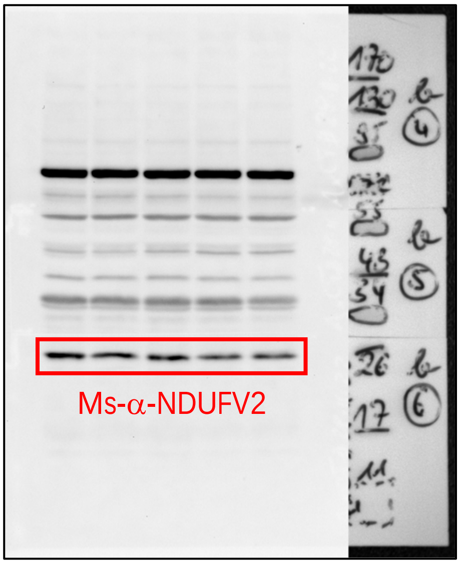

A549_xCT#28-11-22b_Gradient_
Round2,15-15. scan_raw

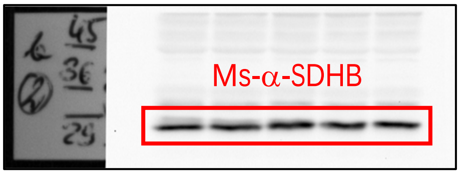

A549_xCT#28-11-22b_Gradient_
Round3,15-15. scan_raw

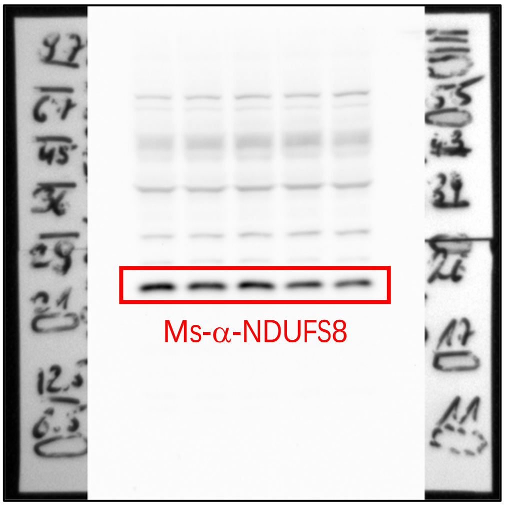

A549_xCT#28-11-22b_Round2,8-8. scan_raw

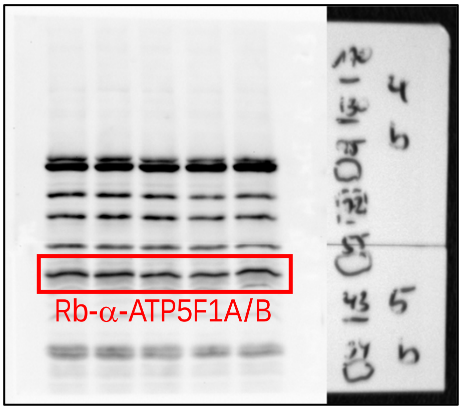

A549_xCT#30-01-23b_Gradient_
Round4,15-15. scan_raw

SourceData_ExtFig9eghk_ right(wo)

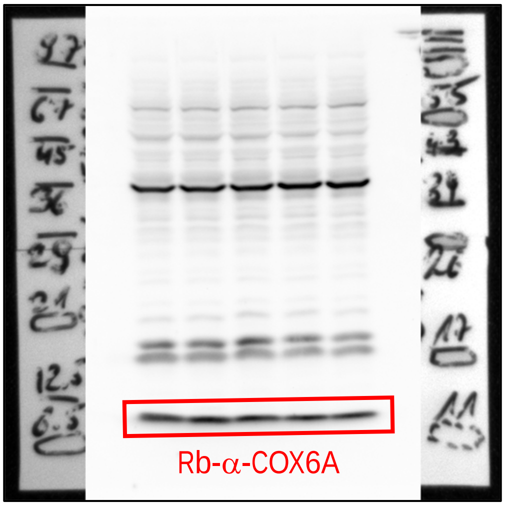

A549_xCT#28-11-22b_Round1,4-4. scan_raw

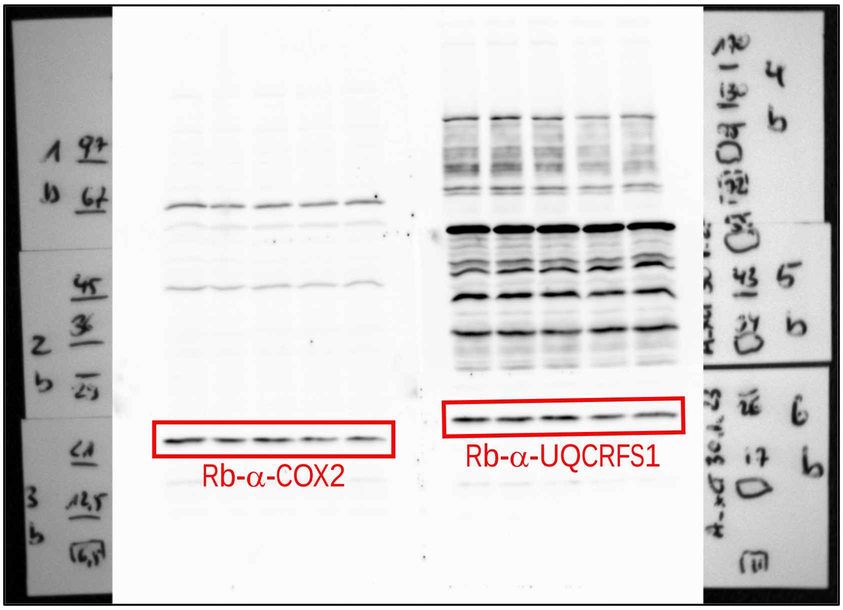

A549_xCT#30-01-23b_Gradient_Round1,7-7. scan_raw

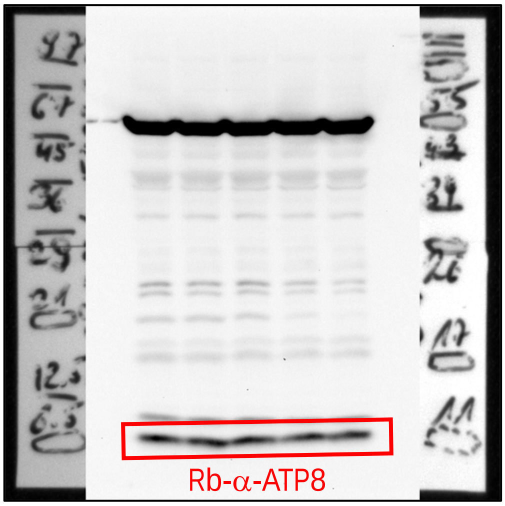

A549_xCT#28-11-22b_ Round3,15-15. scan_raw

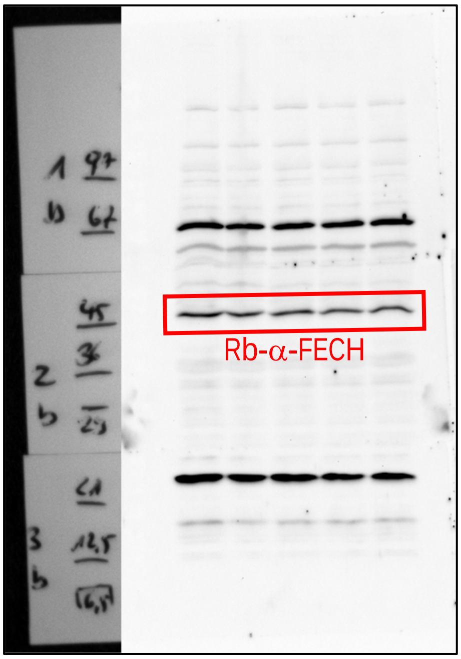

A549_xCT#30-01-23b_Gradient_ Round1,15-15. scan_raw

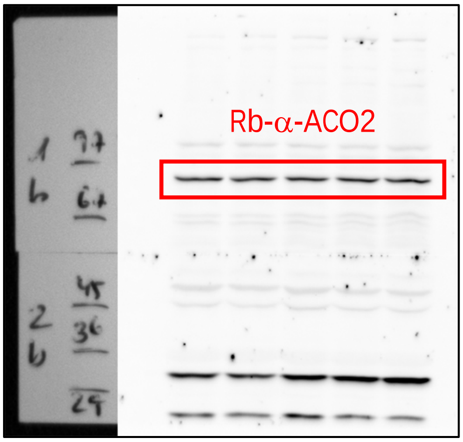

A549_xCT#06-02-23b_Gradient_ Round4,8-8. scan_raw

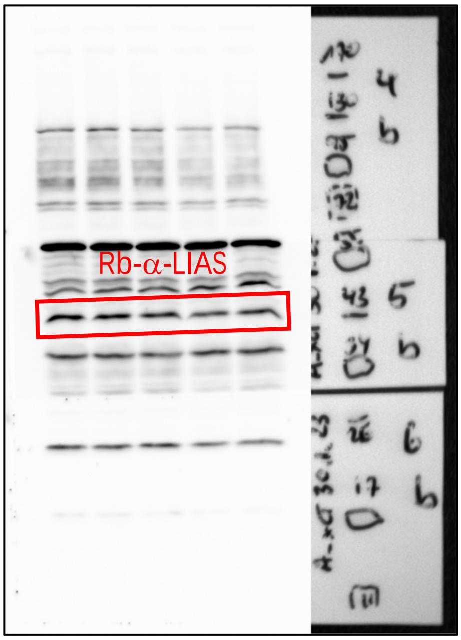

A549_xCT#30-01-23b_Gradient_ Round1,5-5. scan_raw

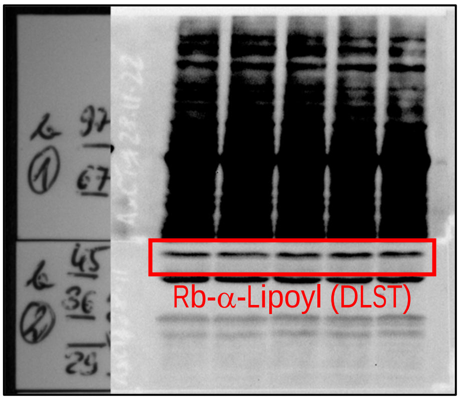

A549_xCT#28-11-22b_Gradient_ Round2,15-15. scan_raw

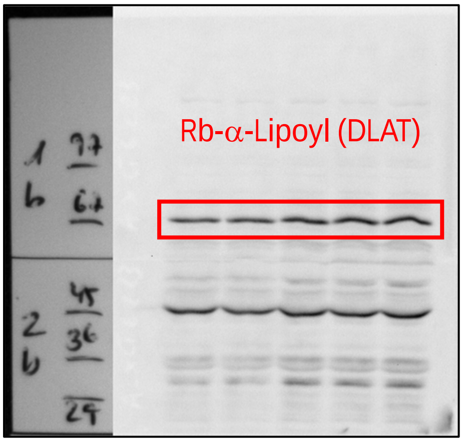

A549_xCT#06-02-23b_Gradient_ Round1,15-15. scan_raw

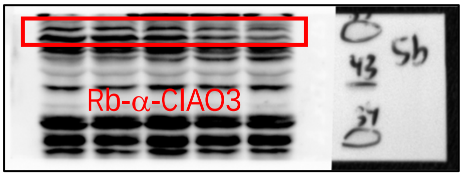

A549_xCT#06-02-23b_Gradient_ Round3,15-15. scan_raw

SourceData_ExtFig9eghk_right(wo)

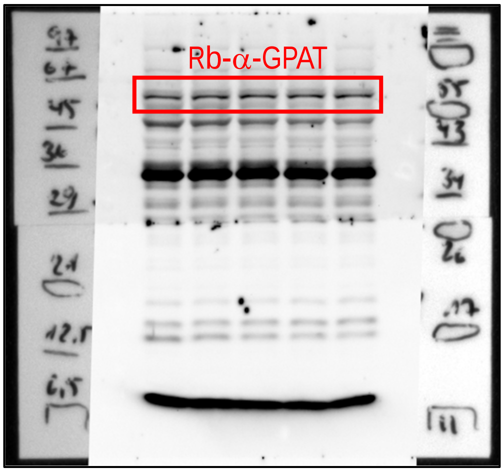

A549_xCT#30-01-23b_Tricine_
Round1,14-14. scan_raw

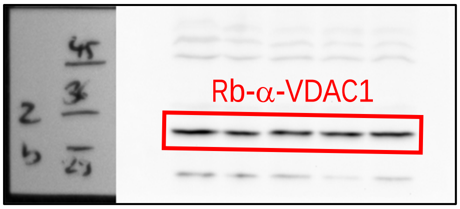

A549_xCT#30-01-23b_Gradient_
Round4,15-15. scan_raw

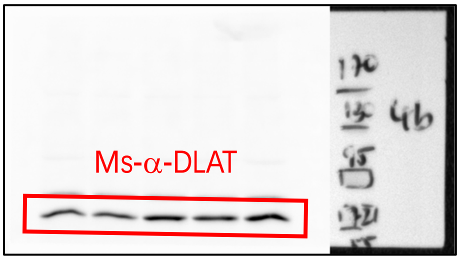

A549_xCT#06-02-23b_Gradient_
Round3.1,1-1. scan_raw

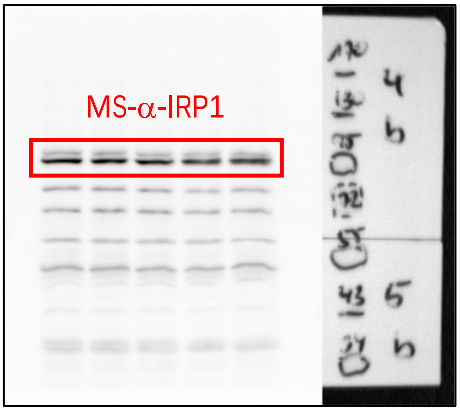

A549_xCT#30-01-23b_Gradient_
Round4,8-8. scan_raw

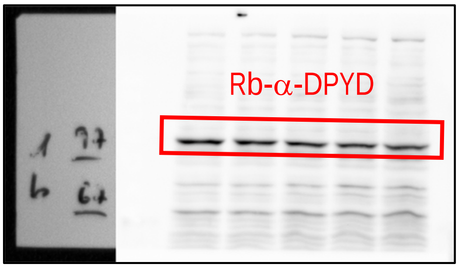

A549_xCT#06-02-23b_Gradient_
Round3,5-5. scan_raw

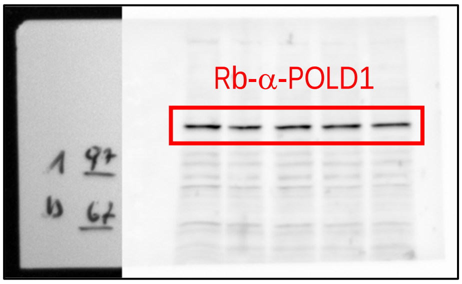

A549_xCT#30-01-23ab_Gradient_
Round5(WDH),3-3. scan_raw

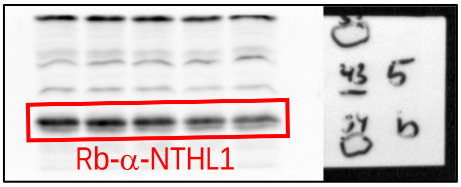

A549_xCT#30-01-23ab_Gradient_
Round2[r],15-15. scan_raw
